# Radiation-induced YAP/TEAD4 binding confers non-small cell lung cancer radioresistance via promoting NRP1 transcription

**DOI:** 10.1038/s41419-024-07017-6

**Published:** 2024-08-26

**Authors:** Mingwei Wang, Junxuan Yi, Hui Gao, Xinfeng Wei, Weiqiang Xu, Mingqi Zhao, Mengdie Zhao, Yannan Shen, Zhicheng Wang, Ning Wu, Wei Wei, Shunzi Jin

**Affiliations:** 1 NHC Key Laboratory of Radiobiology, School of Public Health, Jilin University, Changchun, Jilin China; 2 Department of Orthopedics, The First Hospital of Jilin University, Changchun, Jilin China; 3 Department of Radiation Oncology, China-Japan Union Hospital of Jilin University, Changchun, China; 4 Department of Radiotherapy, Chinese PLA General Hospital, Beijing, China

**Keywords:** Non-small-cell lung cancer, Cell migration

## Abstract

Despite the importance of radiation therapy as a non-surgical treatment for non-small cell lung cancer (NSCLC), radiation resistance has always been a concern, due to poor patient response and prognosis. Therefore, it is crucial to uncover novel targets to enhance radiotherapy and investigate the mechanisms underlying radiation resistance. Previously, we demonstrated that NRP1 was connected to radiation resistance in NSCLC cells. In the present study, bioinformatics analysis of constructed radiation-resistant A549 and H1299 cell models revealed that transcription coactivator YAP is a significant factor in cell proliferation and metastasis. However, there has been no evidence linking YAP and NRP1 to date. In this research, we have observed that YAP contributes to radiation resistance in NSCLC cells by stimulating cell proliferation, migration, and invasion. Mechanistically, YAP dephosphorylation after NSCLC cell radiation. YAP acts as a transcription co-activator by binding to the transcription factor TEAD4, facilitating TEAD4 to bind to the NRP1 promoter region and thereby increasing NRP1 expression. *NRP1* has been identified as a new target gene for YAP/TEAD4. Notably, when inhibiting YAP binds to TEAD4, it inhibits NRP1 expression, and Rescue experiments show that YAP/TEAD4 influences NRP1 to regulate cell proliferation, metastasis and leading to radiation resistance generation. According to these results, YAP/TEAD4/NRP1 is a significant mechanism for radioresistance and can be utilized as a target for enhancing radiotherapy efficacy.

## Introduction

Lung cancer is the second most prevalent malignant tumor, with non-small cell lung cancer (NSCLC) accounting for 85% of all cases [1–3]. Currently, lung cancer is treated using a variety of therapeutic approaches such as surgical resection, radiation, targeted therapy, ADC, and so on [1]. However, 77% of all lung cancer patients have evidence-based radiation indications, making radiotherapy one of the most important treatments for patients with advanced lung cancer that cannot be treated surgically [4–6]. However, the effectiveness of radiation therapy is significantly limited due to the NSCLC cells’ inherent or acquired radiation resistance [7–9]. Therefore, unveiling the mechanism underlying radiation resistance and identifying the potential biomarkers could improve the effectiveness for radiation therapy.

Yes-associated protein (YAP) is the downstream effector for the Hippo signaling pathway crucial in controlling cell growth, organ development, and tissue homeostasis [10, 11]. Abnormally activated or overexpressed YAP leads to tumor development, metastasis, and drug resistance in various cancers [12–15], While, inactive Hippo signaling pathway results in YAP/TAZ’s entry into the nucleus and act as a transcription coactivator. Since YAP cannot directly bind to DNA, it likely interacts with the downstream transcription factors [16, 17]. The TEA domain (TEAD) family is a transcription factor that is commonly associated with YAP. It consists of TEAD1–4 and is widely expressed in different mammalian tissues [18]. It regulates tumor metastasis, drug resistance, and other aspects of cancer by receiving signals via the Hippo signaling pathway.

Tumor radiation resistance is influenced by abnormal YAP expression. In glioma cells, YAP is responsible for promoting the expression of fibroblast growth factor 2, activating the MAPK-ERK signaling pathway, repairing the damaged DNA, and enhancing radiation resistance [19]. In esophageal cancer, radiation resistance is promoted and CDK6 expression is increased by YAP overexpression [15]. However, it is still unclear how YAP affects radiation resistance in NSCLC cells.

Neuropilin-1 (NRP1) is a glycoprotein that is present in all nuclear tissues and performs multiple biological functions [20, 21] as such angiogenesis, tumor development, and immune system function [22, 23]. In previous studies, we found that the expression levels of NRP1 and epithelial-mesenchymal transition (EMT) related proteins were significantly increased, and the PI3K/AKT/mTOR signaling pathway was also activated in radiation-resistant NSCLC cells [24, 25]. We hypothesized that NRP1 may contribute to the formation of radiation resistance in tumor cells.

In the present study, bioinformatics analyses results revealed that YAP is a crucial factor in NSCLC radiation resistance. Further research indicated that YAP lost its phosphorylation after irradiation and entered the nucleus where it bound with transcription factor TEAD4, thus contributing to radiation resistance enhancement by promoting NRP1 expression and NSCLC cell proliferation, migration, and invasion. NRP1 expression was inhibited by limiting YAP binding to TEAD4, which led to an increase in radiation sensitivity.

These results revealed a connection between YAP and radioresistance, implying that targeting the binding site of YAP and TEAD4 could be a feasible therapeutic strategy for NSCLC radiation.

## Materials and methods

### Cell culture

NSCLC cell lines (A549 and H1299) were obtained from Cell Bank Type Culture Collection of the Chinese Academy of Sciences (Shanghai, China). RPMI-1640 (Gibco, USA) was used to maintain the cell lines and culture them at 37 °C in a humidified atmosphere containing 5% CO_2_.

### Radiation treatment

Cells were subjected to 6 Gy irradiation at room temperature five times, totaling 30 Gy. The radiation dose for A549 cells was 1.02 Gy/min and 0.75 Gy/min for H1299 cells. The irradiation was carried out utilizing an X-ray generator (Model X-RAD320iX, USA).

### Patients and specimens

Surgical samples were obtained from five patients with lung adenocarcinoma who had surgery at the China–Japan Union Hospital of Jilin University between 2019 and 2020. The samples contained both tumor and adjacent normal tissue. No chemotherapy or radiotherapy was administered to patients before surgery. Liquid nitrogen was used immediately to freeze all tissue samples. All participants were informed about the study in writing by the Ethics Committee of the China–Japan Union Hospital of Jilin University and gave their permission to participate in this research.

### NSCLC cell-derived xenograft model and in vivo imaging analysis of SCID mice

Female SCID mice (4 weeks, 20 g) were purchased from Huafukang Biotechnology (Beijing, China). The mice were maintained in a particular pathogen-free facility at the Experimental Animal Center at the School of Public Health, Jilin University, where they enjoyed unrestricted food availability and a climate-controlled environment ranging from 22 °C to 25 °C with approximately 50% humidity. Animal ethics approval was from School of Public Health, Jilin University Animal Ethics Committee (Approval No. 2023-06-001). SCID mice were inoculated with A549 (NC/*YAP*^*KD*^) and A549-RR (NC/*YAP*^*KD*^) cells to establish a cell-derived xenograft model. The right hind legs of SCID mice were injected subcutaneously with 1 × 10^7^ cells in PBS. Lead plates were placed outside the tumor to reduce collateral damage. Provided at a dosage of 2.0 Gy/min, the total dose of radiation was fixed at 20 Gy. Tumor size was measured every two days after irradiation. A device from Xenogeny IVIS Spectrum (Caliper, USA) was used to image green fluorescent protein fluorescence in SCID mice after transplantation (14 days after irradiation). The mice were euthanized at the end of the experiment to minimize suffering following the protocols approved by the Jilin University’s Ethics Committee, which adhered to the Rules for the Management of Laboratory Animals. *V* = 0.5 × *ab*^2^ was used to calculate the tumor volume, where ‘*a*’ represented the longest diameter and ‘*b*’ represented the shortest diameter.

## Results

### YAP is overexpressed in lung adenocarcinoma tissues and radioresistant NSCLC cells

**Fig. 1:**
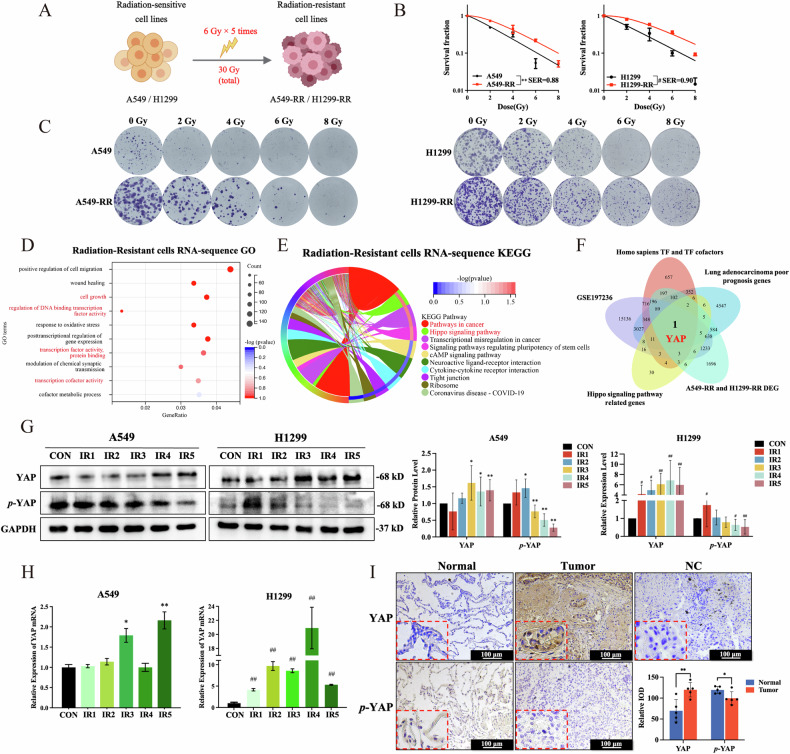
The transcriptional coactivator YAP is substantially expressed in radiation-resistant NSCLC cells. **A** Pattern diagram for A549-RR and H1299-RR models. **B** Quantitative results and **C** representative images of colonies of parental and RR cells after radiation treatment (*n* = 3). **D** GO analysis of differentially expressed genes. **E** KEGG pathway evaluation of genes that differ in expression levels. **F** Venn diagrams for five databases. **G** YAP and p-YAP protein levels (*n* = 3). **H** *YAP* mRNA levels (*n* = 3). **I** YAP-positive and p-YAP-positive IHC staining of human lung adenocarcinoma tumors (100 μm, *n* = 5). Mean ± SD. **p < 0.01 *vs*. A549/Normal, *p < 0.05 *vs*. A549/Normal, ##*p* < 0.01 *vs*. H1299, and #*p* < 0.05 *vs*. H1299.

Our study focused on the mechanism of radiation resistance in NSCLC by selecting human A549 and H1299 NSCLC cells and generating radiation-resistant cell models A549-RR and H1299-RR (Fig. 1A). Colony formation experiments were performed to validate the creation of a radiation-resistant cell model. In the process, it was discovered that radioresistance cells (RR) were more resistant to radiation (Fig. 1B, C). Furthermore, optical microscopy observations showed a notable increase in RR cell size and pseudopodia elongation (Suppl. Fig. 1A). Flow cytometry results revealed that RR cell apoptosis rates were much lower compared to parental cells after 10-Gy irradiation (Suppl. Fig. 1B). All of these findings pointed to the effective creation of an NSCLC cell model that is resistant to radiation.

The molecular mechanisms underlying radiation resistance in NSCLC were investigated using RNA sequencing. GO analysis showed that DEGs had a major impact on the function of transcription factors and cofactors (Fig. 1D). KEGG analysis revealed that numerous signaling pathways were altered when radiation resistance developed, with the Hippo signaling pathway being the most significantly altered (Fig. 1E). To identify the crucial molecules affecting radiation resistance, DEGs from the GSE197236 dataset, DEGs from RR cells in our RNA sequencing, known transcription factors and cofactors, the Hippo signaling pathway-related genes, and genes linked to the poor prognosis in lung adenocarcinoma were studied. *YAP* was identified as the only transcription coactivator gene (Fig. 1F).

Expression of YAP gene was further determined and found that YAP mRNA and protein levels greatly rose during the creation of RR cells and remained high in the RR cells (Fig. 1G, H). It is well known that YAP must be dephosphorylated to enter the nucleus and act as a transcription coactivator [10, 26]. Therefore, *p*-YAP expression was examined and found to gradually decrease as irradiation times increased (Fig. 1G). Isolation and detection of cytoplasmic and nuclear proteins from parental and RR cells demonstrated a considerable rise in YAP levels in the nucleus (Suppl. Fig. 1C). These findings demonstrated that nuclear YAP penetration increased significantly as radiation resistance progressed. In addition, five individuals with lung adenocarcinoma had their YAP and *p*-YAP expression detected using immunohistochemistry in tumor samples and adjacent normal tissues. These results showed that tumor tissues expressed more YAP and less p-YAP than did normal tissues (Fig. 1I and Suppl. Fig. 1D). Taken together, the above results showed that YAP, a key gene in radiation resistance, is highly expressed in RR cells.

### YAP promotes proliferation, invasion, and migration of NSCLC cells

**Fig. 2:**
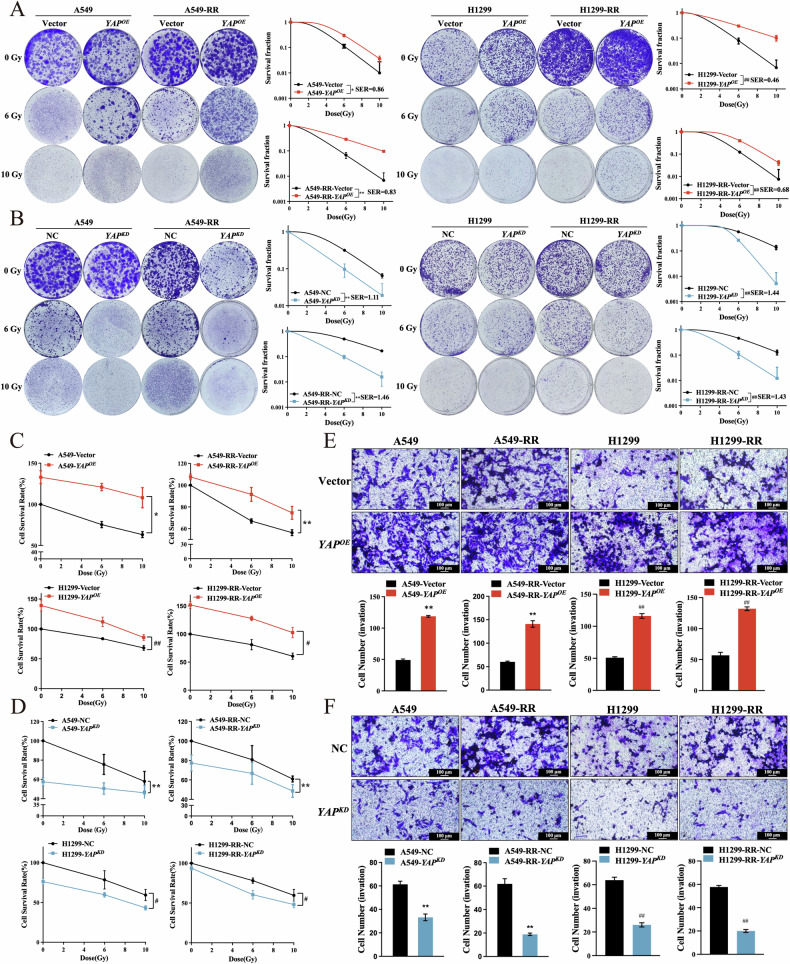
YAP increases NSCLC cell proliferation, invasion, and migration. **A**, **B** Quantitative results and representative images for colony formation assay. **C**, **D** The CCK-8 test was used to measure *YAP*^*KD*^ and *YAP*^*OE*^ viability and proliferation. **E**, **F** Transwell assay in H1299 and A549 cells (100 μm). Mean ± SD, *n* = 3. **p < 0.01 *vs*. Vector/NC, *p < 0.05 *vs*. Vector/NC, ##*p* < 0.01 *vs*. Vector/NC, and #*p* < 0.05 *vs*. Vector/NC.

In order to confirm YAP’s involvement in NSCLC cells’ development of radiation resistance, *YAP* knockdown and overexpression plasmids (*YAP*^*OE*^ & *YAP*^*KD*^) were generated. *YAP* levels were then reduced and increased in H1299 and A549 cell lines using lentivirus-mediated infection followed by clone formation experiments. Compared to the Vector/NC group, the high YAP expression group had significantly higher colony formation than the low YAP expression group (Fig. 2A, B). Thus, YAP has the potential to influence radiation resistance generation.

RNA sequencing GO analysis results showed that differential genes are substantially related to cell proliferation and migration (Fig. 1D and Suppl. Fig. 1C). Thus, further investigation was done on how YAP affected invasiveness, migration, and proliferation. The findings showed that these abilities were enhanced in the presence of overexpressed YAP and inhibited in the absence of YAP (Fig. 2C–F and Suppl. Fig. 2A–D). Based on these results, YAP can increase the proliferation, migration, and invasion capacity of NSCLC cells, thereby improving their resistance to radiation.

### YAP regulates NRP1 transcription and translation

**Fig. 3:**
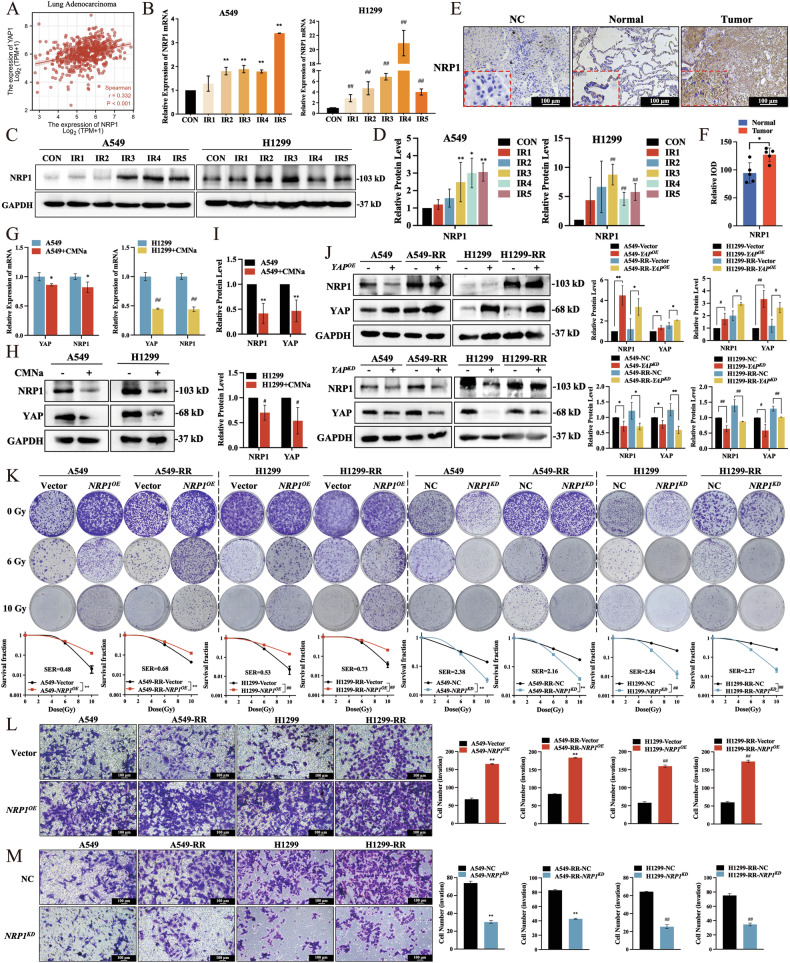
NRP1 transcription and translation are regulated by its transcriptional coactivator YAP. **A** Correlation between YAP and NRP1 in lung adenocarcinoma based on TCGA databases. NRP1 **B** mRNA and **C**, **D** protein levels (*n* = 3). **E**, **F** NRP1-positive IHC staining of human lung adenocarcinoma tumors (100 μm, *n* = 5). YAP and NRP1 (**G**) mRNA and (**H**, **I**) protein levels after adding radiation sensitizer CMNa (1 mM, *n* = 3). **J** Representative immunoblots using indicated antibodies in A549 and H1299 cells (*n* = 3). **K** Quantitative results and representative images of colony formation assay (*n* = 3). **L**, **M** Transwell assay after knockdown and overexpression of *NRP1* in H1299 and A549 cells (100 μm, *n* = 3). Mean ± SD. **p < 0.01 *vs*. Vector/NC/Normal, *p < 0.05 *vs*. Vector/NC/Normal, ##*p* < 0.01 *vs*. Vector/NC, and #*p* < 0.05 *vs*. Vector/NC.

Our previous report demonstrated that radiation resistance development in NSCLC is directly related to NRP1 [25]. In the present study, the TCGA databases were analyzed in order to investigate the connection between YAP and NRP1, r showing a positive connection between YAP and NRP1 in lung adenocarcinoma patients (Fig. 3A). During the generation of RR cells, NRP1 mRNA and protein were stably expressed after the fifth irradiation procedure (Fig. 3B–D). NRP1 was highly expressed in lung cancer tissue samples from five patients compared to normal lung tissue samples (Fig. 3E, F and Suppl. Fig. 3A).

Colony formation assay showed that radiosensitivity was enhanced when radiosensitizer sodium glycididazole (CMNa) was added to parental cells (Suppl. Fig. 2E, F). YAP and NRP1 levels were decreased in radiation-sensitive cells (Fig. 3G–I). Based on these results, it appears that YAP and NRP1 are essential for the development of radiation resistance. The relationship between YAP and NRP1 was examined by observing changes in NRP1 when *YAP* was knocked down or overexpressed. Overexpression of *YAP* enhanced NRP1 mRNA and protein levels, while knockdown of *YAP* decreased them (Fig. 3J and Suppl. Fig. 2G, H). In addition, YAP level did not change significantly when *NRP1* was knocked down or overexpressed, suggesting that YAP is an upstream regulator of NRP1 transcription and translation (Suppl. Fig. 3B, C).

The expression of *NRP1* was upregulated and knocked down in order to further elucidate its function in NSCLC radiation resistance. The results of the colony formation assay results demonstrated that *NRP1* overexpression increased radiation resistance of parental cells. *NRP1* knockdown resulted in increased radiation sensitivity of RR cells (Fig. 3K). In addition, consistent with the YAP results, *NRP1* knockdown/overexpression decreased/enhanced cell proliferation, migration, and invasion of NSCLC cells (Fig. 3L, M and Suppl. Fig. 3D–H). Based on these results, YAP is essential for the development of radiation resistance as it controls NRP1 transcription and translation.

### *NRP1* is a novel target gene for YAP/TEAD4

**Fig. 4:**
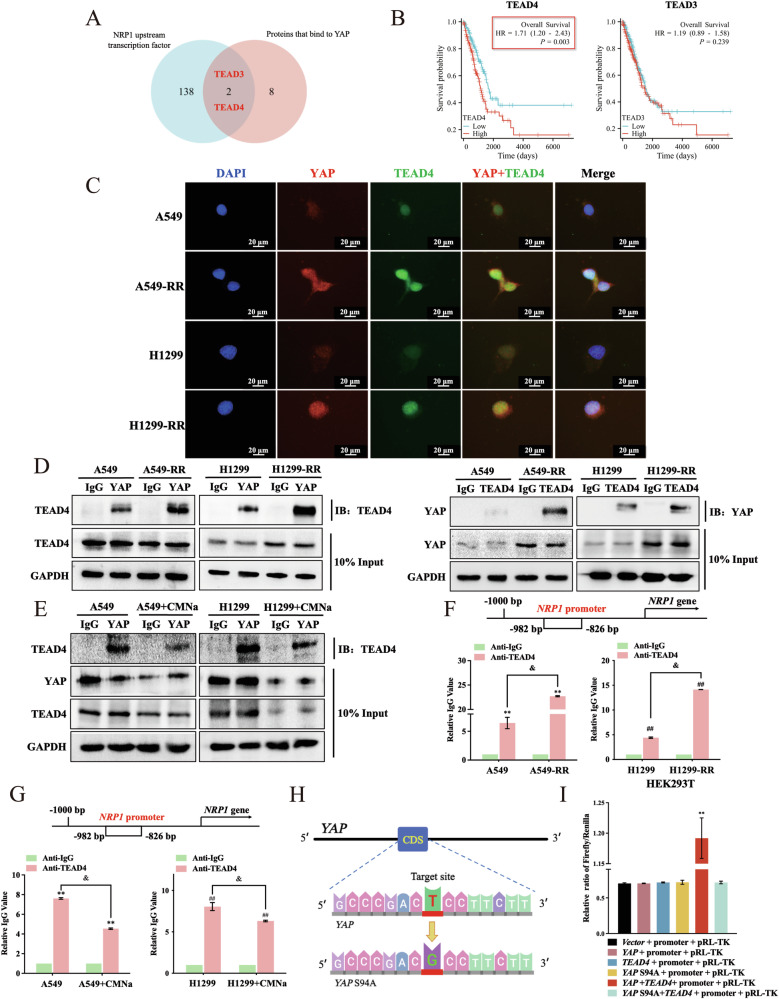
*NRP1* is a new gene that targets YAP/TEAD4. **A** Venn diagrams for two databases. **B** Kaplan–Meier analysis in patients with lung adenocarcinomas that express TEAD3 and TEAD4 at high and low levels. **C** Representative illustration of IF cell staining for YAP (red) or TEAD4 (green) and cell nuclei staining for DAPI (blue) (20 μm). **D**, **E** Western blot analysis of YAP co-IP fractions for its ability to immunoprecipitate with TEAD4. **F**, **G** ChIP-qPCR results using TEAD4 antibody. **H** Pattern diagram for mutant plasmid YAP S94A construction. **I** Several plasmids were added to HEK293T cells in combination with the NRP1 luciferase reporter. The luciferase activity was assessed using a dual-luciferase reporter system. Mean ± SD, *n* = 3. **p < 0.01 *vs*. Vector/IgG, ##*p* < 0.01 *vs*. Vector/ IgG.

Based on those findings, more research was done on the mechanism by which YAP controls NRP1. It is a well-known that YAP is a transcription coactivator that does not have a domain directly binding to DNA. Therefore, it likely binds to transcription factors to function [27, 28]. Additional analysis was explored on whether YAP acts as a transcriptional coactivator of *NRP1* and what transcription factor is in between. Cross-referencing analysis revealed two genes (TEAD3 and TEAD4) that intersected the transcription factors *NRP1* and YAP (Fig. 4A). The TCGA database study found that people with lung cancer had a higher TEAD4 overexpression and a poorer overall survival rate (Fig. 4B). In an attempt to confirm the relationship between YAP and TEAD4, immunofluorescence co-staining showed that RR cells had much higher levels of TEAD4 and YAP expression, providing a spatial basis for their interaction (Fig. 4C). RR cells had significantly higher binding rates than parental cells in the co-IP experiments (Fig. 4D). Addition of the radiation sensitizer CMNa decreased the binding between YAP and TEAD4 (Fig. 4E).

Next, JASPAR and ChIP-qPCR were used to predict transcription factor binding sites. As a result, TEAD4 bound to the *NRP1* promoter region (−982 to −826 bp; Fig. 4F). This binding was reduced in radiation-sensitive cells (Fig. 4G). Then, we construct mutant plasmid YAP S94A (Fig. 4H). The luciferase reporter assay showed that *NRP1* was only positively regulated when YAP and TEAD4 were present simultaneously. Interestingly, positive regulation disappeared when YAP and TEAD4 binding was inhibited (Fig. 4I). Thus, the above results demonstrated that NRP1 is a target gene for YAP/TEAD4, suggesting that *NRP1* function regulation may requires both YAP and TEAD4.

### YAP/TEAD4 is an indispensable transcription factor for NRP1

**Fig. 5:**
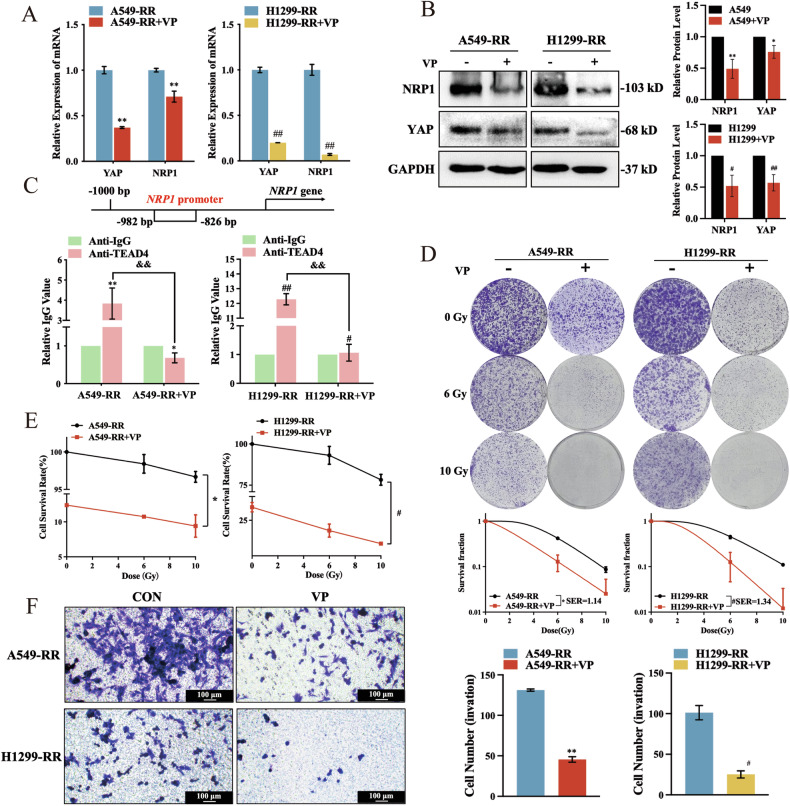
YAP/TEAD4 is necessary for NRP1 to function as a transcription factor. **A** *YAP* and *NRP1* mRNA levels with verteporfin (VP) treatment (5 mg/L). **B** YAP and NRP1 protein levels with VP treatment. **C** ChIP-qPCR results using TEAD4 antibody after VP treatment. **D** Quantitative results and representative images of colony formation assay after VP treatment. **E** The CCK-8 assay was used to determine the survival and growth of cells after VP treatment. **F** Transwell assay after VP treatment (100 μm). Mean ± SD, *n* = 3. **p < 0.01 *vs*. A549-RR, *p < 0.05 *vs*. A549-RR, ##*p* < 0.01 *vs*. H1299-RR, and #*p* < 0.05 *vs*. H1299-RR.

To validate the hypothesis that YAP and TEAD4 must work together to regulate NRP1, verteporfin (VP), which specifically inhibits YAP binding to TEAD4, was added to RR cells. As a result, the NRP1 mRNA and protein levels were reduced (Fig. 5A, B). When YAP was not binding to TEAD4, TEAD4 was found not to bind to the NRP1 promoter region by ChIP-qPCR analysis (Fig. 5C). At the same time, following VP treatment, there was a significant reduction in the radiation resistance, cell proliferation, migration, and invasion capacity of RR cells (Fig. 5D–F and Suppl. Fig. 3I). When radiation sensitivity increased, the proliferation ability of cells also decreased significantly (Suppl. Fig. 4A). In addition, NRP1 expression levels did not change whether *TEAD4* was overexpressed or shut down (Suppl. Fig. 4B–E). Based on these results, it can be concluded that YAP is the functional regulator of NRP1, and that TEAD4 is the structural binder for the NRP1 promoter region.

### NRP1 mediates the radioresistant effect of YAP on NSCLC

**Fig. 6:**
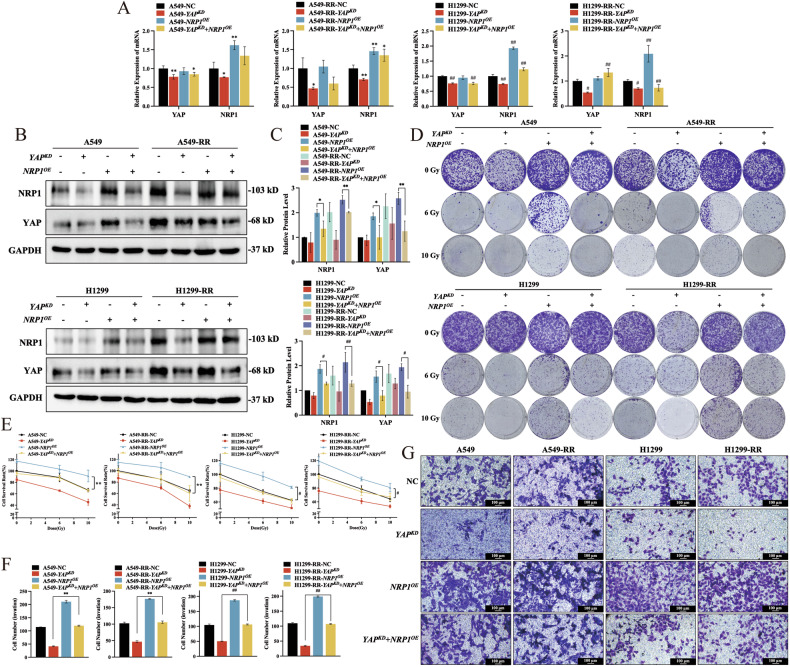
The radioactive effects of YAP are mediated by NRP1 in NSCLC. **A** *YAP* and *NRP1* mRNA levels after *YAP* silencing or *NRP1* overexpression. **B**, **C** YAP and NRP1 protein levels after *YAP* silencing or *NRP1* overexpression. **D** Representative images and quantitative results (Table S4) for colony formation assay after *YAP* silencing or *NRP1* overexpression. **E** The CCK-8 test was used to assess cell viability and proliferation after *YAP* silencing or *NRP1* overexpression. **F**, **G** Transwell assay results after *YAP* silencing or *NRP1* overexpression (100 μm). Mean ± SD, n = 3. **p < 0.01 *vs*. NC, *p < 0.05 *vs*. NC, ##*p* < 0.01 *vs*. NC, and #*p* < 0.05 *vs*. NC.

A rescue experiment was performed to determine whether YAP/TEAD4 truly affects the development of NSCLC radiation resistance via NRP1. First, *YAP* levels were decreased while increasing *NRP1* levels in both parental and RR cells. It was observed that *YAP* suppression inhibited the development of radiation resistance, and that it also prevented cell invasion, migration, and proliferation (Fig. 6A–D and Table S3). It is worth noting that *NRP1* overexpression can partially reverse the effects of *YAP* knockdown, counteracting the effect of *YAP* enhancing radiation sensitivity (Fig. 6E, F and Suppl. Fig. 5A, B). The opposite result was achieved by overexpressing *YAP* and knocking down *NRP1* (Suppl. Fig. 4F–I and Suppl. Fig. 5C–E). Based on these outcomes, it can be suggested that the interaction between YAP and NRP1 is essential for NSCLC radiation resistance development. YAP influences the cells’ ability to proliferate, migrate, and invade.

### *YAP* gene depletion reverses radiation resistance and inhibits tumor development in vivo

**Fig. 7:**
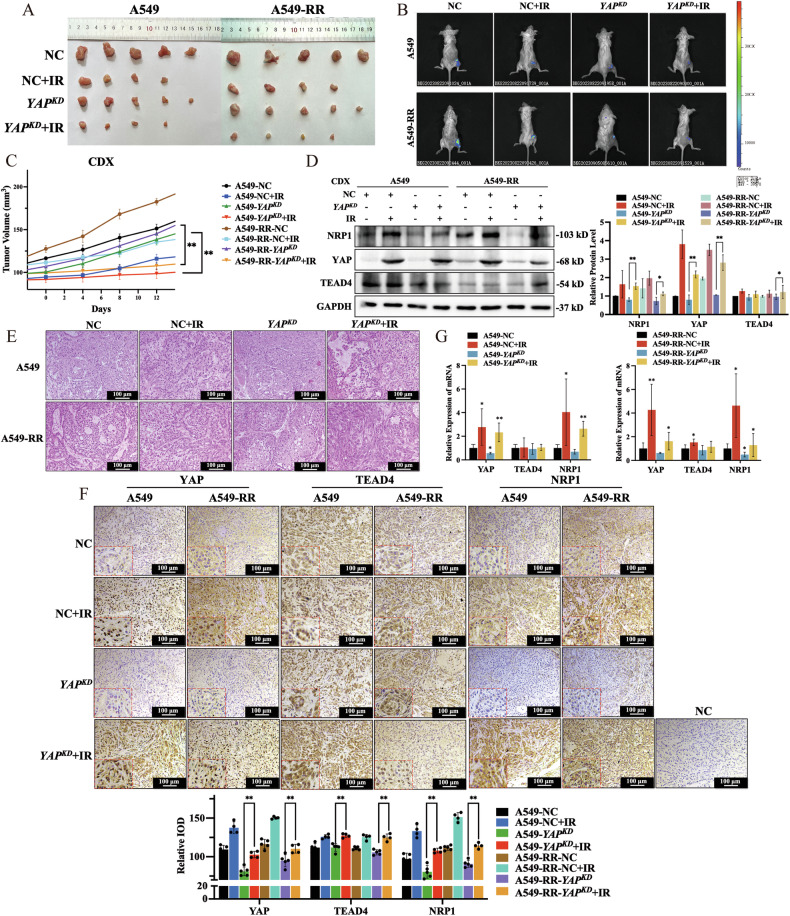
*YAP* gene depletion reverses radiation resistance and inhibits tumor development in vivo. **A** A549 and A549-RR xenograft volumes were compared among eight lung cancer groups. **B** Representative pseudo color bioluminescence images of xenografts 14 days post-irradiation. **C** Relative tumor size was measured every 2 days. **D** YAP, TEAD4 and NRP1 protein levels in SCID mice. **E** Paraffin-embedded tumor tissue slices in SCID mice stained with H&E (100 μm). **F** YAP-, TEAD4- and NRP1-positive IHC staining in SCID mice tumor tissue (100 μm). **G** *YAP*, *TEAD4* and *NRP1* mRNA levels in SCID mice tissue. Mean ± SD, *n* = 4 or 5. **p < 0.01 *vs*. NC, *p < 0.05 *vs*. NC.

To create a tumor model in SCID mice and determine the importance of YAP, A549 and A549-RR cells that were stalely transfected with shRNA or NC were utilized. Treatment of SCID mice with or without 20 Gy radiation showed that IR elicited a reduction in the volume of xenografts, especially in A549 cells (Fig. 7A, B). Notably, *YAP* knockdown caused a further reduction in xenograft volume post-IR (Fig. 7C). Hematoxylin and eosin staining showed the characteristic features of adenocarcinoma, such as glandular cavity and mucus. Foam cells and large necrotic tissues were noted in the group received radiation (Fig. 7E). Results from immunohistochemistry and western blotting showed a lower NPR1 expression in the YAP knockdown group. TEAD4 expression was increased after irradiation, regardless of whether YAP was knocked down. Moreover, the nucleus showed a noticeable rise in YAP, while both the cytoplasm and nucleus showed a substantial increase in NRP1 after irradiation (Fig. 7D, F). The mRNA changes were consistent with protein changes (Fig. 7G). Therefore, *YAP* knockdown can reverse the progression of NSCLC radiation resistance and prevent tumor growth in vivo.

**Fig. 8:**
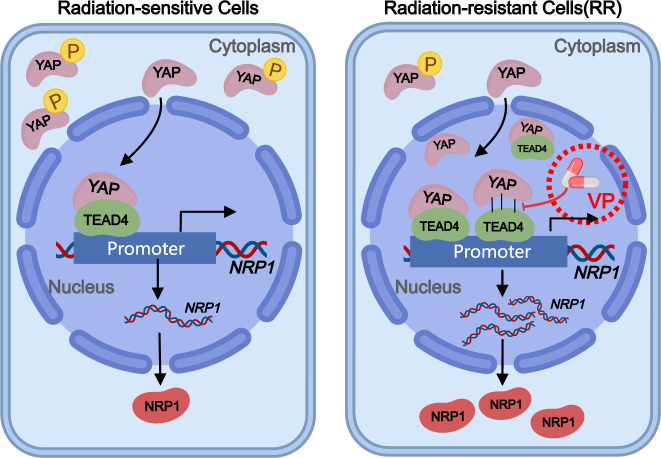
A working model for radiation resistance regulation in NSCLC cells via the YAP/TEAD4/NRP1 axis. Created with MedPeer (www.medpeer.cn).

In conclusion, our results revealed an important mechanism for radiation resistance development in NSCLC. Radiation causes YAP dephosphorylation, increases its entry into the nucleus, and binds to TEAD4 in NSCLC cells. YAP and TEAD4 are both play the role in transcription and bind to the *NRP1* promoter region, which positively impacts NRP1 expression (Fig. 8). NRP1 transcription and translation can be inhibited by blocking YAP binding to TEAD4. Ultimately, enhancing or inhibiting NSCLC cells’ ability to grow, migrate, and invade can either develop or reverse radiation resistance.

Taken together, our results reveal the connection between the YAP/TEAD4/NRP1 axis and radiation resistance in NSCLC cells. These findings suggest that inhibiting YAP binding to TEAD4 or inhibiting YAP expression may have therapeutic value for lung cancer patients as it can restore radiation sensitivity of lung cancer cells.

## Discussion

Radiotherapy is the primary treatment for NSCLC. However, after radiotherapy, tumor cells develop radiation resistance, rendering them insensitive to ionizing radiation. Therefore, exploring the mechanisms behind the development of radiation resistance and identifying novel therapeutic targets are imperative [29, 30]. In this study, we observed that radiation induced YAP to lose phosphorylation and enter the nucleus, where it bound to the transcription factor TEAD4. In addition, our research findings indicate that NRP1, as a novel target of YAP/TEAD4, can regulate the development of radiation resistance in NSCLC cells. These findings not only show a novel molecular mechanism by which the YAP/TEAD4/NRP1 axis influences radiation resistance development, but also highlight the probable mechanism by which the YAP inhibitor verteporfin acts as a radiation sensitizer.

Firstly, we used A549 cells and H1299 cells to create a model of radiation-resistant resistant cells in order to clarify the process of producing radiation resistance in NSCLC. Then, RNA sequencing revealed the main roles of transcription factors, transcription coactivators, and the Hippo signaling pathway. Previous studies have shown, the ability of transcription factors and transcription coactivators to control a wide range of biological processes, including drug resistance, metastasis, and so on [31–33]. The Hippo signaling system is also intimately linked to radiosensitivity. For example, ubiquitin-specific peptidase 21 can activate YAP via controlling FOXM1 stability, blocking Hippo signaling, lowering cell proliferation, causing apoptosis, and eventually increasing the radiosensitivity of cervical cancer cells [34]. Intersecting by Venn diagram, the only mutated gene *YAP* was selected. Simultaneously, we analyzed YAP in human tumor samples and discovered high levels of YAP.

YAP is a key effector molecule of Hippo pathway and a transcriptional coactivator. In our investigations, increasing the irradiation dose resulted in a constant decline in p-YAP and an increase in the nucleus, indicating that YAP works as a transcription coactivator. Thus, to investigate the function of YAP in radiation resistance, YAP expression was manipulated in NSCLC cells. Our results show that high YAP expression can promote NSCLC cell growth and metastasis, increasing radiation resistance. Since prior findings revealed that NRP1 plays an important role in radiation resistance [24, 33, 35]. And many studies have shown that YAP interacts with TEAD4 as a heterodimer that binds to downstream target genes [12, 36]. To further investigate the tripartite link between YAP, TEAD4, and NRP1, we conducted IP, ChIP-qPCR and Plasmid transfection and luciferase reporter assays. It was discovered that YAP directly binds to TEAD4, enriches TEAD4 binding to the *NRP1* promoter region, and stimulates NRP1 translation and transcription. When we inhibit YAP binding to TEAD4 with Verteporfin, TEAD4 loses its ability to connect to NRP1, reversing RR cell proliferation, migration, and invasion and improving radiation sensitivity. Combined with the fact that TEAD4 cannot regulate NRP1 expression suggests that YAP functionally regulates NRP1, while TEAD4 structurally binds to NRP1. Both are essential in regulating NRP1.

We performed reverse validation using radiosensitizers CMNa. The results showed decreased binding to the NRP1 promoter region and the level of YAP and NRP1, which led to a decrease in clone survival rate and cell viability. Positive and negative verification confirmed that YAP/TEAD4/NRP1 is crucial for radiation resistance. Finally, to clarify the mechanisms of action of YAP/TEAD4/NRP1, we created xenograft tumor SCID mice. We found that *YAP* knockdown in vivo led to slower tumor growth after irradiation and the expression of YAP and NRP1 dramatically increased. Ultimately, *YAP* knockdown partially reversed the radiation resistance developing in NSCLC.

In summary, the key mechanism by which YAP/TEAD4/NRP1 enhances radiation protection in NSCLC cells was determined by the current investigation. However, since the limitations of clinical treatment plans, specimens from NSCLC patients who were tolerant radiation before and after radiotherapy could not be collected. Thus, human specimens could not be verified further. Additionally, lung adenocarcinoma cells from NSCLC, the most frequent form of lung cancer, were selected to verify that YAP/TEAD4/NRP1 had an effect on radiation resistance in NSCLC cells but other lung cancer classification have not been well explored.

Our novel findings clarify an association between YAP/TAED4/NRP1 and radioresistance in NSCLC. Targeted medications have typically been used to inhibit tumor growth by altering gene expression, but they can also affect healthy cells. Thus, according to our findings, radiosensitivity can be improved by creating targeted medicines to restrict YAP binding to TEAD4 while preserving gene expression. It is a completely new perspective and method for increasing the efficacy of radiation.

### Supplementary information

Supporting Information

Original Western blots Data

## Data Availability

The data presented in this study are available on request from the corresponding author. The RNA-seq raw data has been submitted to the NCBI. The Bioproject accession is PRJNA1135543.
