# Supplementary material for: Radiation-induced YAP/TEAD4 binding confers non-small cell lung cancer radioresistance via promoting NRP1 transcription
Source: Cell Death Dis. 2024 Aug 26;15(8):619. doi: 10.1038/s41419-024-07017-6 (PMC11347582; doi:10.1038/s41419-024-07017-6)
Supplement: Supplementary file 1 — Supporting Information [file 41419_2024_7017_MOESM1_ESM.pdf]

Supporting Information

Radiation-induced YAP/TEAD4 binding confers non-small cell lung cancer radioresistance via promoting NRP1 transcription

Mingwei Wang[1], Junxuan Yi[1], Hui Gao[1, 2], Xinfeng Wei[1], Weiqiang Xu[1], Mingqi Zhao[1], Mengdie Zhao[1], Yannan Shen[1], Zhicheng Wang[1], Ning Wu[3], Wei Wei[4*], Shunzi Jin[1*].

1. NHC Key Laboratory of Radiobiology, School of Public Health, Jilin University, Changchun, Jilin, China
2. Department of Orthopedics, The First Hospital of Jilin University, Changchun, Jilin, China.
3. Department of Radiation Oncology, China-Japan Union Hospital of Jilin University, Changchun, China
4. Department of Radiotherapy, Chinese PLA General Hospital, Beijing, China

Corresponding author: Wei Wei[2*], Shunzi Jin[1*].

Email: jinsz@jlu.edu.cn; dr_weiwei528@163.com

Supplementary Methods

Plasmid transfection

Human pPLK/GFP+Puro-YAP shRNA (*YAP*^{KD}) and pPLK/GFP+Puro-TEAD4 shRNA (*TEAD4*^{KD}) and pCDH-CMV-TEAD4-GFP-Puro (*TEAD4*^{OE}) were constructed by Public Protein/Plasmid Library (Nanjing, China). pCDH-CMV-YAP-GFP-Puro (*YAP*^{OE}), pLNCX2-NRP1 (*NRP1*^{OE}) and RetroQ-NRP1 shRNA (*NRP1*^{KD}) were generated in-house. The Lipofectamine 2000 reagent (Invitrogen, USA) was used for transfections in accordance with the manufacturer's instructions.

Colony formation assay and Cell proliferation assay

For the colony formation assay, 500-1,000 cells were planted in six-well plates and exposed to varying radiation doses (0, 2, 4, 6, and 8 Gy) the next day. The cells were treated with 4% paraformaldehyde (LEAGENE, Beijing, China) and stained with 0.1% crystal violet (Solarbio, Beijing, China) 14 days later. Colonies per dish were then counted. Cell proliferation assay was performed as described previously(1).

Wound healing assay and Transwell assay

A six-well plate was filled with complete medium and cells were seeded and cultured for 24 h for the wound healing assay. Then, a straight line was scratched through the monolayer and the plate was photographed at 0 h, 24 h, and 48 h post scratching. Transwell assay was performed using Transwell membranes (Biofil, Guangzhou, China) with Matrigel (Corning, USA). A total of 4×105 cells in serum-free RPMI-1640 were added to the chambers, which were placed in 12-well plates containing 20% fetal bovine serum. The inserts were stained with crystal violet after treatment and incubated 30 min.

Quantitative real-time PCR (qRT-PCR)

qRT-PCR was performed as described previously (2).

Western blotting

Cells were washed with pre-cooled phosphate-buffered saline (PBS), lysed with reagents, and celllysate was quantitated using BCA detection kit (Beyotiome, Shanghai, China) to determine protein concentrations. After SDS-PAGE, the proteins were transferred to nitrocellulose membranes for Western blot analysis. After themembranes were blocked using 5% skim milk r in washing buffer, primary antibodies were used to immunoblot the membranes overnight at 4°C. The membranes were rinsed with washing buffer and incubated with secondary antibodies for 2 hours at room temperature. The membranes were analyzed using chemiluminescence (Pierce ECL kit, Thermo Fisher Scientific, USA) after washing with washing buffer. The relative gray

value of WB was analyzed through ImageJ software, and the result was expressed as the target band's gray value divided by the reference protein's gray value (n=3).

Chromatin immunoprecipitation-quantitative PCR (ChIP-qPCR)

The chromatin IP DNA Purification kit (ACTIVE-MOTIF, Carlsbad, CA, USA) was used for ChIP-qPCR in accordance with the manufacturer's instructions. Antibodies against TEAD4 or IgG were used as the control in ChIP analysis. After de-crosslinking the eluates, ChIP-enriched DNA was extracted and used for qPCR examination.

Plasmid transfection and luciferase reporter assays

The NRP1 promoter was added to the pGL3.0-basic vector to construct the luciferase reporter plasmid. The luciferase reporter was used to transfect HEK293T cells. The Dual-Luciferase Reporter Assay System (Promega, Madison, USA) was used to measure luciferase activity 48 hours post transfection following the manufacturer's instructions.

Histology and Immunohistochemistry

Samples of cancer tissue were taken, preserved in 4% paraformaldehyde, embedded in paraffin wax, and sectioned using a paraffin slicer into 5 mm sections for additional examination. The general morphology of the tissue was investigated with H&E staining kit (Solarbio, Beijing, China). Tissue protein expression was assessed in situ using an immunohistochemistry kit (MXB, Fuzhou, China). More specific procedures were described before (3).

Dataset analysis

The A549 radiation-resistant cell dataset (GSE1197236) from the Gene Expression Omnibus (GEO) database was used to select all differentially expressed genes (DEGs). The transcription factor and transcriptional coactivator datasets were downloaded from the AnimalTFDB4 website (guolab.wchscu.cn/AnimalTFDB4//#/). Genes associated with the Hippo signaling pathway were acquired from the Kyoto Encyclopedia of Genes and Genomes (KEGG) database (https://www.kegg.jp). Genes associated with poor prognosis in lung adenocarcinoma were determined using The Cancer Genome Atlas (TCGA) database (https://portal.gdc.cancer.gov/). In-house RNA sequencing was utilized to determine genes related with various genes coexisting in A549 and H1299 cells. In addition, collection of proteins interacting with YAP was collected from the STRING database (cn.string-db.org). The dataset of transcription factors binding to *NRP1* was obtained from JASPAR (https://jaspar.elixir.no).

Statistical analysis

The results are the representative of experiments repeated at least three times and were presented as mean ± SEM. Statistical analyses were conducted using SPSS software (version 24.0, SPSS Inc., Chicago, IL, USA). One-way ANOVA and a two-tailed Student's paired t-test were used to determine experimental validity. The n reflects the number of biologically independent experiments in each group. The results were highly significant at p-values < 0.05.

Supplementary Figures and Tables:

Figure S1. Establishment of radiation resistance models and the entry of YAP into the cell nucleus. Figure S2. *YAP* knockdown/overexpression inhibits/promotes the proliferation, migration, and efficiency of NSCLC cells. Figure S3. *NRP1* knockdown/overexpression inhibits/promotes the proliferation, migration, and efficiency of NSCLC cells, thereby affecting radiation resistance. Figure S4. TEAD4 is unable to regulate NRP1 expression. Figure S5. Rescue test results for *YAP* overexpression and *NRP1* suppression. Table S1. qRT-PCR and CHIP-qPCR primer sequences. Table S2. A list of all utilized antibodies and their dilutions. Table S3. sh-RNA sequences. Table S4. Quantitative results for colonies in rescue experiments.

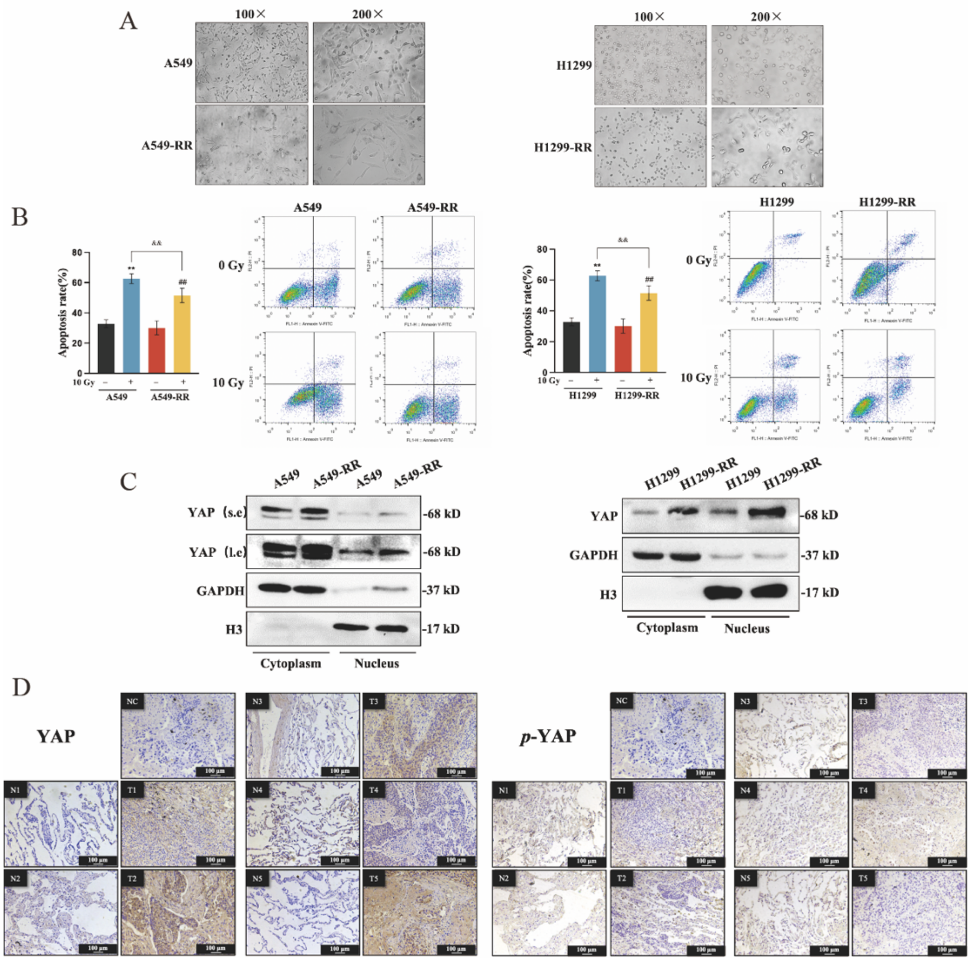

Fig. S1. Establishment of radiation resistance models and the entry of YAP into the cell nucleus. (A) Raw microscopy images of A549 and H1299 cell lines. (B) Apoptosis flow cytometry analysis. (C) Nuclear and cytoplasmic proteins levels of YAP. (D) IHC shows YAP and *p*-YAP protein levels in tumor tissue of five patients (100 μm, n=5). Mean ± SD, n = 3. ***p* < 0.01 *vs.* A549, ##*p* < 0.01 *vs.* H1299, and &&*p* < 0.01 *vs.* IR.

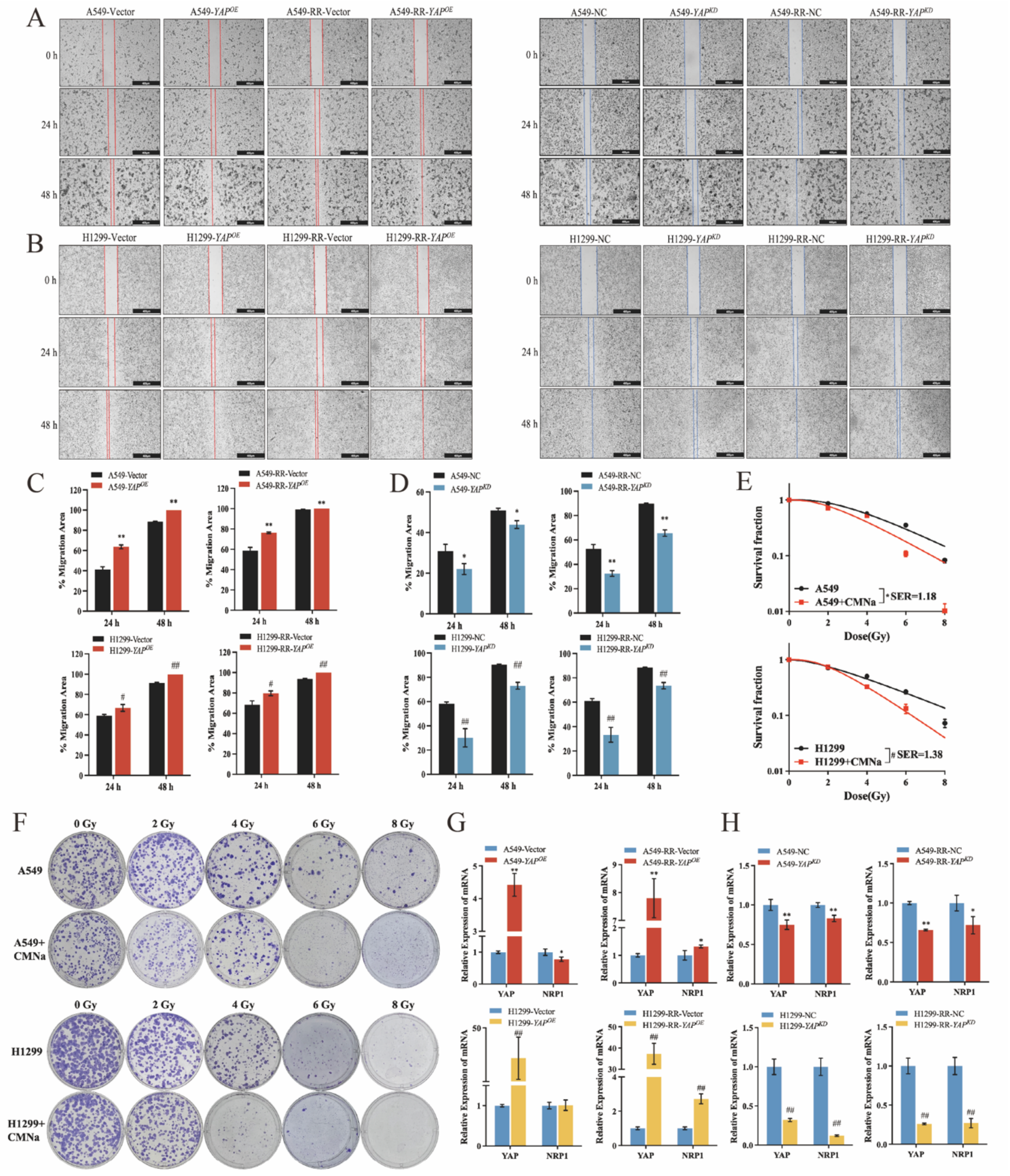

Fig. S2. ***YAP* knockdown/overexpression inhibits/promotes the proliferation, migration, and efficiency of NSCLC cells.** (A, B) Representative images and (C, D) quantitative results for the wound healing assay (400 μm). (F) Representative images and (E) quantitative results for the colony formation assay after adding CMNa (1mM). (G, H) *YAP* and *NRP1* mRNA levels. Mean ± SD, n = 3. $^{**}p < 0.01$ *vs.* Vector/NC, $^{*}p < 0.05$ *vs.* Vector/NC, $^{\#\#}p < 0.01$ *vs.* Vector/NC, and $^{\#}p < 0.05$ *vs*. Vector/NC.

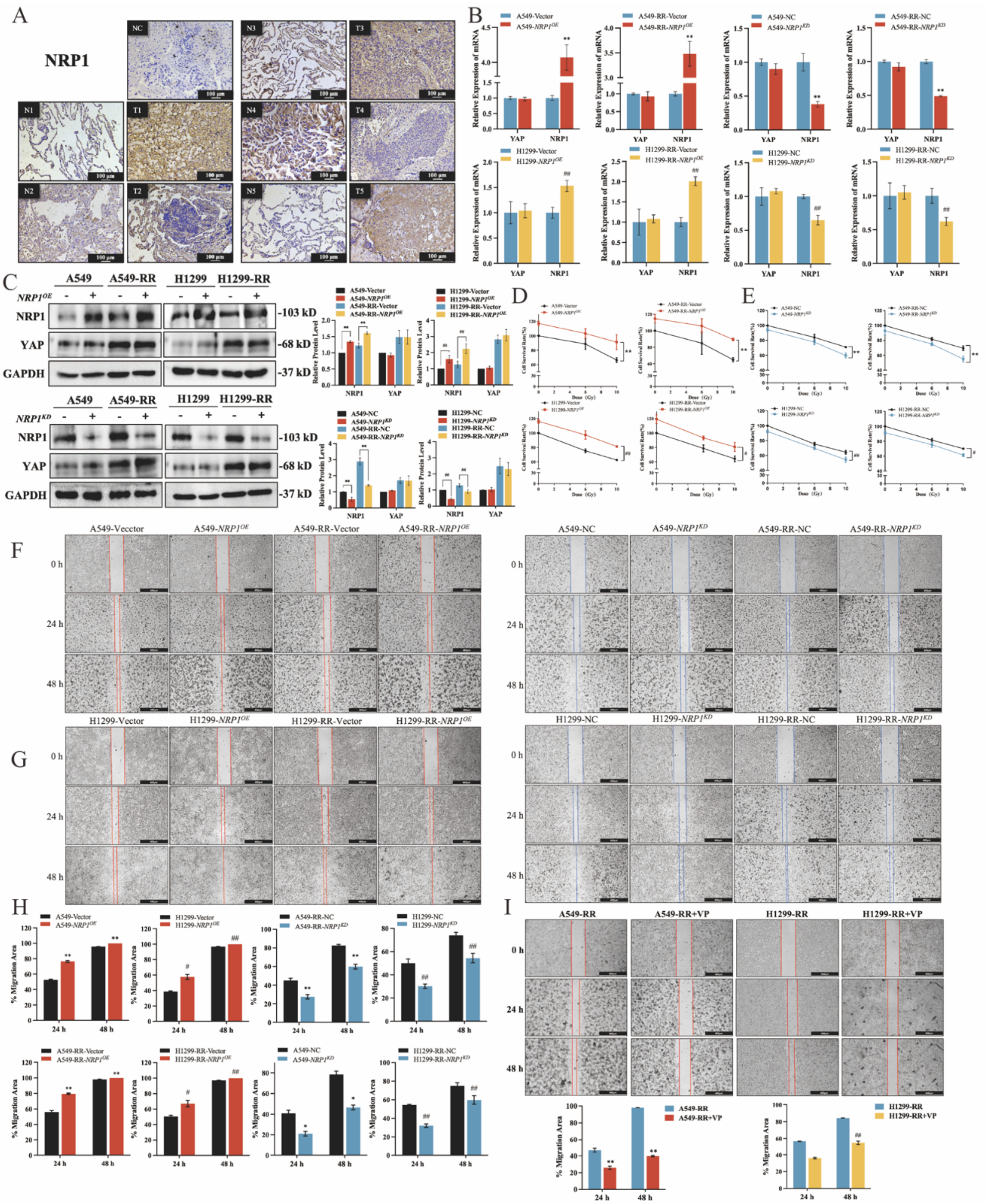

Fig. S3. *NRP1* knockdown/overexpression inhibits/promotes the proliferation, migration, and efficiency of NSCLC cells, thereby affecting radiation resistance. (A) IHC results showing NRP1 protein levels in tumor tissue of five patients (100 μm). YAP and NRP1 (B) mRNA and (C) protein levels. (D, E) CCK-8 assay was performed to determine cell viability and proliferation. (F, G) Representative images and (H) quantitative results for the wound healing assay (400 μm). (I) Representative images for wound healing assay after VP treatment (400 μm).

Mean ± SD, n = 3. **$p < 0.01$ *vs.* Vector/NC, *$p < 0.05$ *vs.* Vector/NC, ##$p < 0.01$ *vs.* Vector/NC, and #$p < 0.05$ *vs.* Vector/NC.

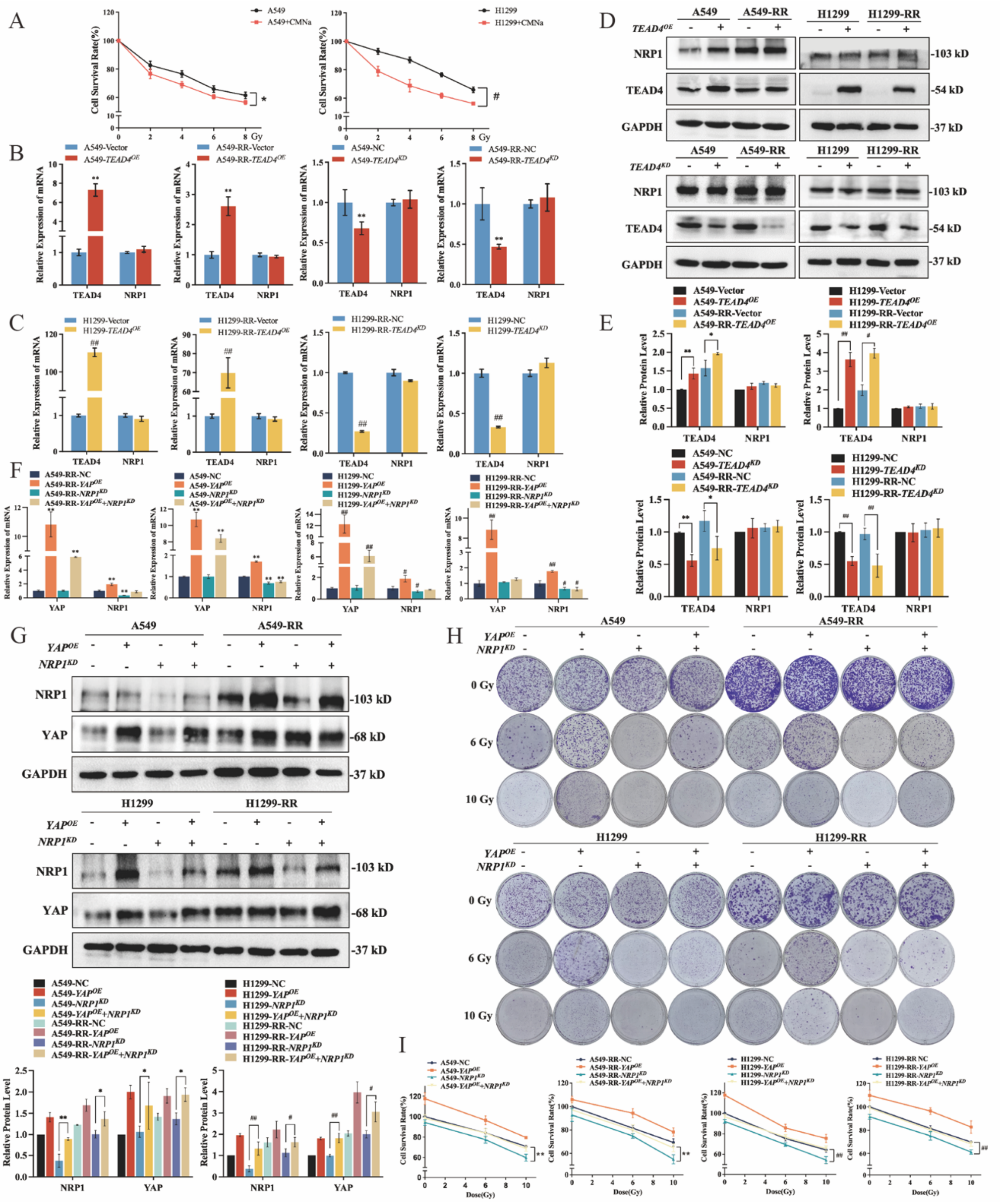

Fig. S4. TEAD4 is unable to regulate NRP1 expression. (A) CCK-8 assay was performed to determine cell viability and proliferation after CMNa treatment (1 mM). (B, C) *TEAD4* and *NRP1* mRNA levels after *TEAD4* silencing or overexpression. (D, E) TEAD4 and NRP1 protein levels after *TEAD4* silencing or overexpression. (F) mRNA levels after *NRP1* silencing or *YAP*

overexpression. (G) Protein levels after *NRP1* silencing or *YAP* overexpression. (H) Representative images for the colony formation assay after *NRP1* silencing or *YAP* overexpression. (I) CCK-8 assay was performed after *NRP1* silencing or *YAP* overexpression. Mean ± SD, n = 3. $^{**}p < 0.01$ *vs.* Vector/NC, $^{*}p < 0.05$ *vs.* Vector/NC, $^{\#\#}p < 0.01$ *vs.* Vector/NC, and $^{\#}p < 0.05$ *vs.* Vector/NC.

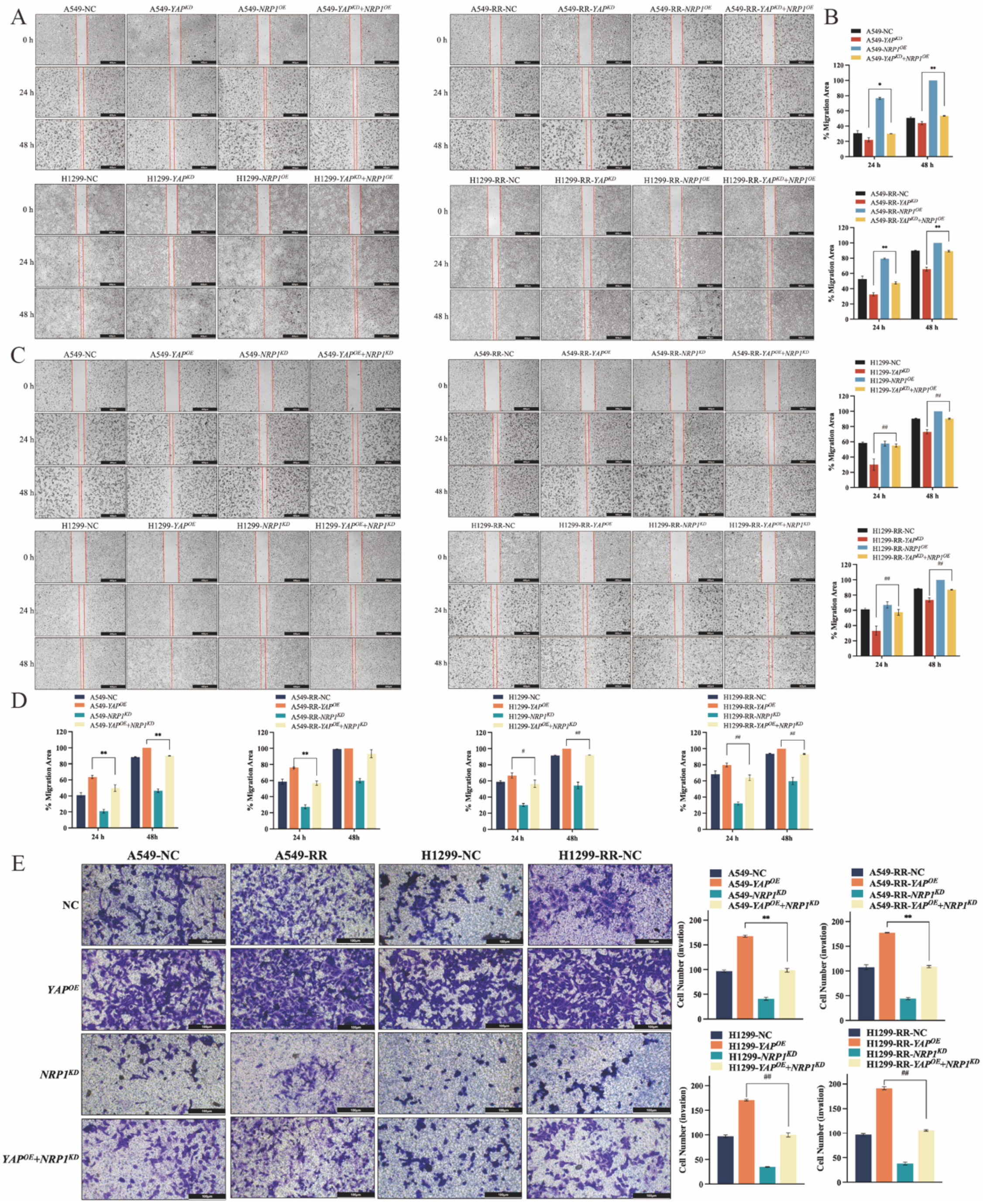

Fig. S5. Rescue test results for *YAP* overexpression and NRP1 suppression. (A, C) Representative images and (B, D) quantitative results for wound healing assay after *NRP1* silencing or *YAP* overexpression (400 μm). (E) Transwell assay after *NRP1* silencing or *YAP* overexpression. Mean ± SD, n = 3. **p < 0.01 vs. NC, *p < 0.05 vs. NC, ##p < 0.01 vs. NC, and #p < 0.05 vs. NC.

Table S1. qRT-PCR and ChIP-qPCR primer sequences.

Primers		Sequence 5' to 3'
h-GAPDH	Forward	ACGGATTTGGTCGTATTGGG
	Reverse	TGATTTTGGAGGGATCTCGC
h-YAP	Forward	GCTCTAGAATGGATCCCGGGCAGCAGCC
	Reverse	CGGAATTCTAACCATGTAAGAAAGCTTT
h-NRP1	Forward	CCCCAAACCACTGATAACTCG
	Reverse	AGACACCATACCCAACATTCC
h-TEAD4	Forward	GAACGGGGACCCTCCAATG
	Reverse	GCGAGCATACTCTGTCTCAAC
CHIP-qPCR-h-NRP1-promoter	Forward	CACACTCAGCAGGGAAAGG
	Reverse	GAGCGCCCGTTTGGATAG

Table S2. A list of all utilized antibodies and their dilutions.

Antibody	Company	Cat.	Dilutions
YAP	CST	D8H1X	1:1000
NRP1	Abcam	Ab81321	1:1000
GAPDH	OriGene	TA802519	1:1000
p-YAP	CST	D9W2I	1:1000
TEAD4	Abcam	Ab197589	1:1000
Histone-H3	Abcam	Ab1791	1:1000
Goat anti-Mouse IgG (H+L) HRP	BS12478	Bioworld	1:50000
Goat anti-Rabbit IgG (H+L) HRP	BS13278	Bioworld	1:10000
Cy3 goat antibody	ABclonal	AS007	1:250
Normal Rabbit IgG	2729S	CST	1:100

Table S3. sh-RNA sequences.

Plasmids	Sequence
NC	GTTCTCCGAACGTGTCACGTT
YAP^{KD}	GCCACCAAGCTAGATAAAGAA
NRP1^{KD}	GCCAGAGGAGTACGATCAGTT
TEAD4^{KD}	GCCCAGTTAAATGTTCACCAAT

Table S4. Quantitative results for colonies in rescue experiments.

Group	D_0 (Gy)	N (Gy)	SER
A549-YAP^{KD}	1.571	16.62	0.43^{**}
A549-YAP^{KD}+$NRP1^{OE}$	3.676	2.51	
A549-RR-YAP^{KD}	1.049	85.45	0.53^{**}
A549-RR-YAP^{KD}+$NRP1^{OE}$	1.992	15.26	
H1299-YAP^{KD}	1.066	72.8	$0.27^{\#\#}$
H1299-YAP^{KD}+$NRP1^{OE}$	3.883	2.288	
H1299-RR-YAP^{KD}	1.945	6.148	$0.47^{\#\#}$
H1299-RR-YAP^{KD}+$NRP1^{OE}$	4.106	3.197	
A549-YAP^{OE}	3.445	4.057	2.04^{**}
A549-YAP^{OE}+$NRP1^{KD}$	1.688	12.87	
A549-RR-YAP^{OE}	3.545	2.562	1.95^{**}
A549-RR-YAP^{OE}+$NRP1^{KD}$	1.821	7.689	
H1299-YAP^{OE}	4.341	1.954	$3.58^{\#\#}$
H1299-YAP^{OE}+$NRP1^{KD}$	1.211	36.28	
H1299-RR-YAP^{OE}	3.611	4.065	$2.95^{\#\#}$
H1299-RR-YAP^{OE}+$NRP1^{KD}$	1.223	37.31	

SI References

1. Cong L, Yi J, Qiu S, Wang R, Jin S, Jiang R, et al. Effect of EG00229 on Radiation Resistance of Lung Adenocarcinoma Cells. J Cancer. 2021;12(20):6105-17.
2. Yi J, Gao H, Wei X, Wang M, Xu W, Yu D, et al. The transcription factor GATA3 positively regulates NRP1 to promote radiation-induced pulmonary fibrosis. Int J Biol Macromol. 2024;262(Pt 2):130052.
3. Wei W, Zhang HY, Gong XK, Dong Z, Chen ZY, Wang R, et al. Mechanism of MEN1 gene in radiation-induced pulmonary fibrosis in mice. Gene. 2018;678:252-60.
