# Supplementary material for: Radiation-induced YAP/TEAD4 binding confers non-small cell lung cancer radioresistance via promoting NRP1 transcription
Source: Cell Death Dis. 2024 Aug 26;15(8):619. doi: 10.1038/s41419-024-07017-6 (PMC11347582; doi:10.1038/s41419-024-07017-6)
Supplement: Supplementary file 2 — Original Western blots Data [file 41419_2024_7017_MOESM2_ESM.pdf]

Original Western blots Data

Fig1. G+ +Fig3. C

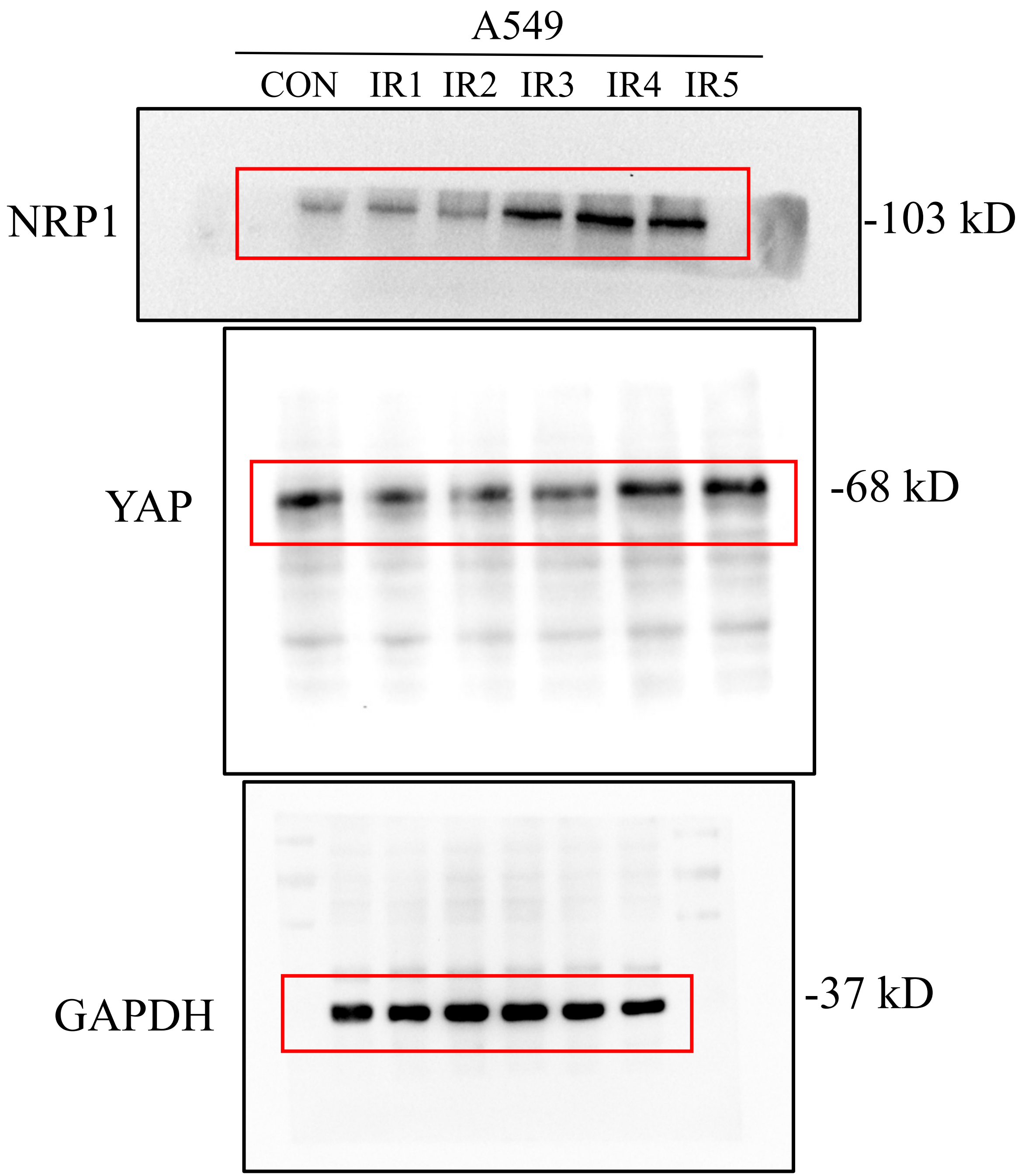
A549
CON IR1 IR2 IR3 IR4 IR5
NRP1
-103 kD
YAP
-68 kD
GAPDH
-37 kD

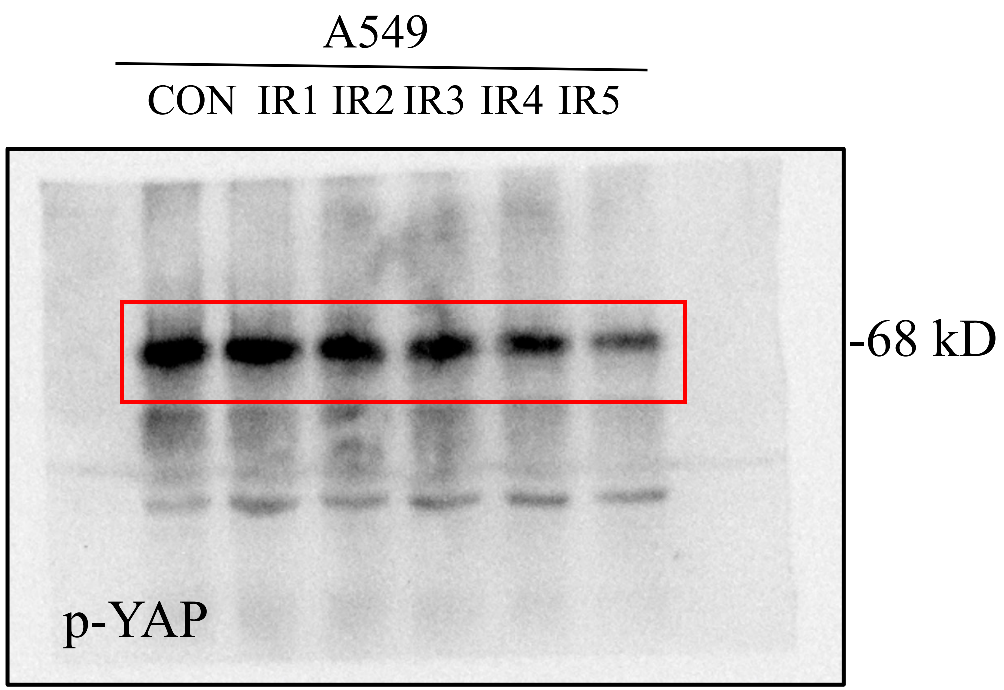
A549
CON IR1 IR2 IR3 IR4 IR5
-68 kD
p-YAP

Fig1. G+ +Fig3. C-2

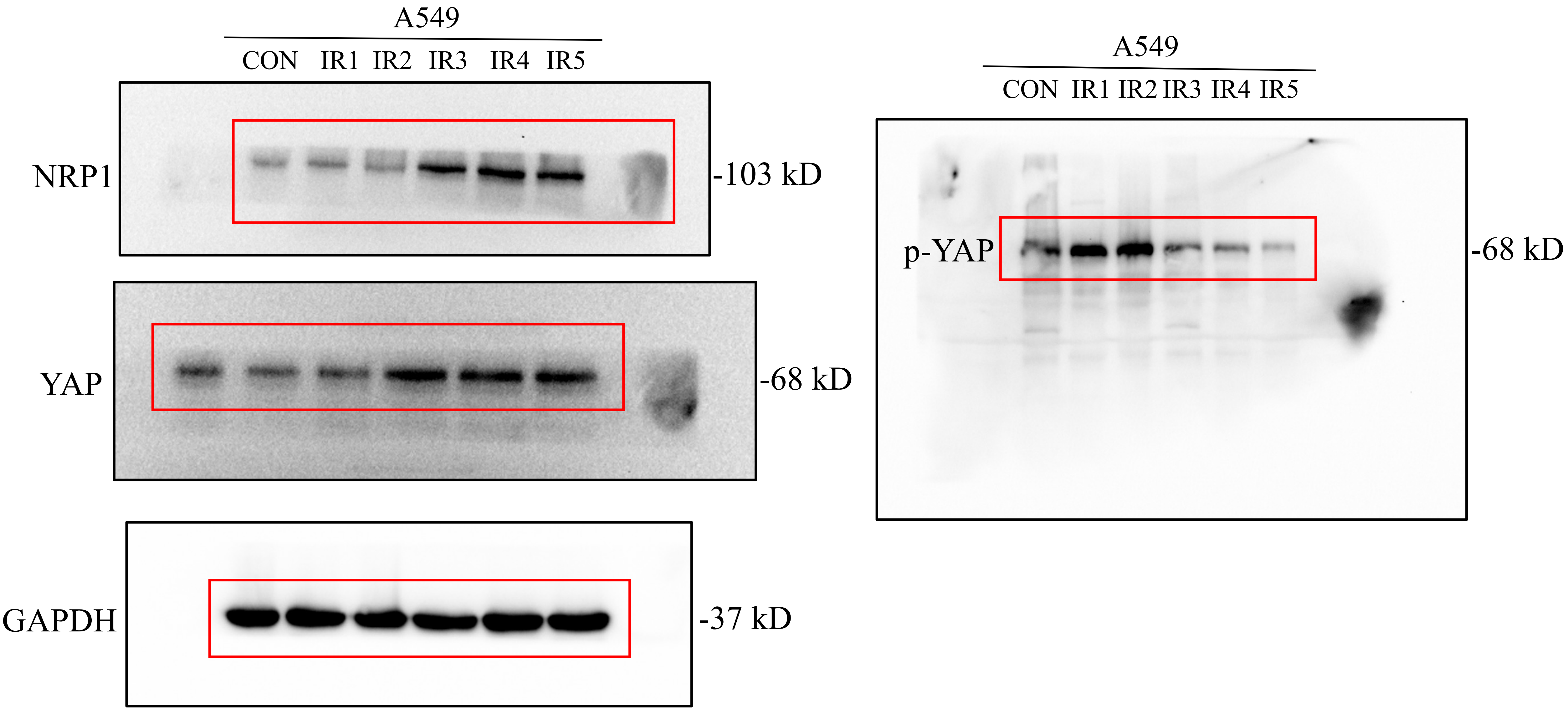

Fig1. G+ +Fig3. C-3

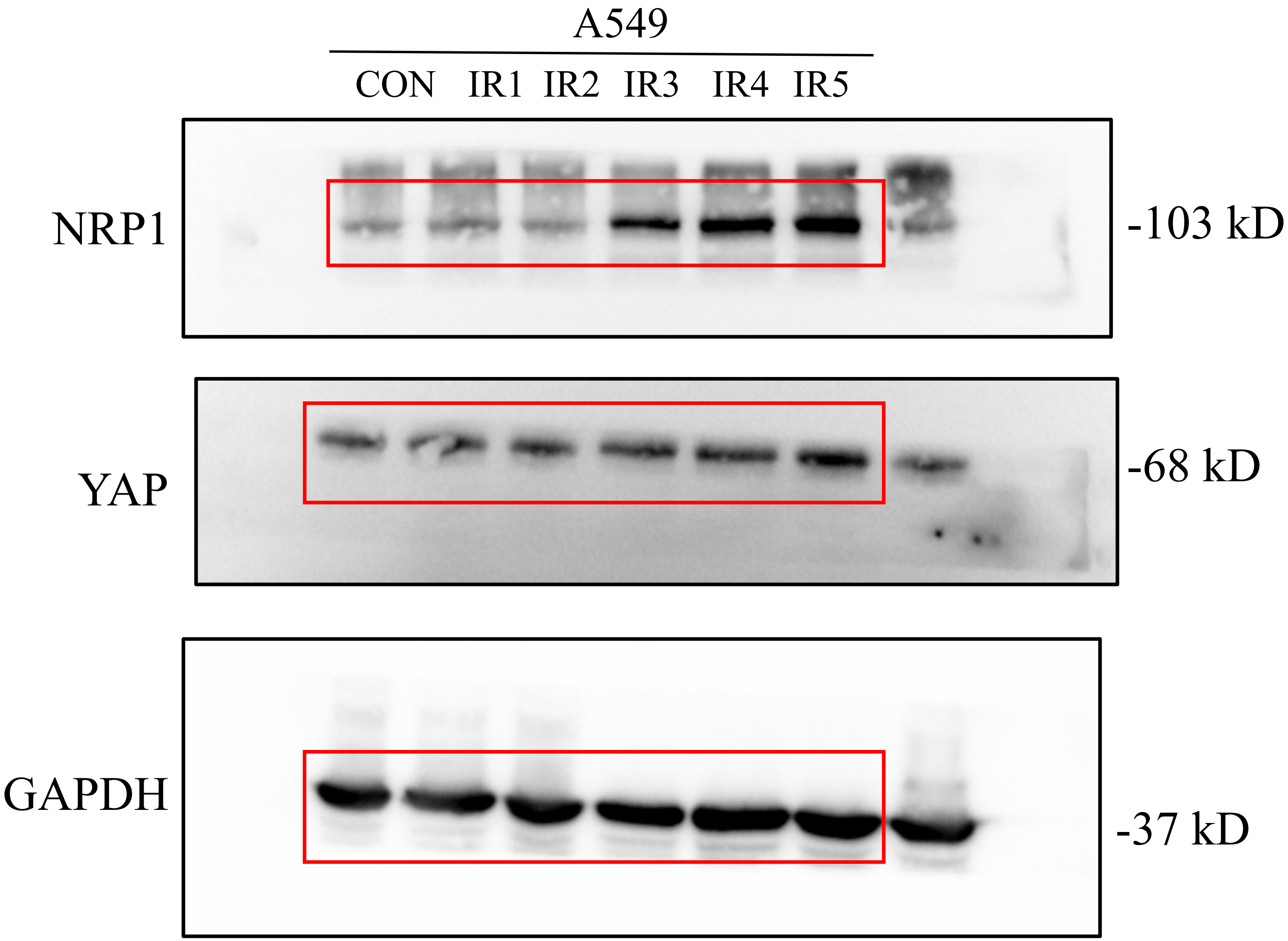

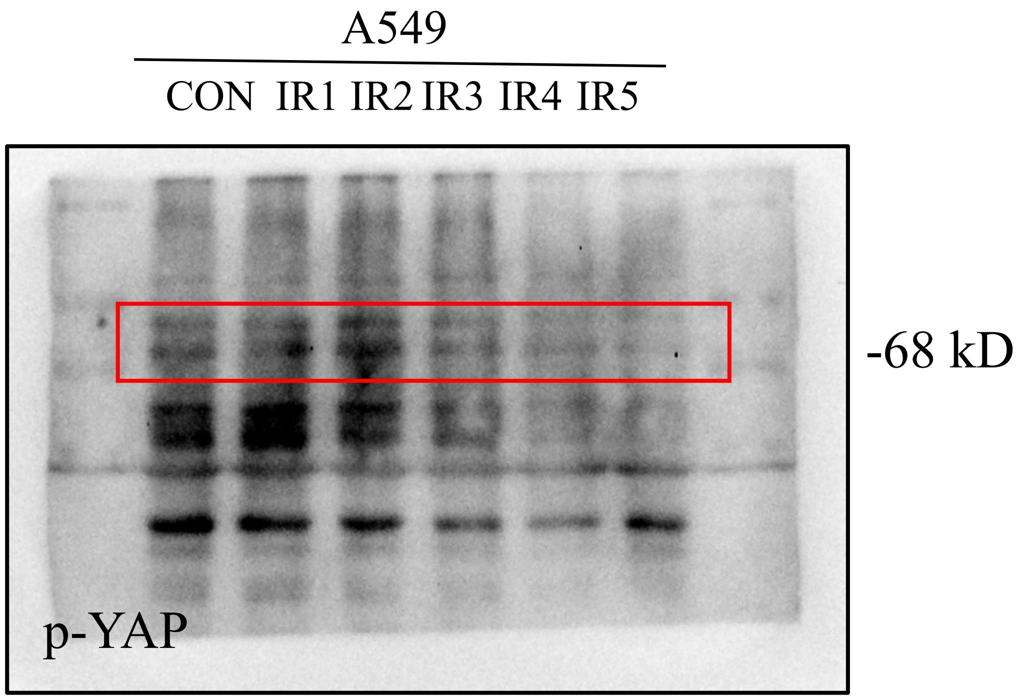

Fig1. G+Fig3. C

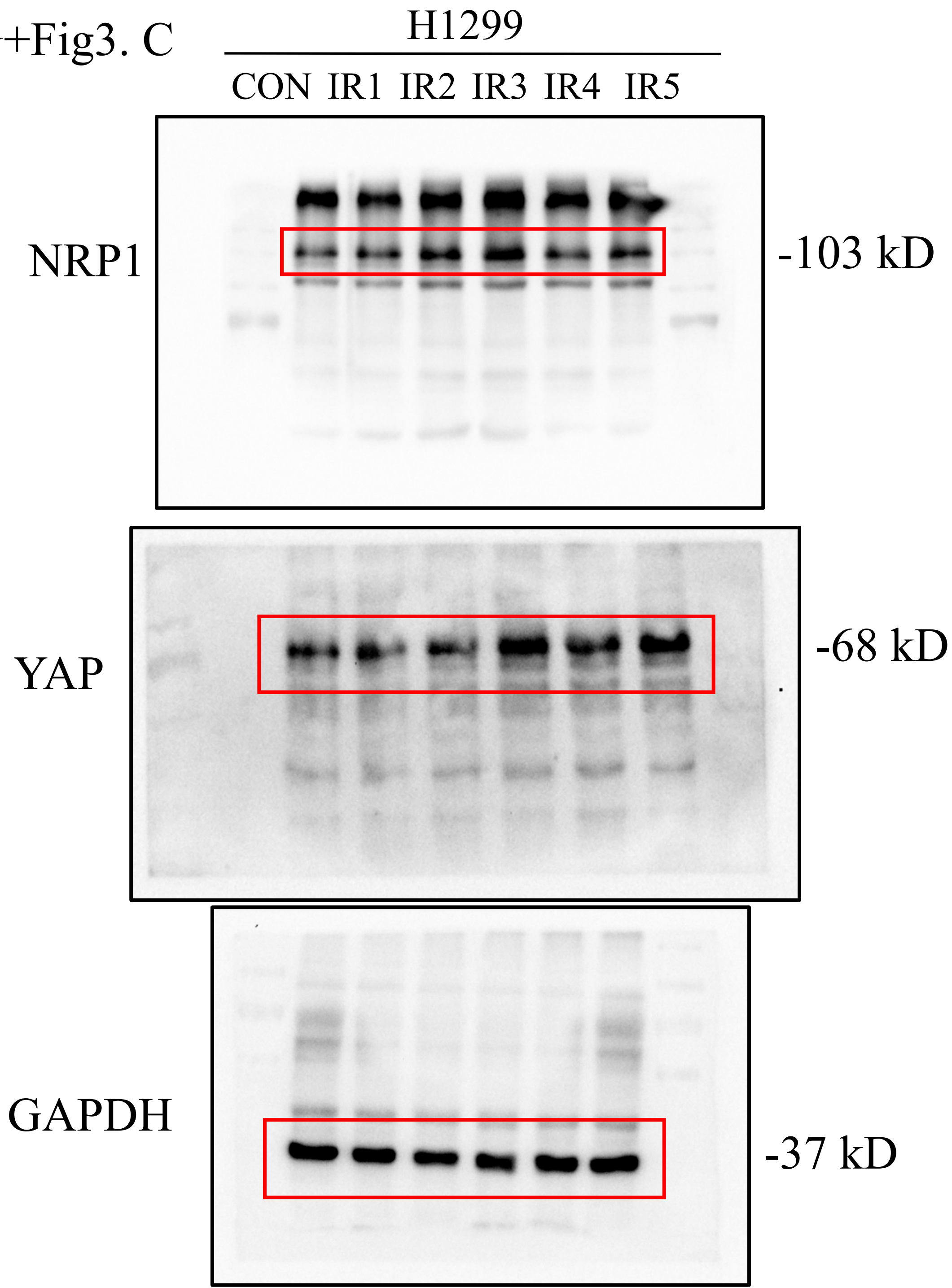
H1299
CON IR1 IR2 IR3 IR4 IR5
NRP1
-103 kD
YAP
-68 kD
GAPDH
-37 kD

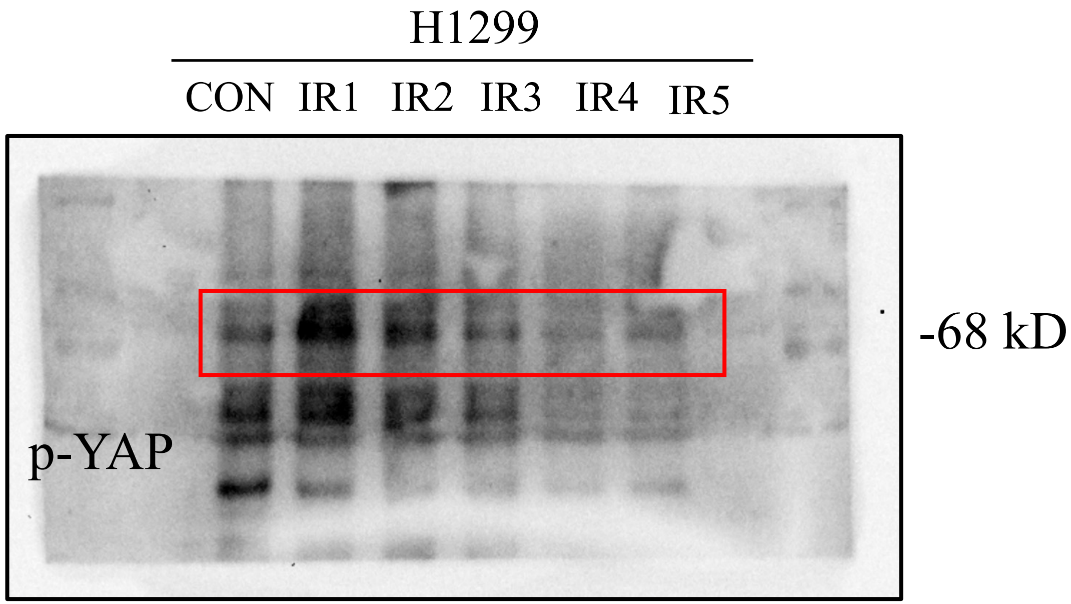
H1299
CON IR1 IR2 IR3 IR4 IR5
p-YAP
-68 kD

Fig1. G+Fig3. C-2

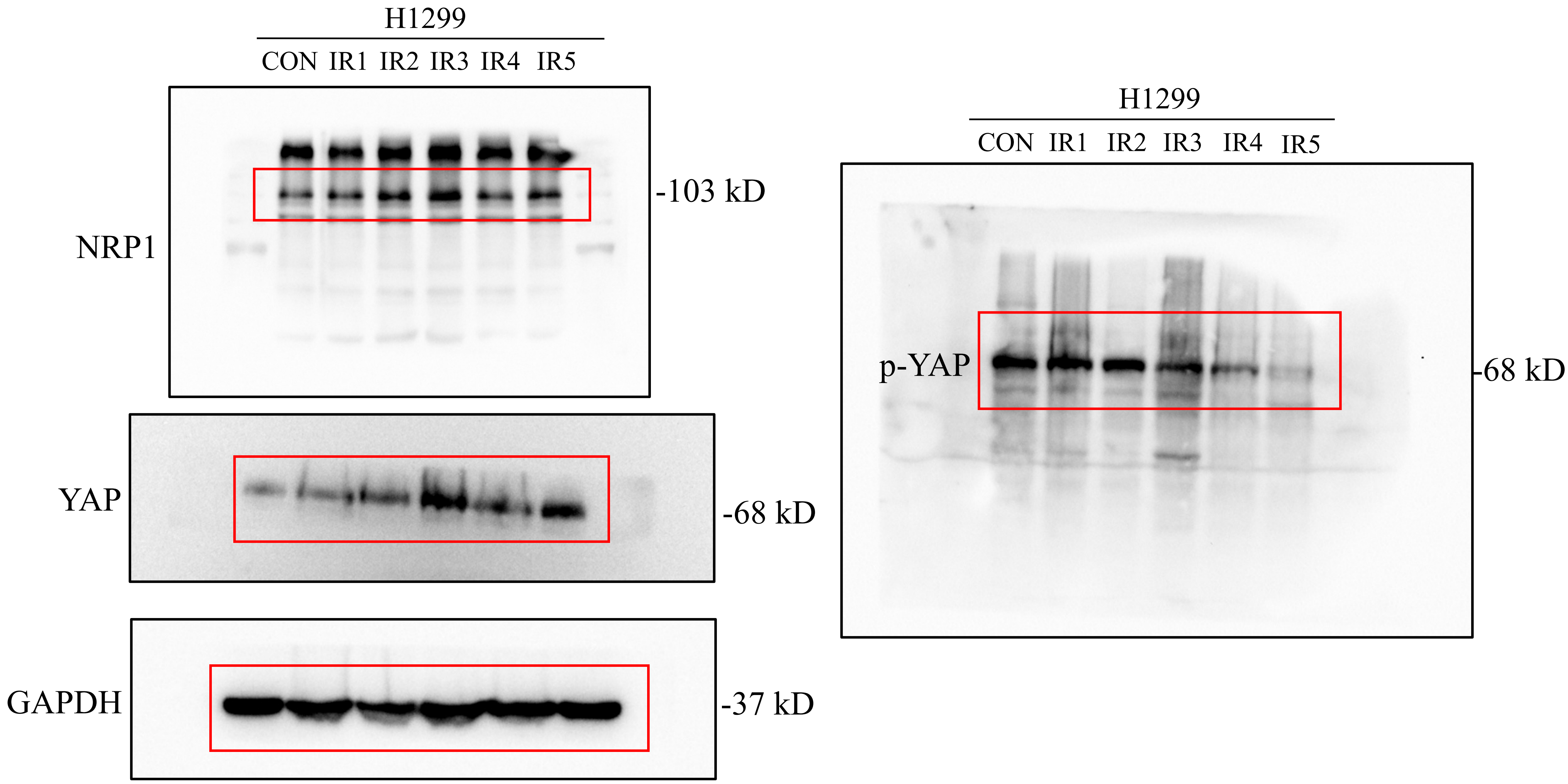

Fig1. G+Fig3. C-3

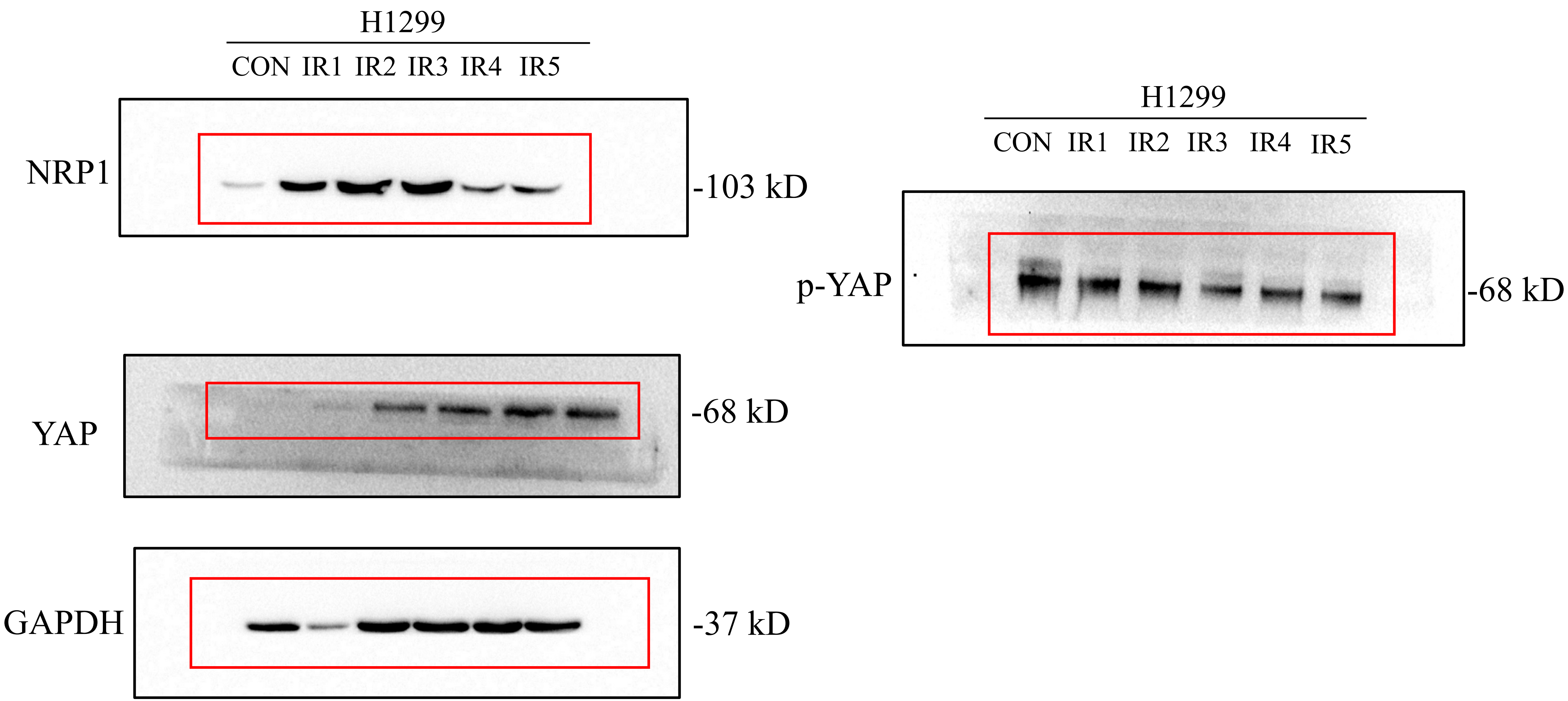

Fig3. F

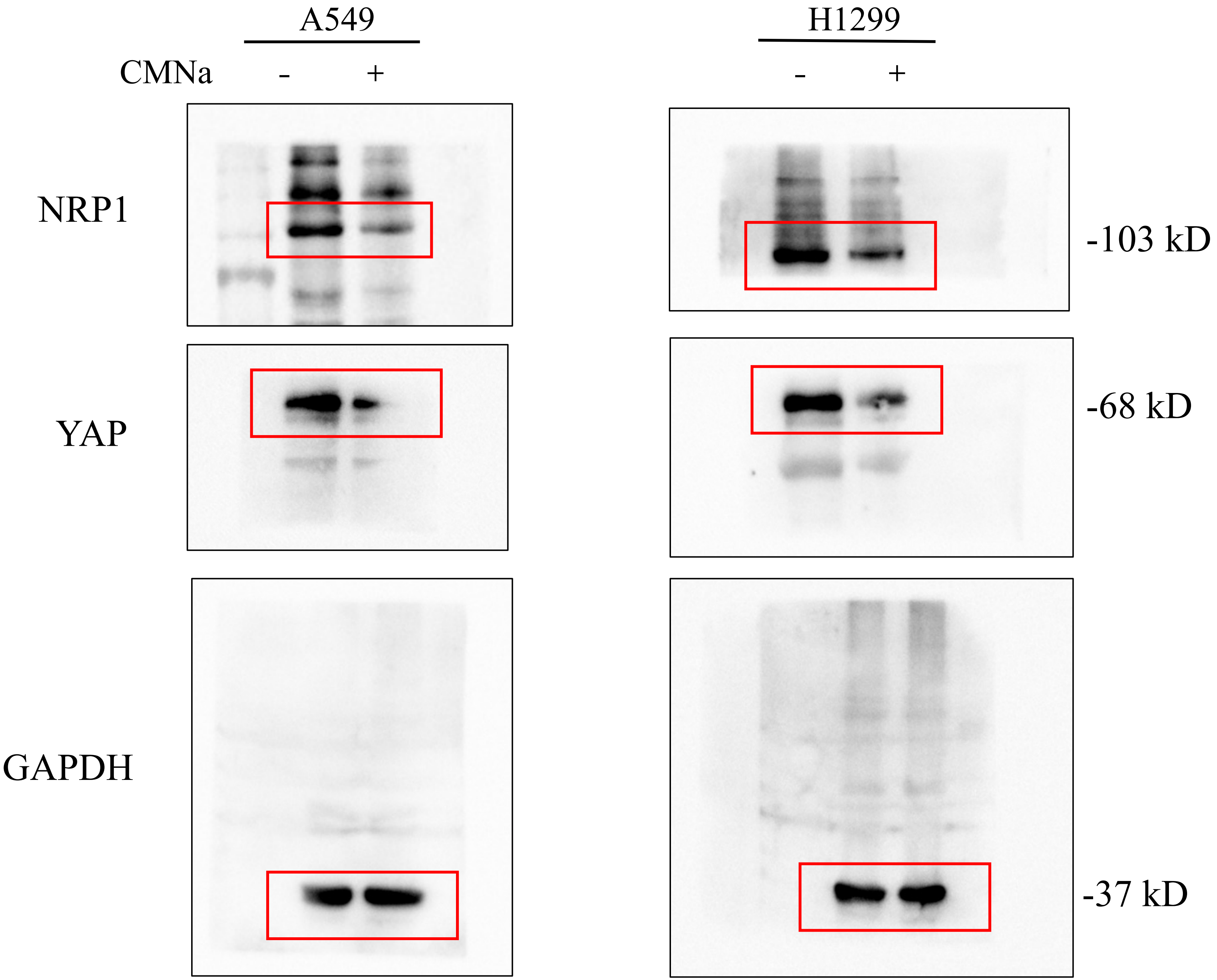

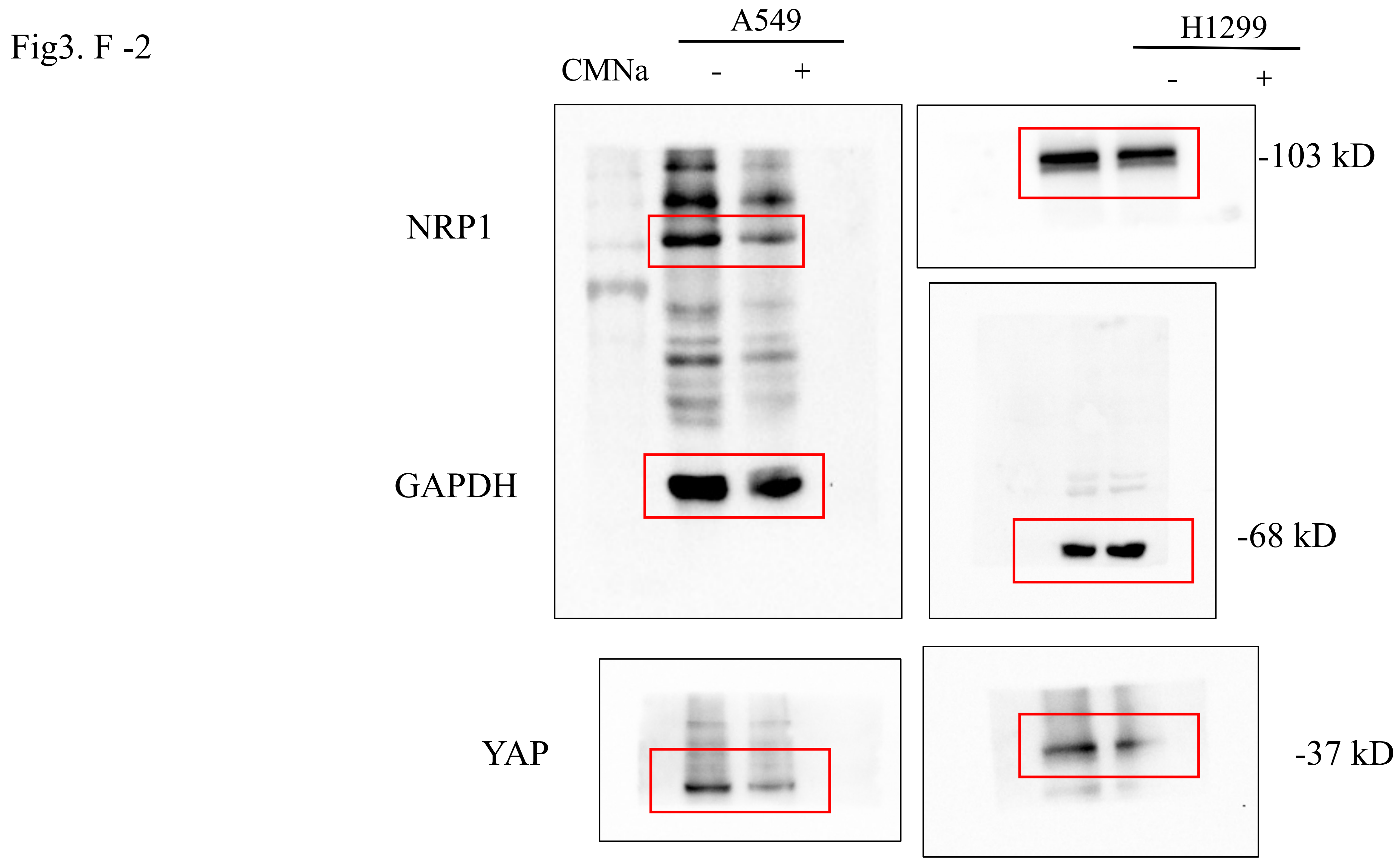
Fig3. F -2
A549
H1299
CMNa
-
+
-
+
NRP1
GAPDH
YAP
-103 kD
-68 kD
-37 kD

Fig3. F -3

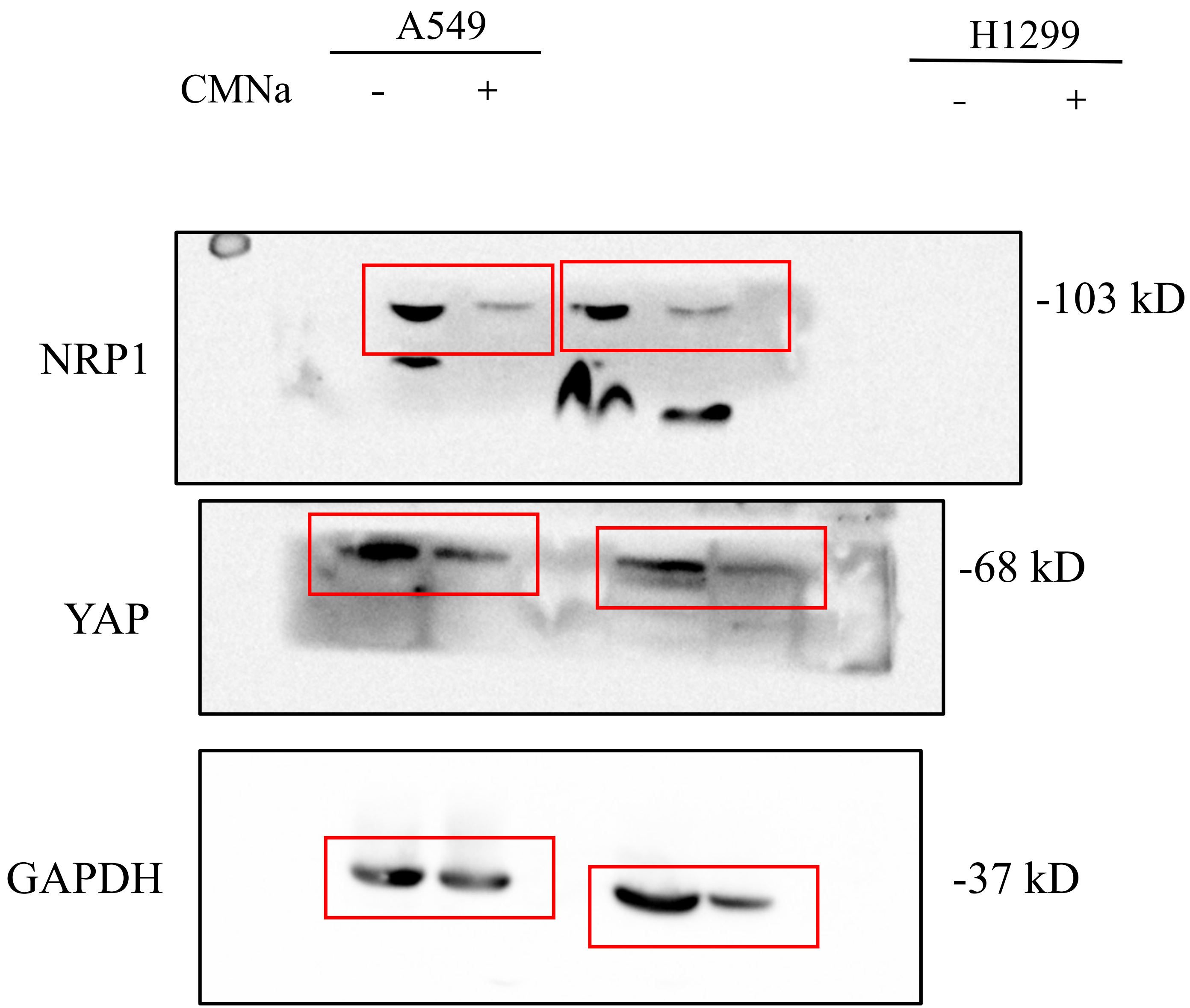

Fig3.G

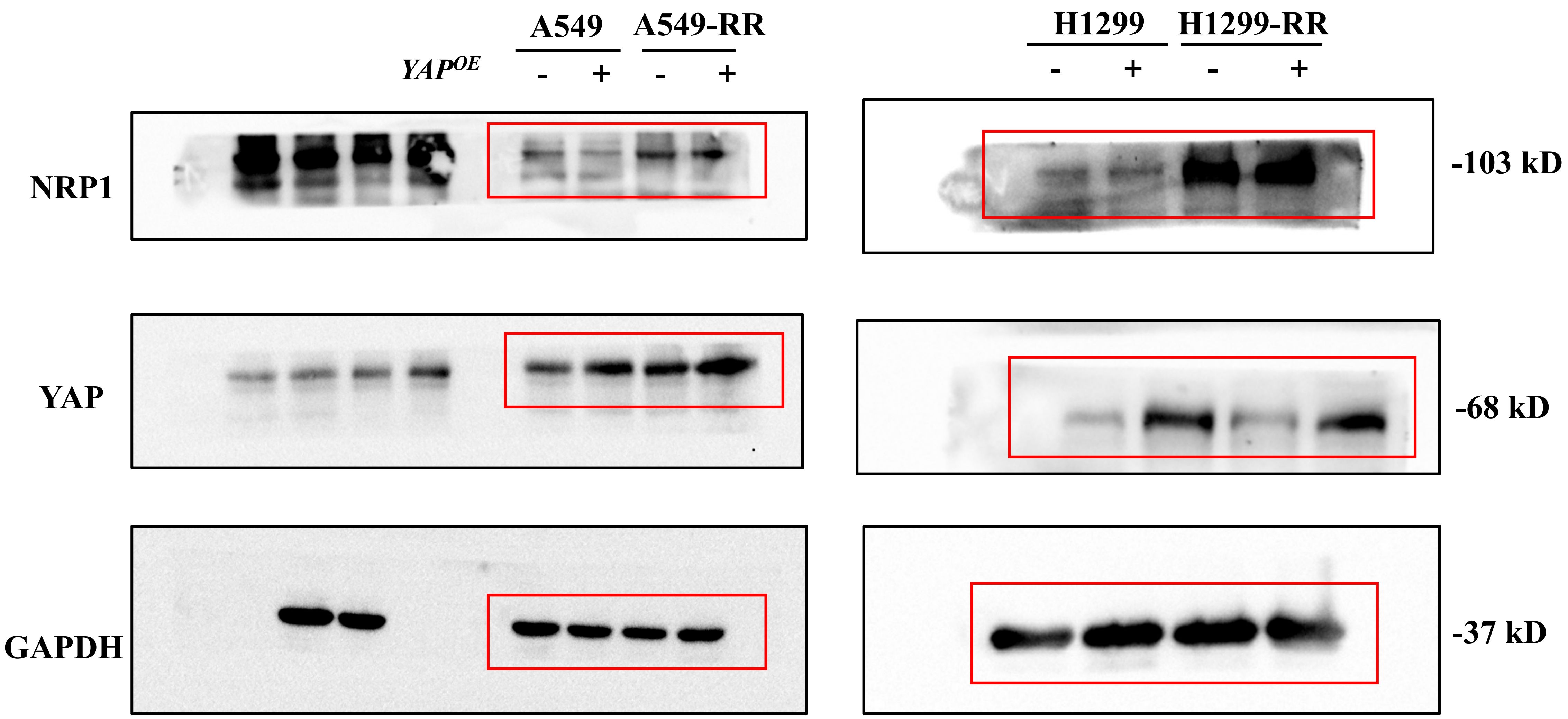
A549
A549-RR
H1299
H1299-RR
YAP^OE
- + - +
- + - +
NRP1
YAP
GAPDH
-103 kD
-68 kD
-37 kD

Fig3.G-2

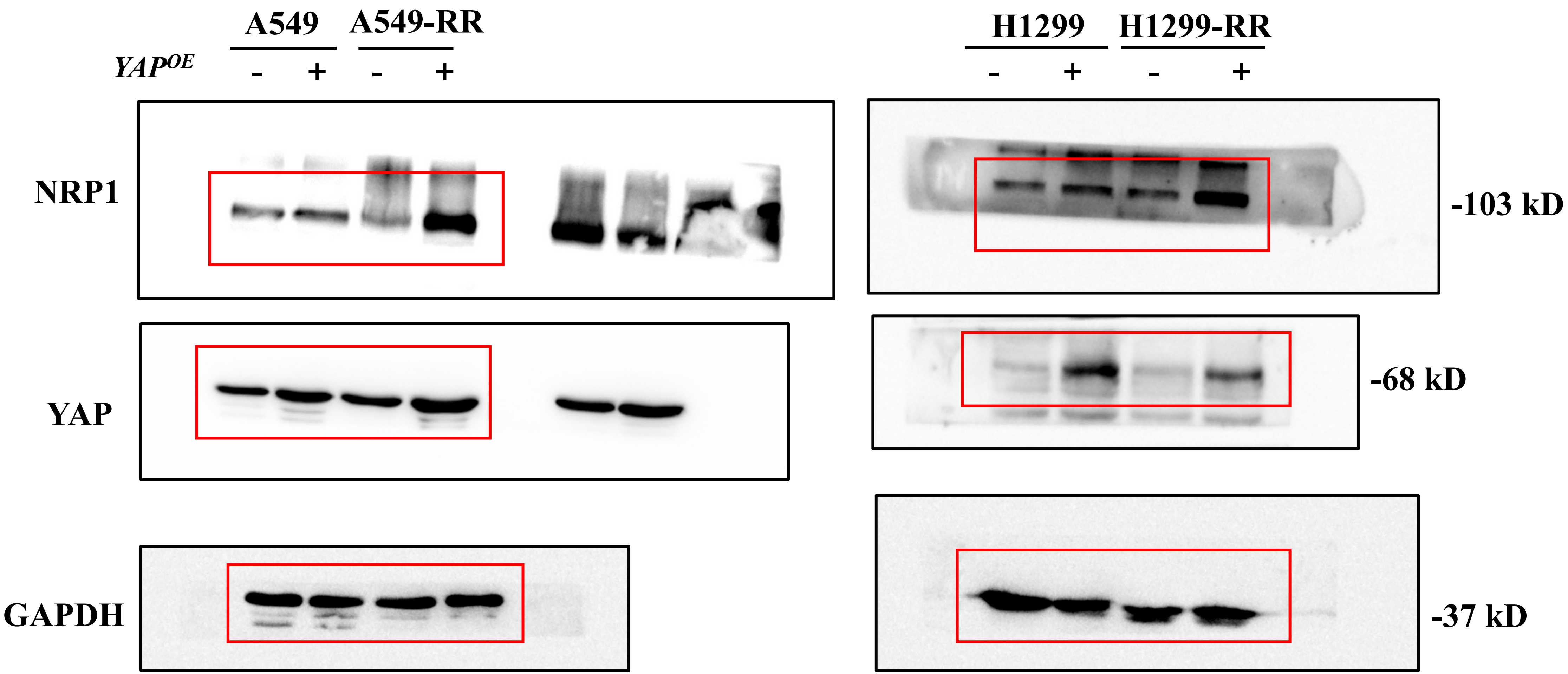
A549
A549-RR
H1299
H1299-RR
YAP^OE
-
+
-
+
NRP1
YAP
GAPDH
-103 kD
-68 kD
-37 kD

Fig3.G-3

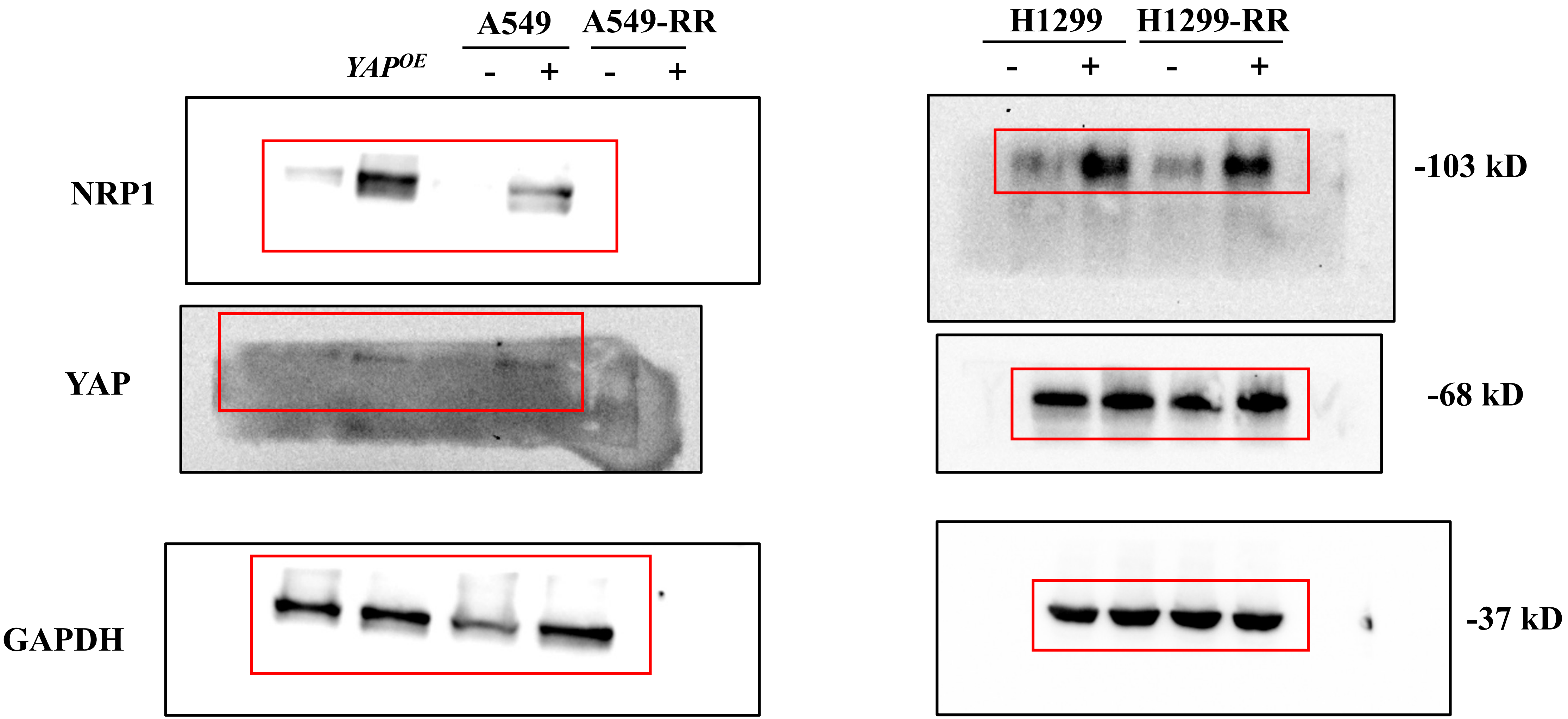
A549
A549-RR
YAP^OE
- + - +
H1299
H1299-RR
- + - +
NRP1
YAP
GAPDH
-103 kD
-68 kD
-37 kD

Fig3.G

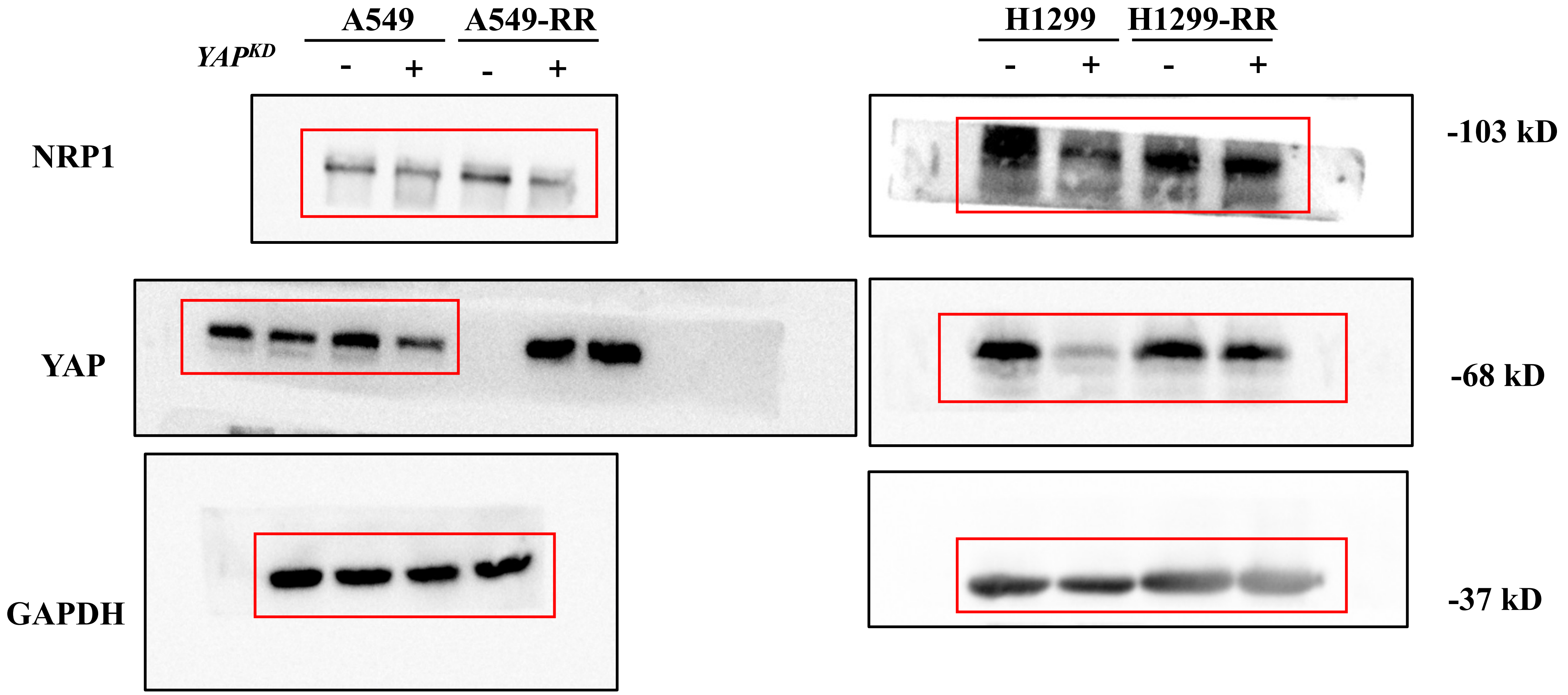

A549
A549-RR
H1299
H1299-RR
YAP^{KD}
-
+
-
+
-
+
-
+
NRP1
YAP
GAPDH
-103 kD
-68 kD
-37 kD

Fig3.G-2

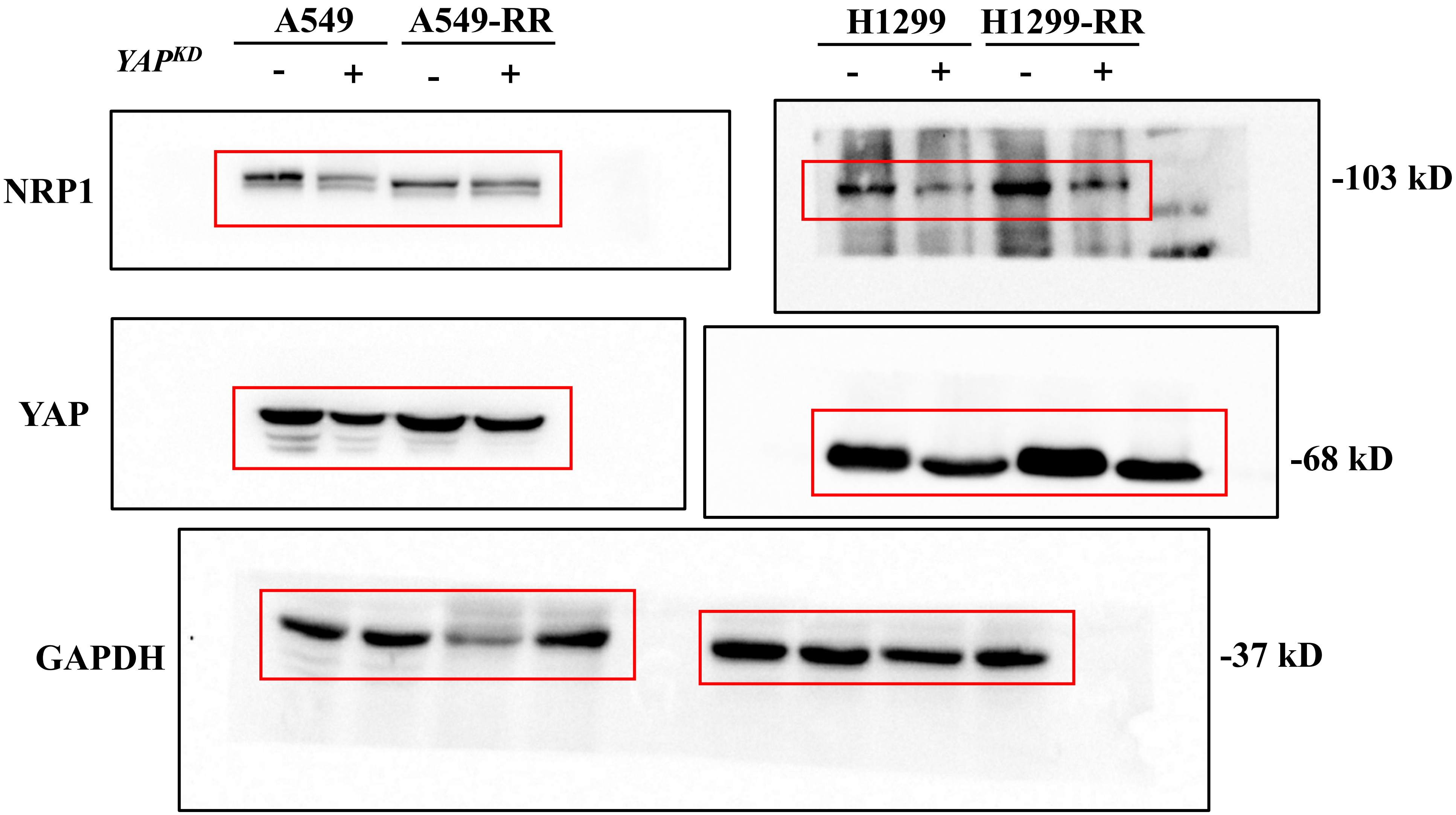
A549
A549-RR
H1299
H1299-RR
YAP^KD
- + - +
- + - +
NRP1
YAP
GAPDH
-103 kD
-68 kD
-37 kD

Fig3.G-3

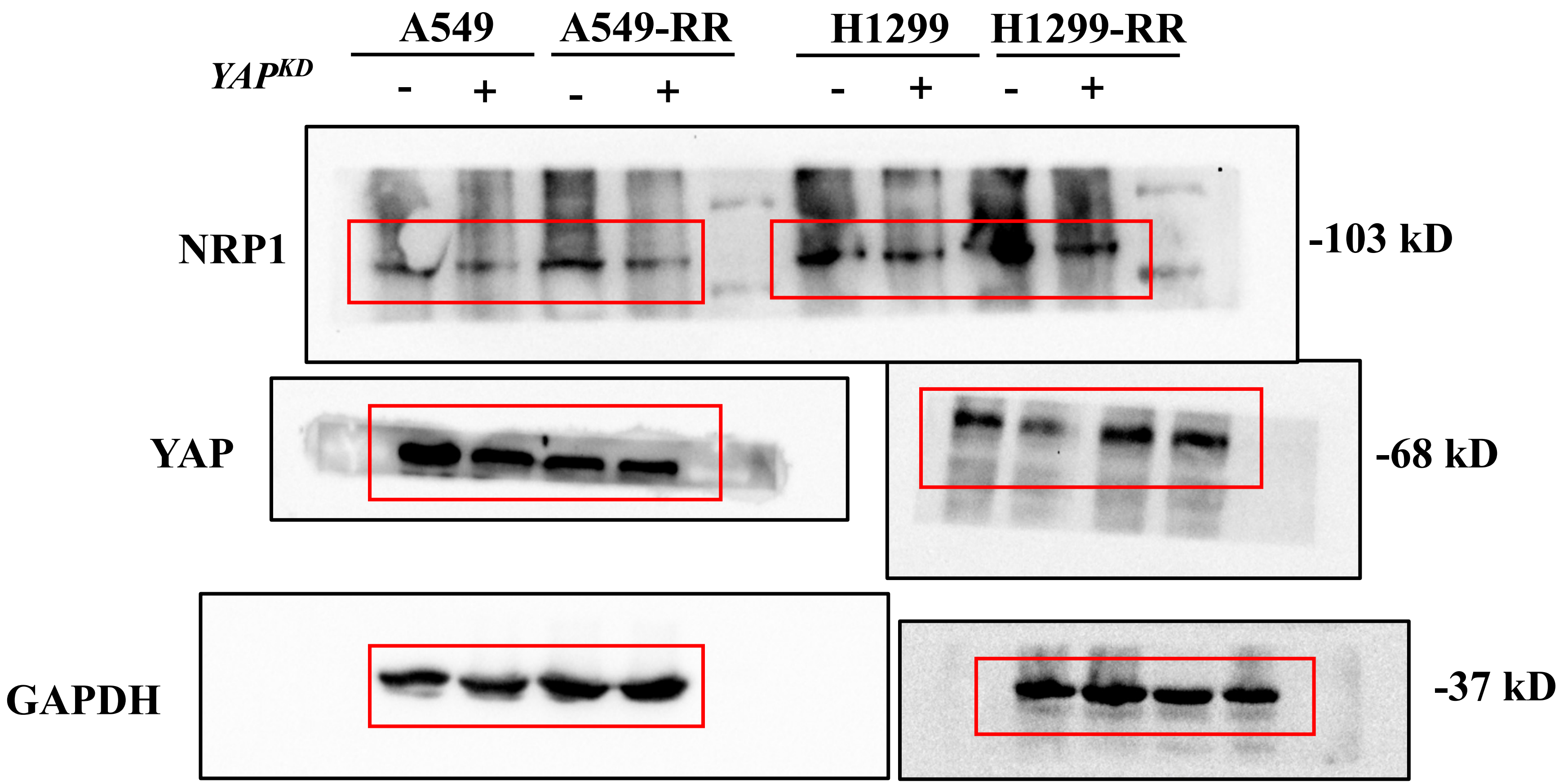
A549
A549-RR
H1299
H1299-RR
YAPKD
-
+
-
+
-
+
-
+
NRP1
YAP
GAPDH
-103 kD
-68 kD
-37 kD

Fig4.D

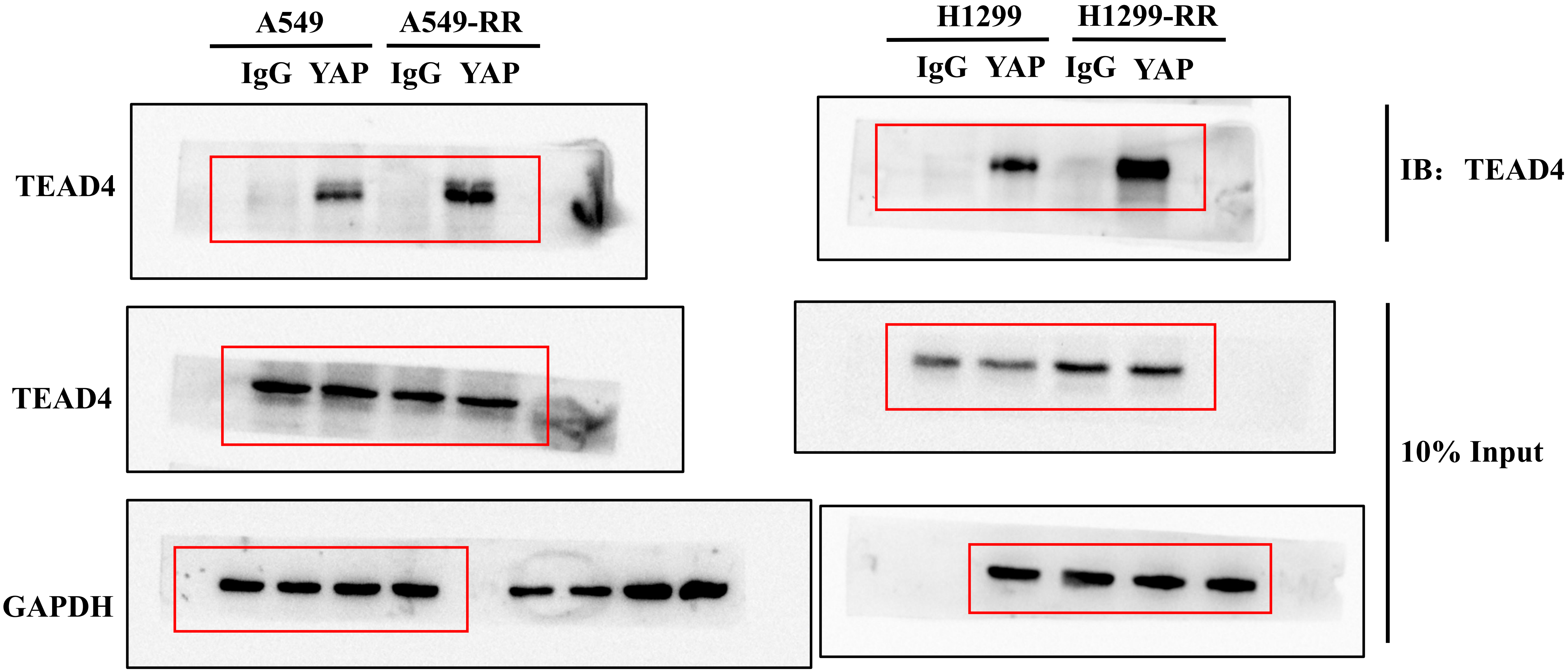

A549
A549-RR
IgG YAP IgG YAP
H1299
H1299-RR
IgG YAP IgG YAP
TEAD4
IB: TEAD4
TEAD4
GAPDH
10% Input

Fig4.D

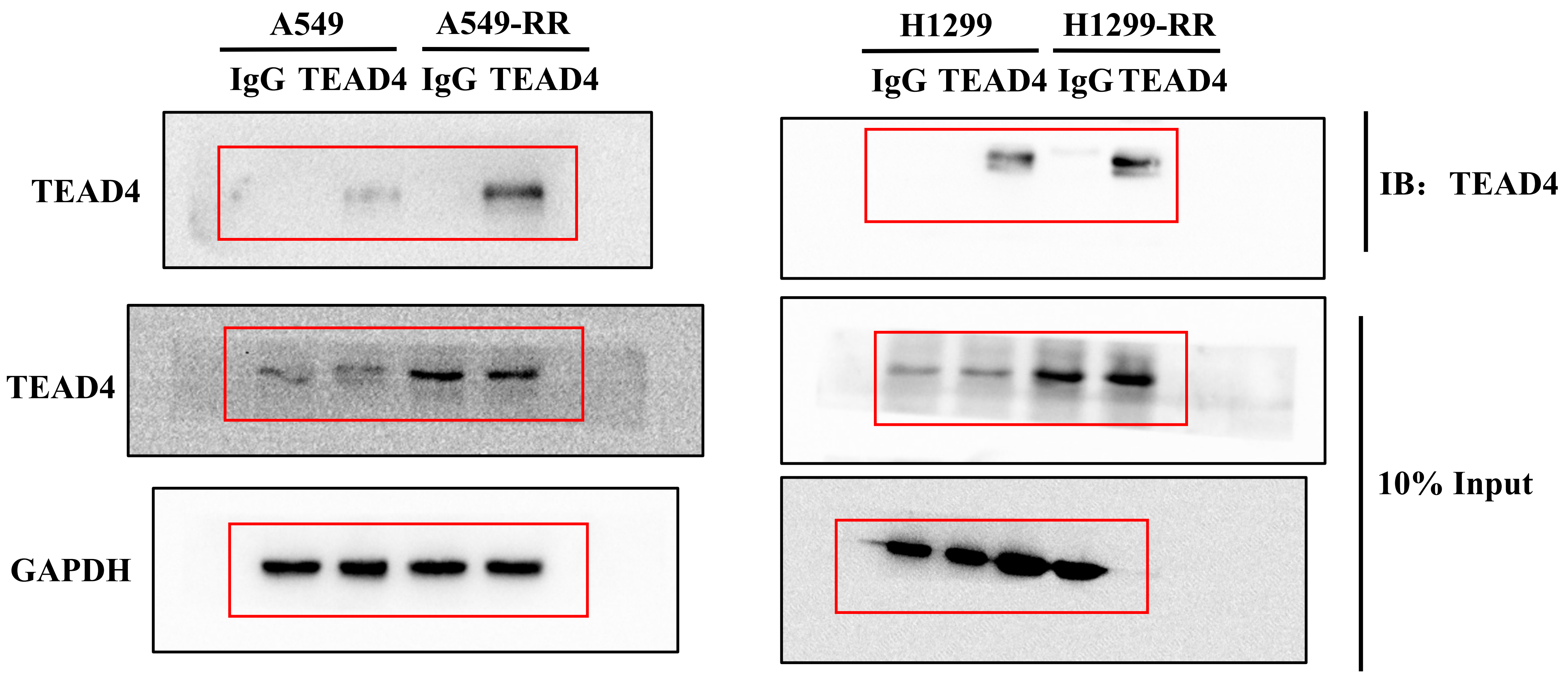
A549
A549-RR
IgG TEAD4 IgG TEAD4
H1299
H1299-RR
IgG TEAD4 IgG TEAD4
TEAD4
TEAD4
GAPDH
IB: TEAD4
10% Input

Fig4.E

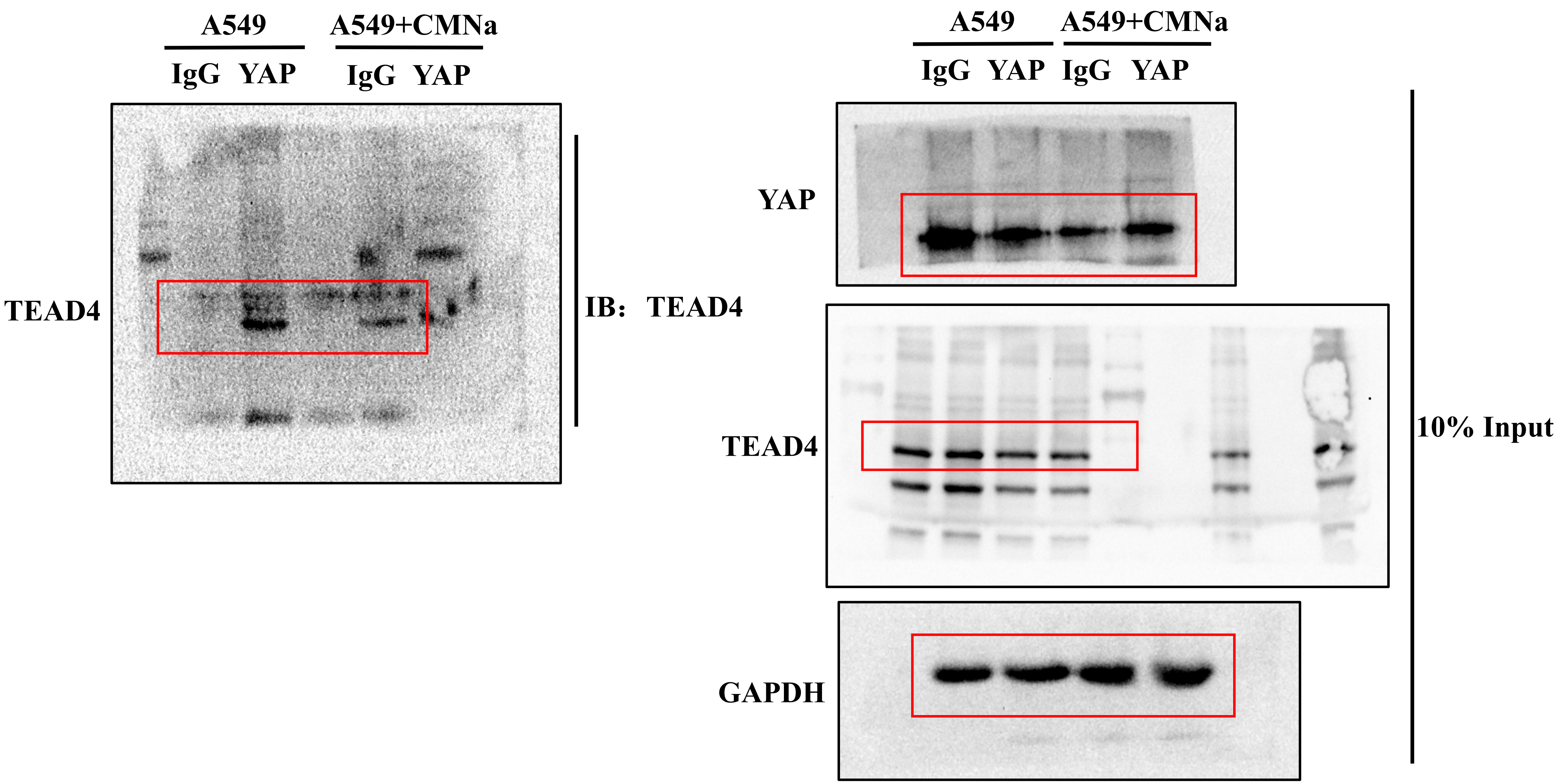
A549
A549+CMNa
IgG YAP IgG YAP
TEAD4
IB：TEAD4
A549
A549+CMNa
IgG YAP IgG YAP
YAP
TEAD4
GAPDH
10% Input

Fig4.E

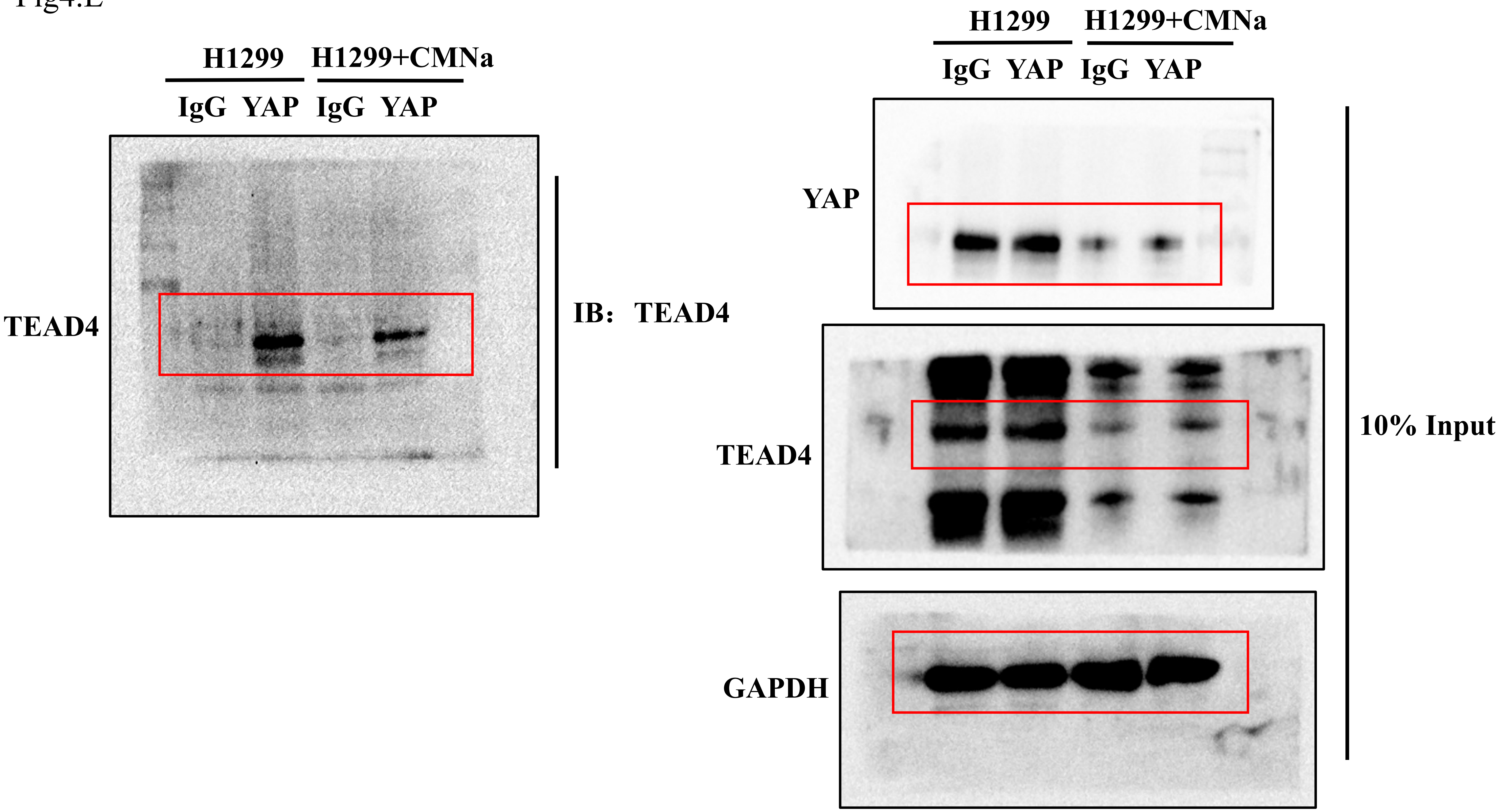

H1299
H1299+CMNa
IgG
YAP
IgG
YAP
TEAD4
IB： TEAD4
H1299
H1299+CMNa
IgG
YAP
IgG
YAP
YAP
TEAD4
GAPDH
10% Input

Fig5.B

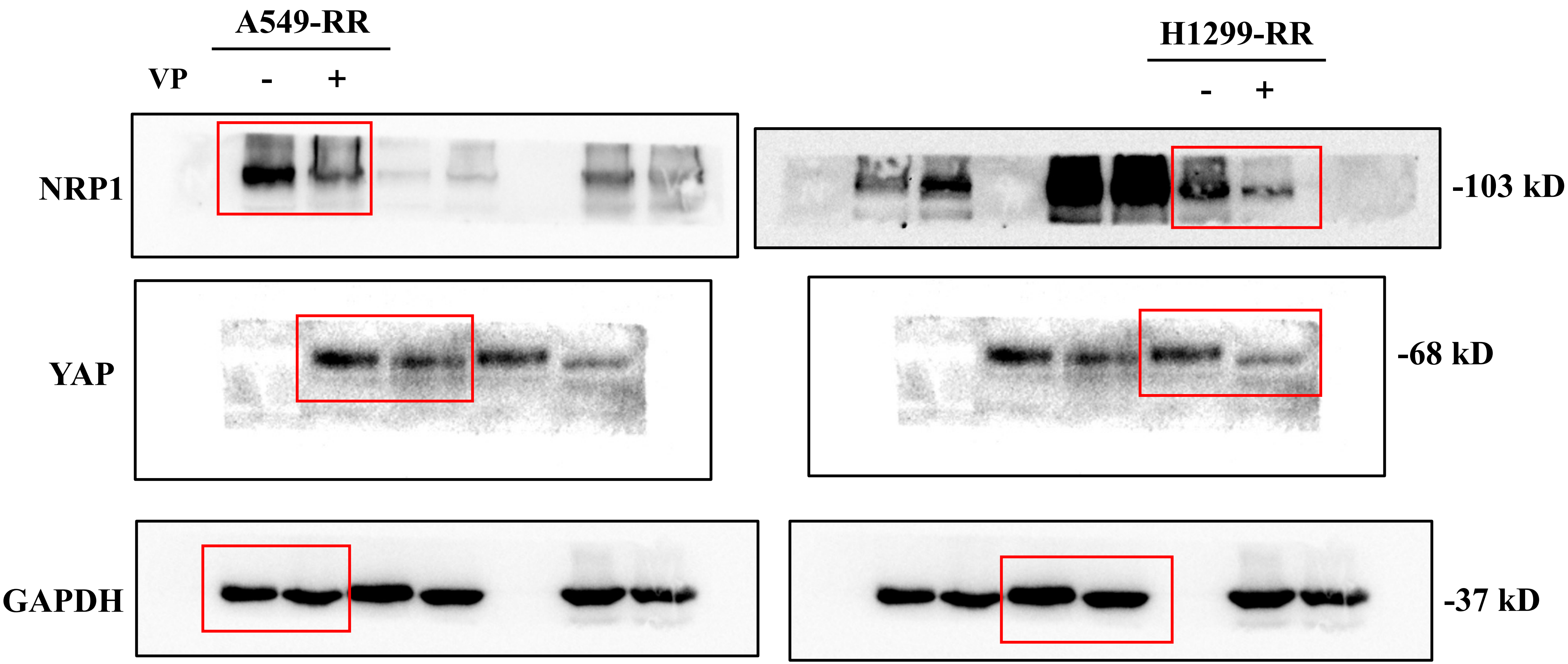

Fig5.B-2

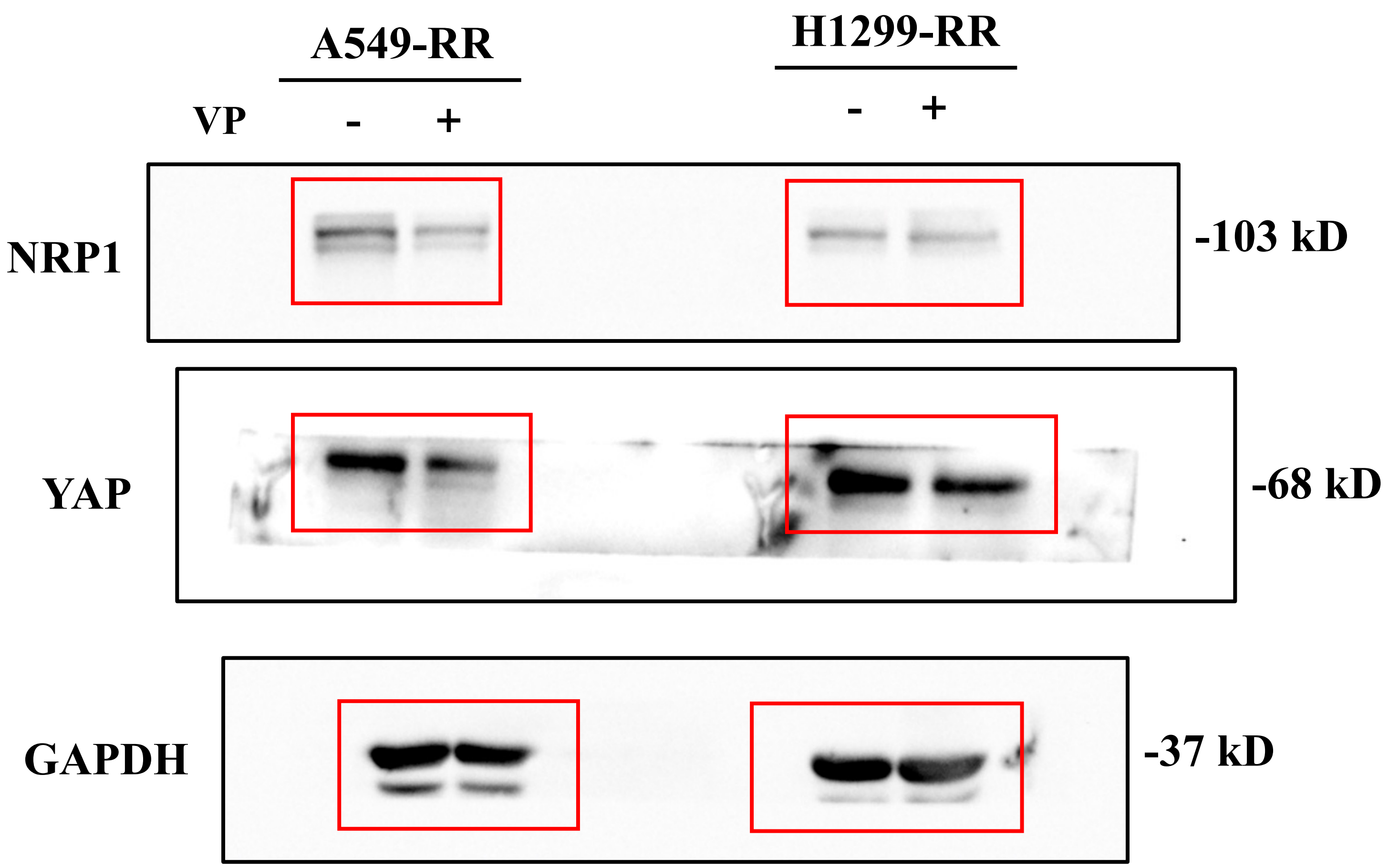

Fig5.B-3

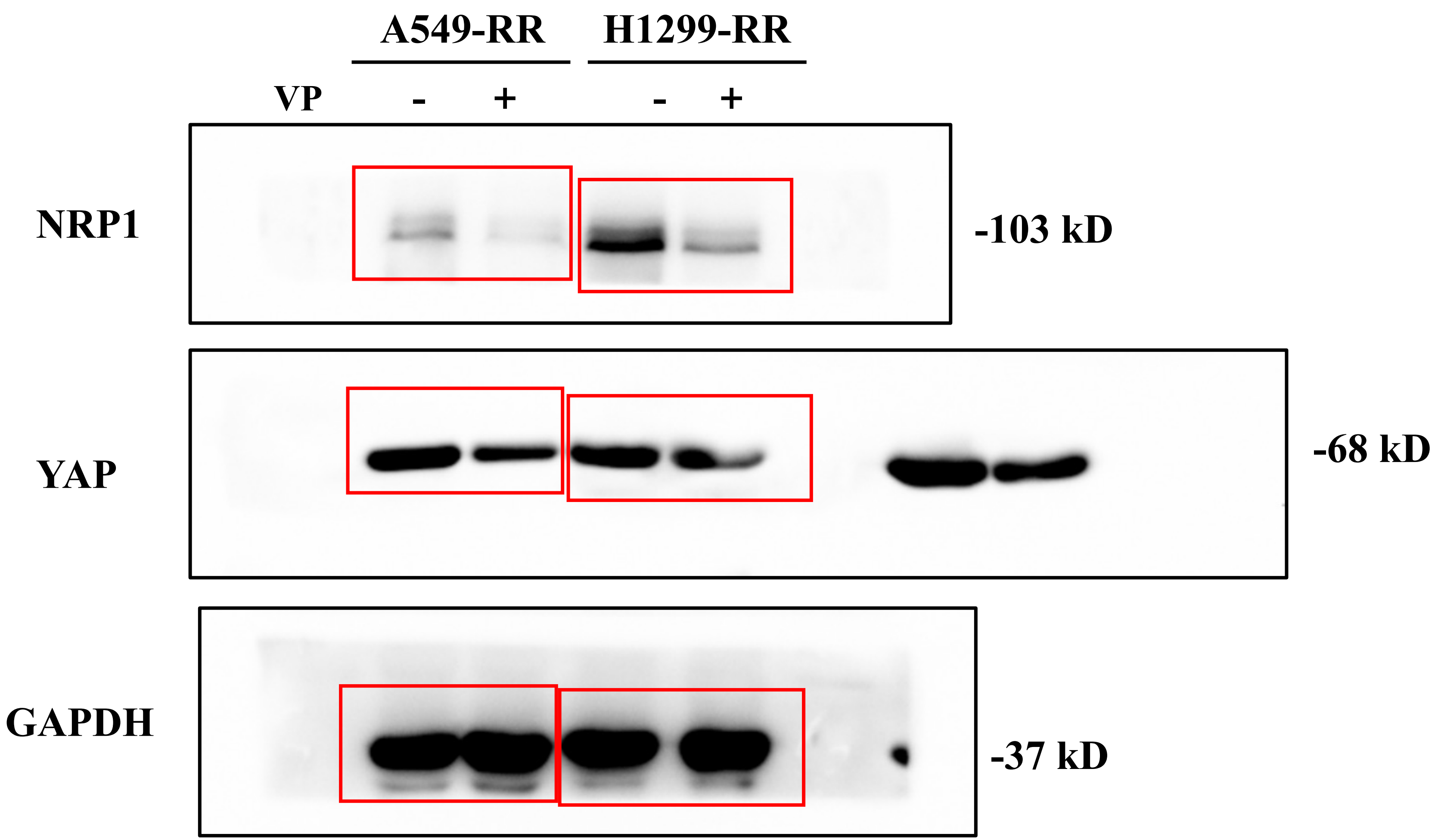

Fig6.B

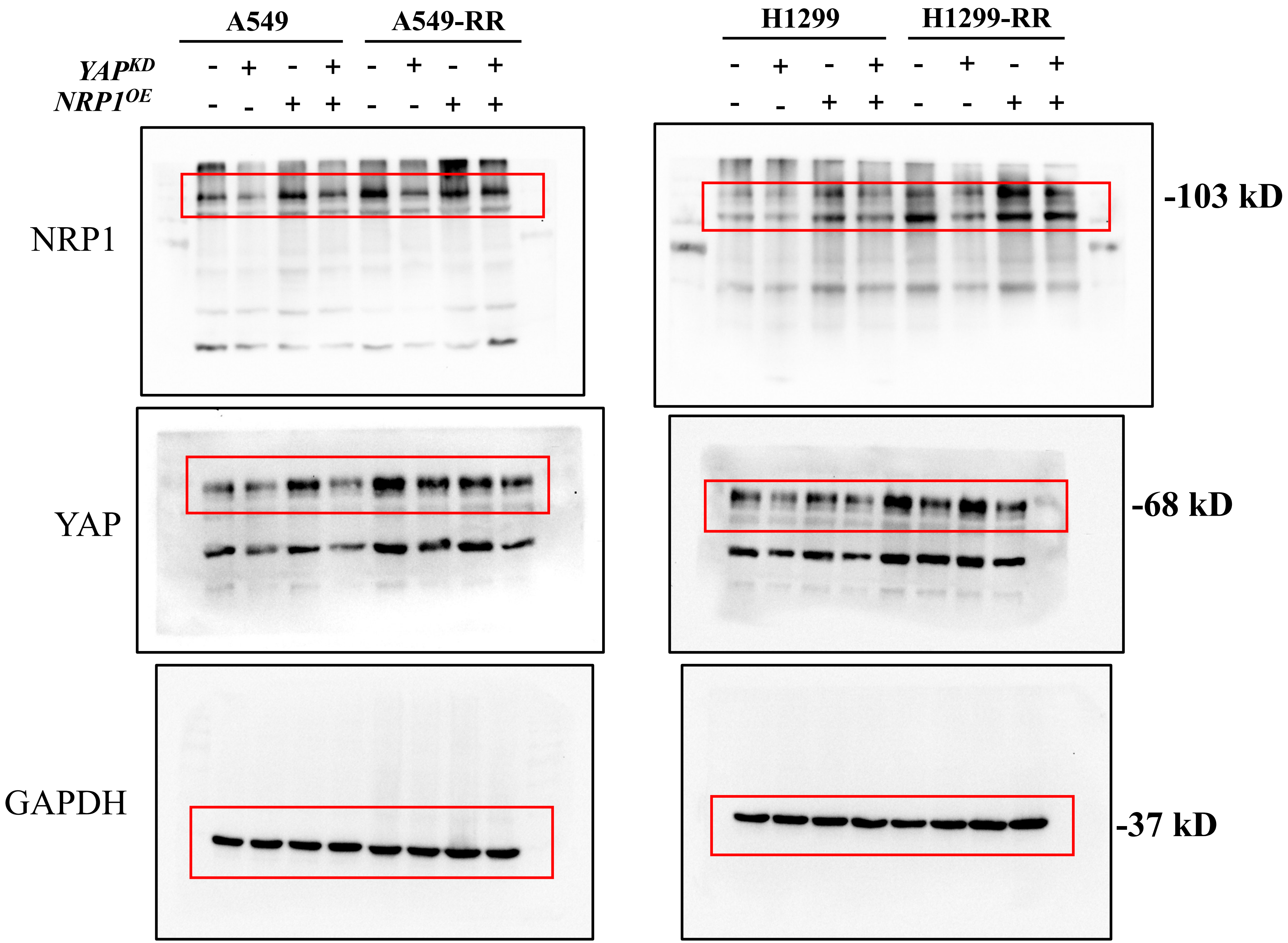
A549
A549-RR
H1299
H1299-RR
YAP^KD - + - + - + - + - + - + - + - +
NRP1^OE - - + + - - + + - - + + - - + +
NRP1
YAP
GAPDH
-103 kD
-68 kD
-37 kD

Fig6.B-2

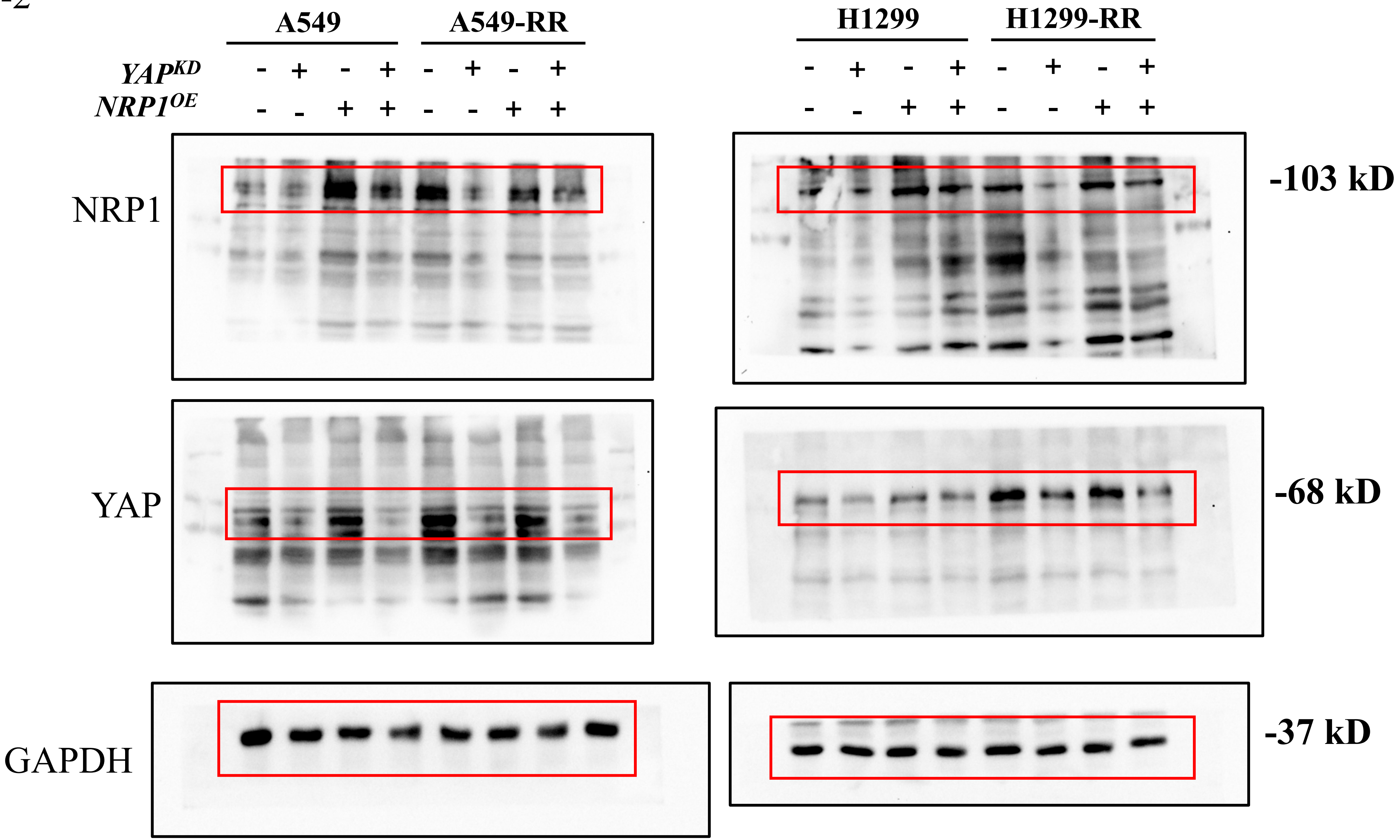
A549
A549-RR
H1299
H1299-RR
YAP^KD
NRP1^OE
- + - + - + - +
- - + + - - + +
- + - + - + - +
- - + + - - + +
NRP1
YAP
GAPDH
-103 kD
-68 kD
-37 kD

Fig6.B-3

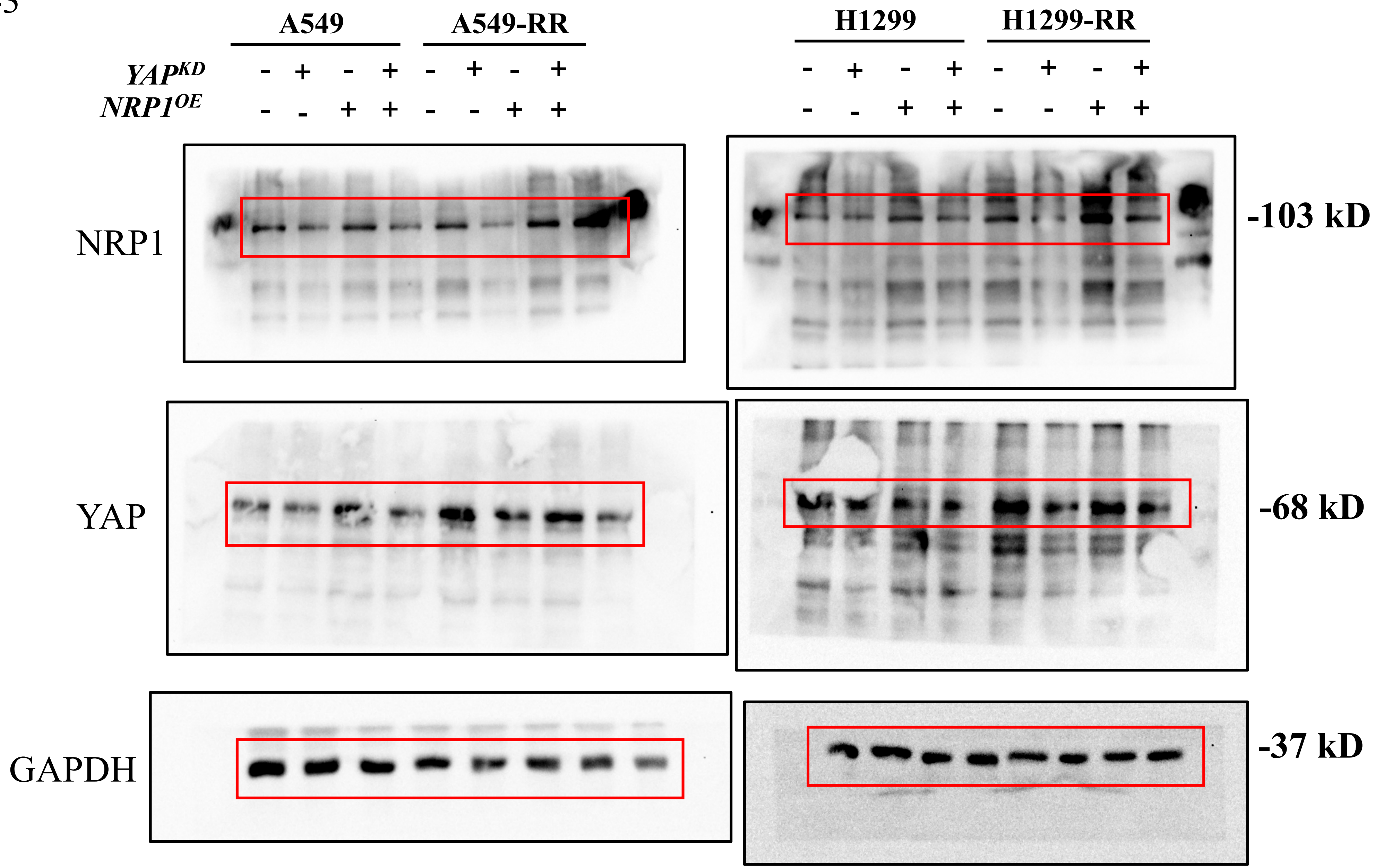

A549
A549-RR
H1299
H1299-RR
YAP^KD - + - + - + - + - + - + - + - +
NRP1^OE - - + + - - + + - - + + - - + +
NRP1
YAP
GAPDH
-103 kD
-68 kD
-37 kD

Fig7.D

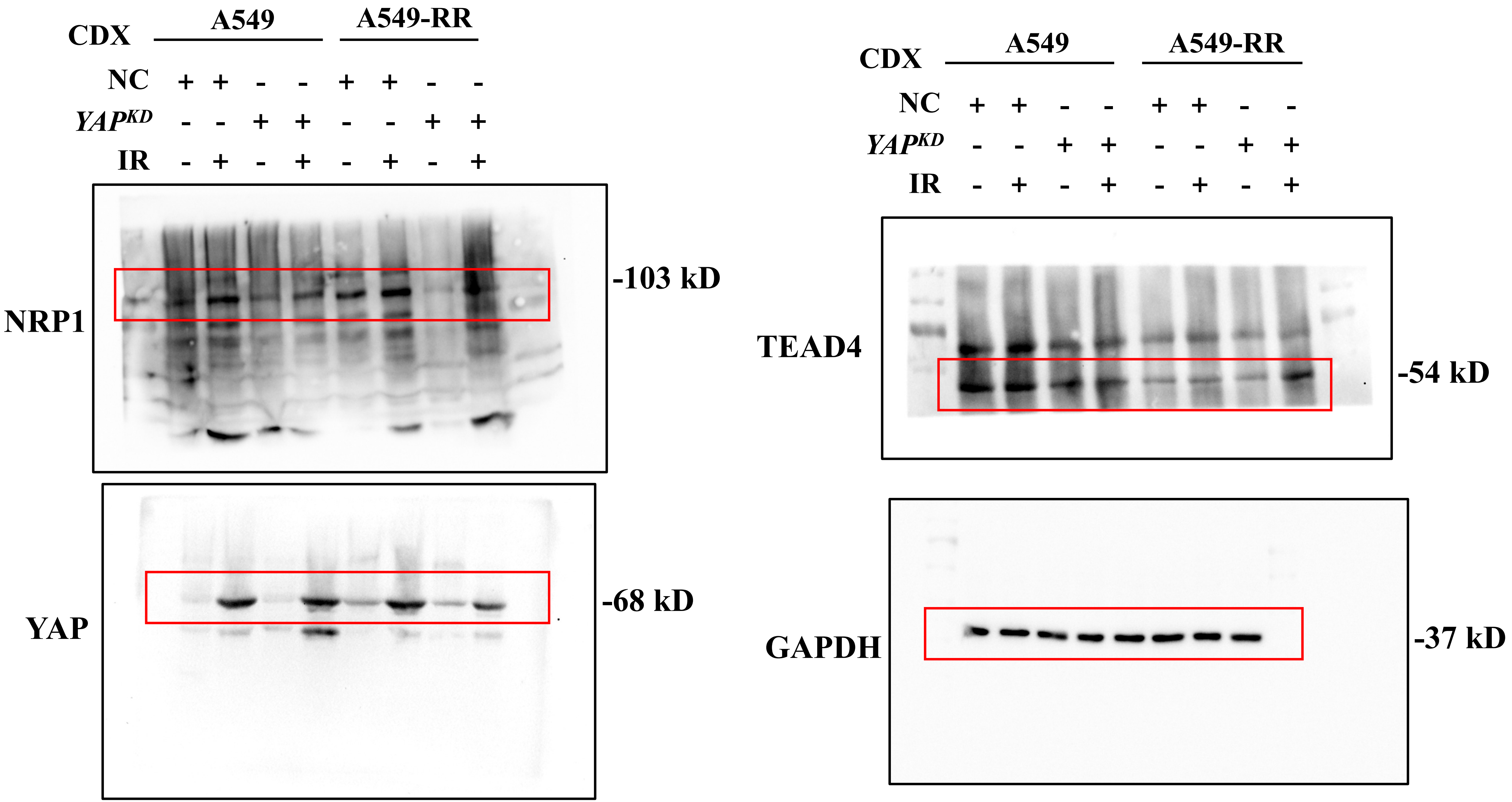

CDX
A549
A549-RR
NC + + - - + + - -
YAPKD - - + + - - + +
IR - + - + - + - +
NRP1
-103 kD
YAP
-68 kD
CDX
A549
A549-RR
NC + + - - + + - -
YAPKD - - + + - - + +
IR - + - + - + - +
TEAD4
-54 kD
GAPDH
-37 kD

Fig7.D-2

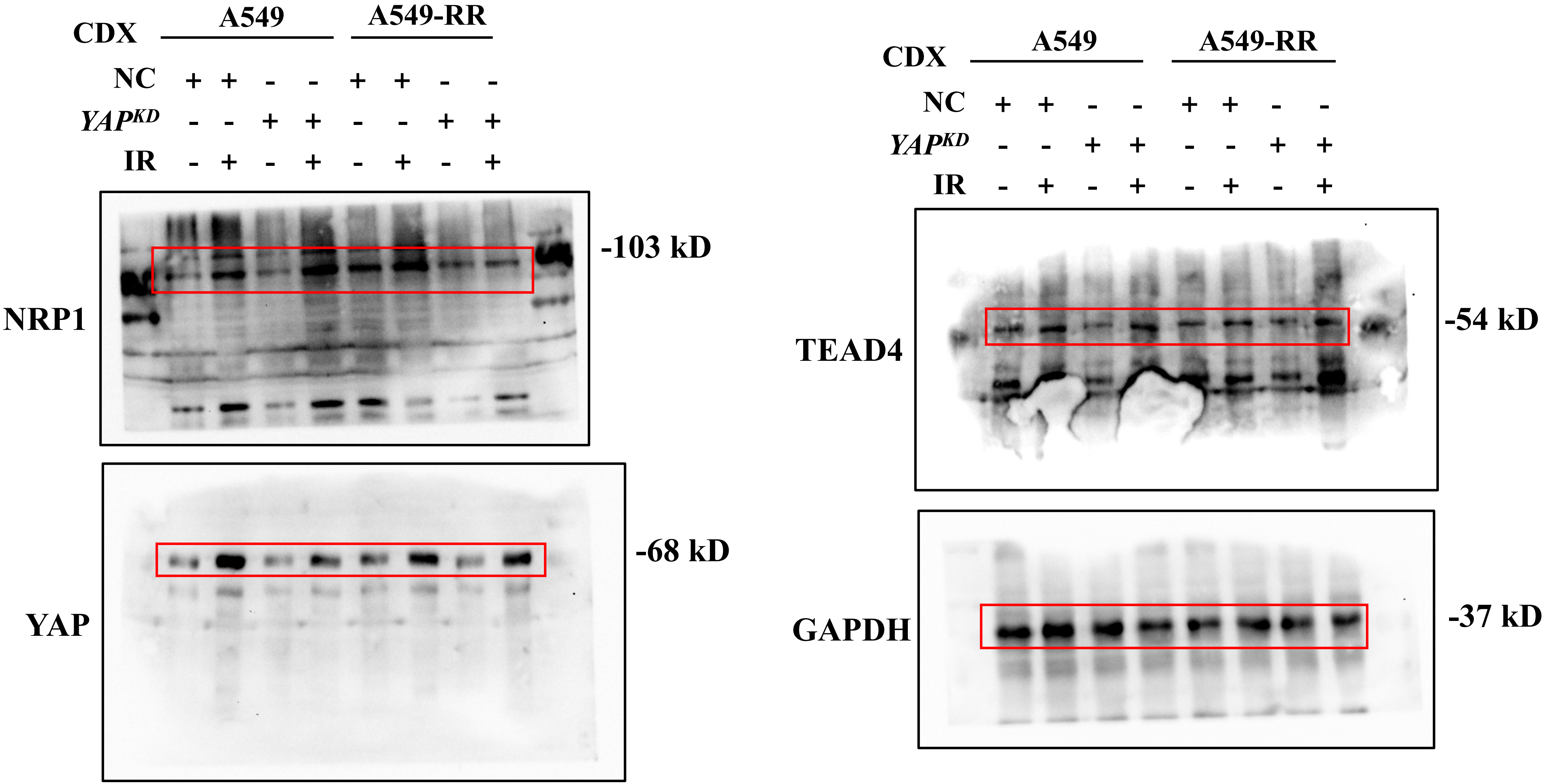
CDX
A549
A549-RR
NC + + - - + + - -
YAPKD - - + + - - + +
IR - + - + - + - +
NRP1
-103 kD
YAP
-68 kD
CDX
A549
A549-RR
NC + + - - + + - -
YAPKD - - + + - - + +
IR - + - + - + - +
TEAD4
-54 kD
GAPDH
-37 kD

Fig7.D-3

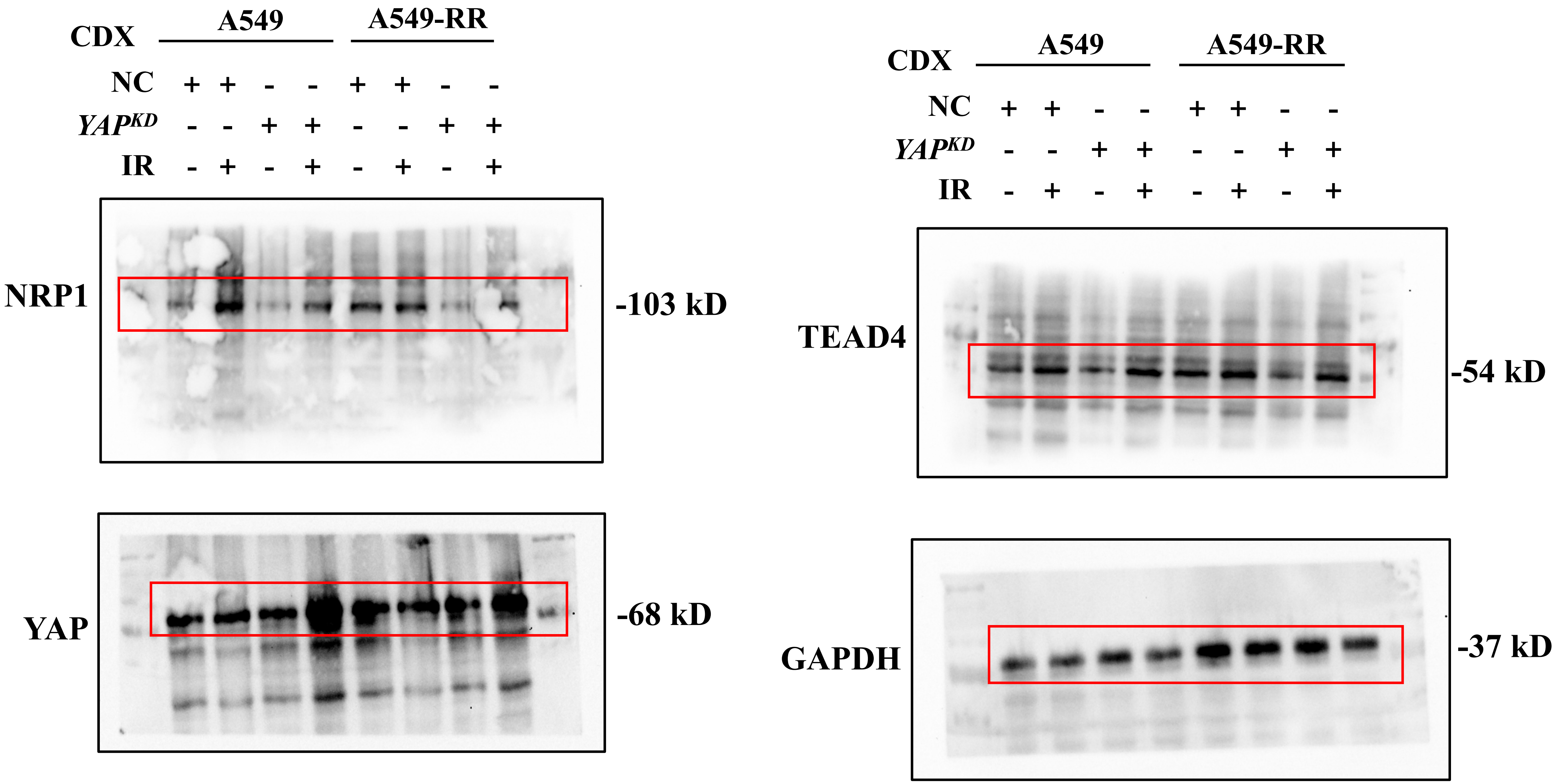
CDX
A549
A549-RR
NC + + - - + + - -
YAPKD - - + + - - + +
IR - + - + - + - +
NRP1
-103 kD
YAP
-68 kD
CDX
A549
A549-RR
NC + + - - + + - -
YAPKD - - + + - - + +
IR - + - + - + - +
TEAD4
-54 kD
GAPDH
-37 kD

SFig1.C

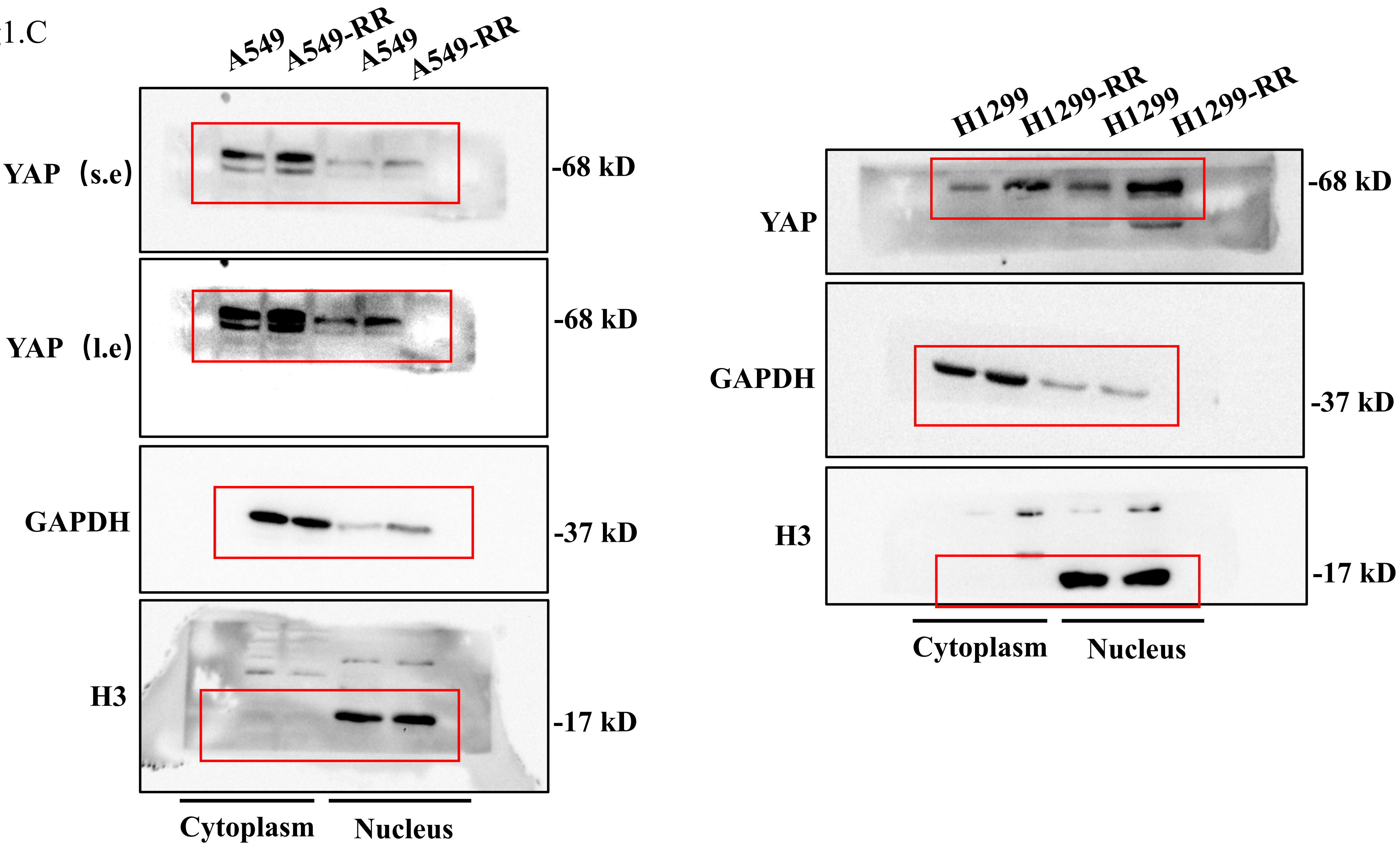
A549
A549-RR
A549
A549-RR
YAP (s.e)
-68 kD
YAP (l.e)
-68 kD
GAPDH
-37 kD
H3
-17 kD
Cytoplasm
Nucleus
H1299
H1299-RR
H1299
H1299-RR
YAP
-68 kD
GAPDH
-37 kD
H3
-17 kD
Cytoplasm
Nucleus

SFig3.C

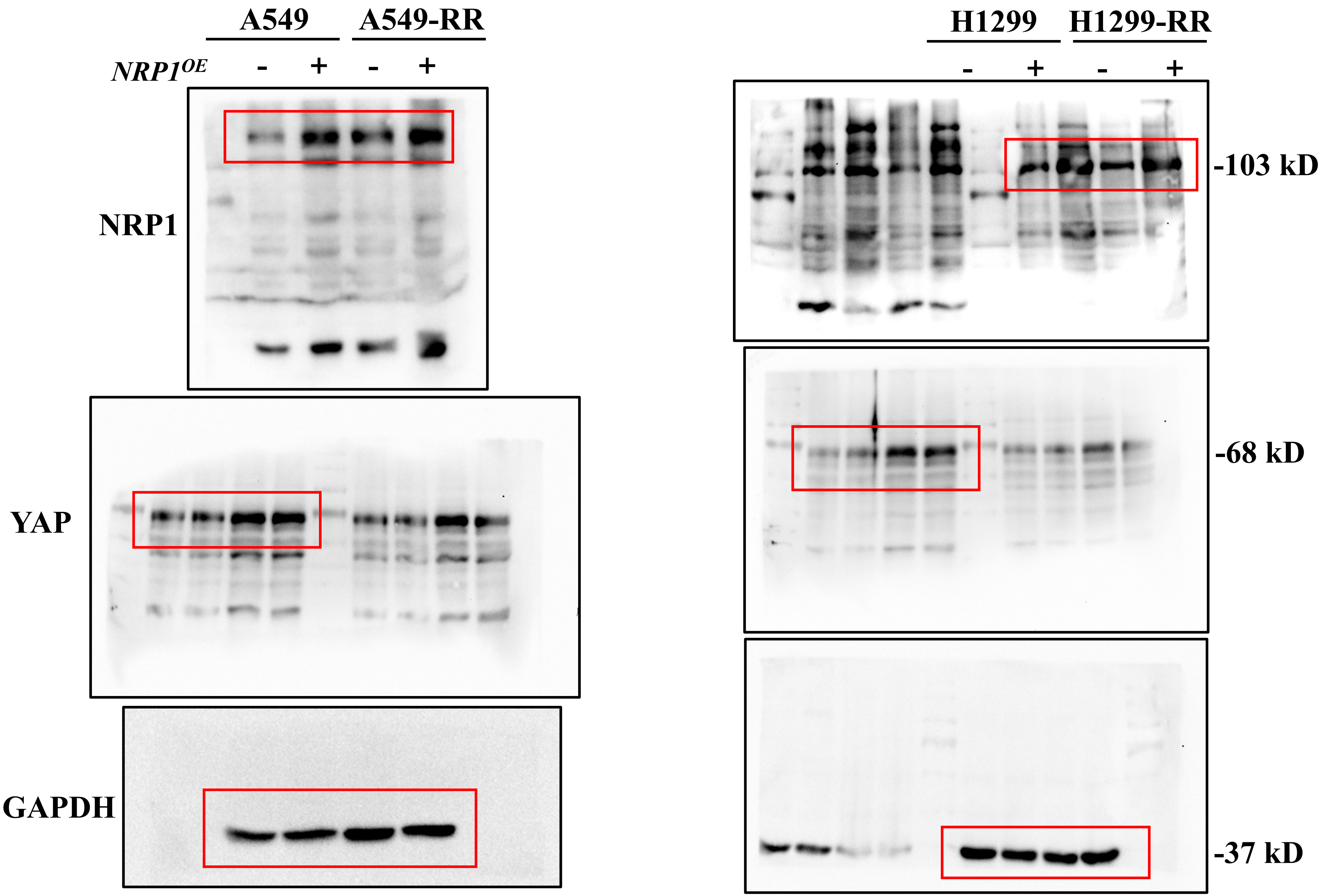
A549
A549-RR
NRP1OE
- + - +
NRP1
YAP
GAPDH
H1299
H1299-RR
- + - +
-103 kD
-68 kD
-37 kD

SFig3.C-2

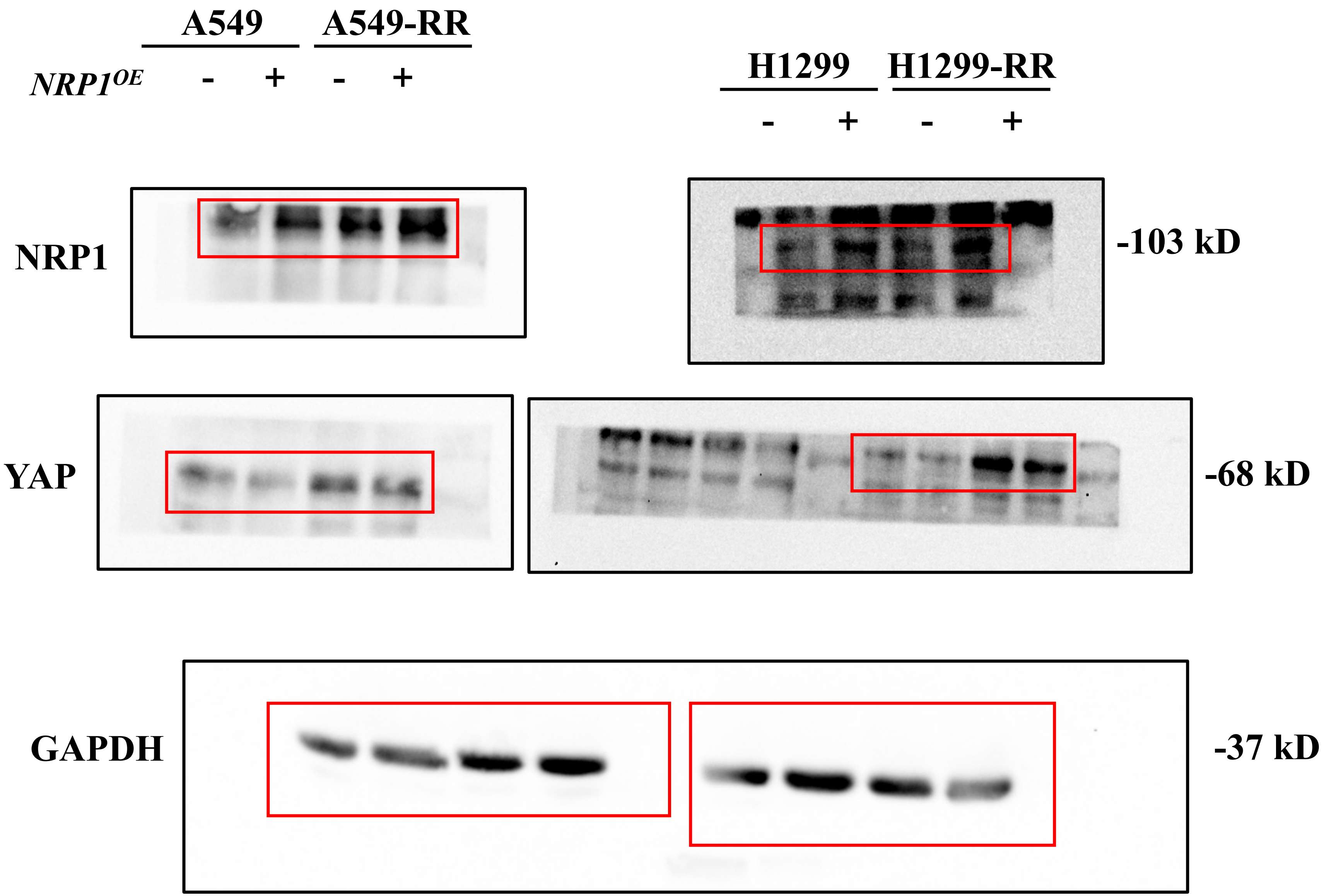
A549
A549-RR
NRP1^OE
-
+
-
+
H1299
H1299-RR
-
+
-
+
NRP1
-103 kD
YAP
-68 kD
GAPDH
-37 kD

SFig3.C-3

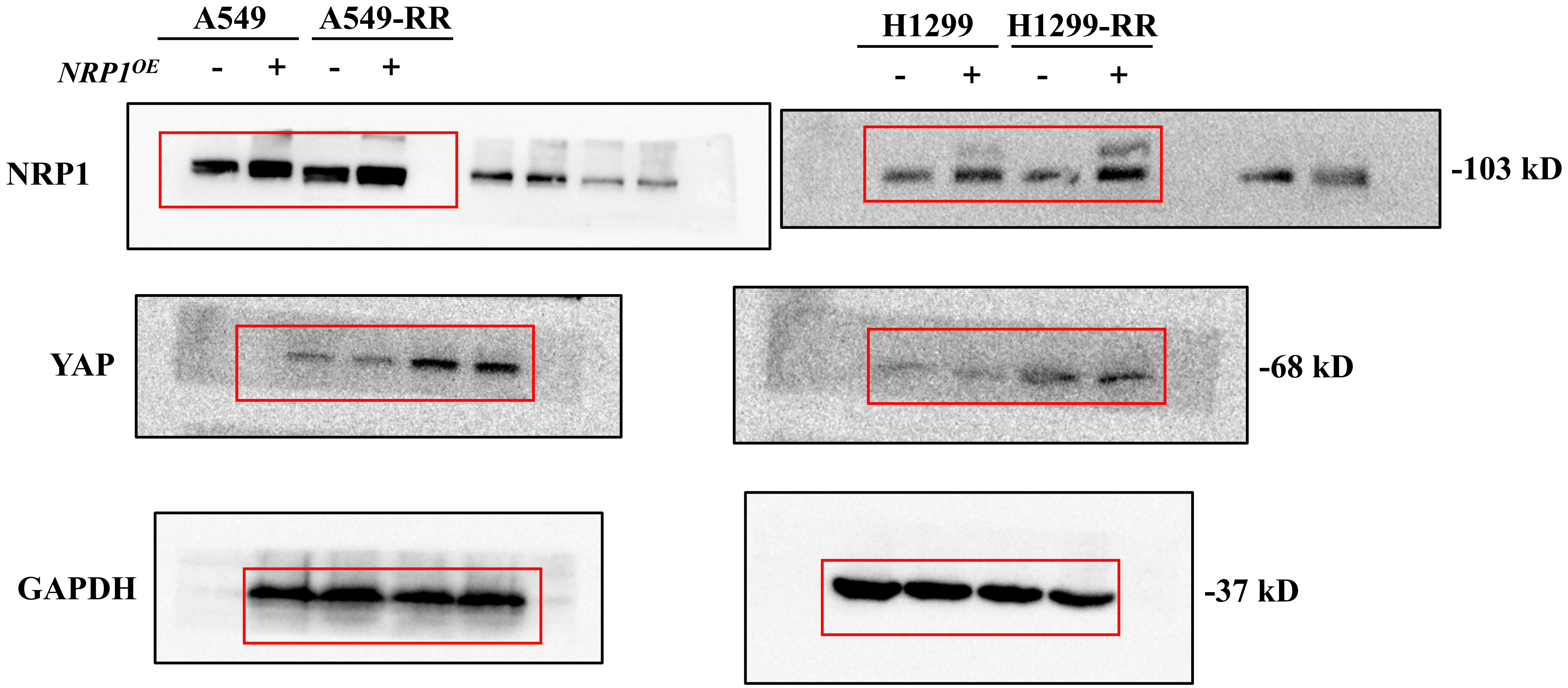
A549
A549-RR
NRP1OE
- + - +
H1299
H1299-RR
- + - +
NRP1
YAP
GAPDH
-103 kD
-68 kD
-37 kD

SFig3.C

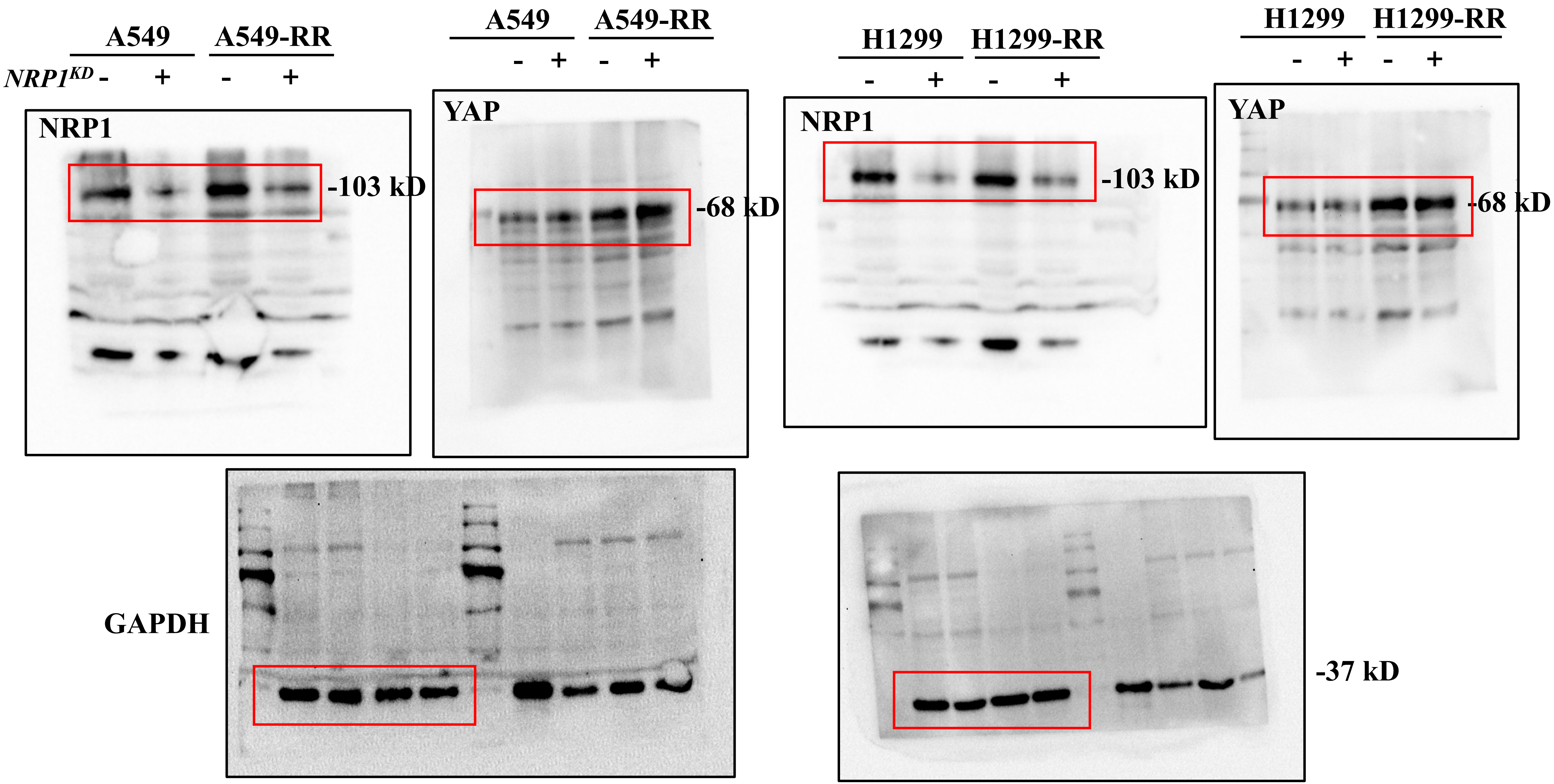

A549
A549-RR
NRP1KD - + - +
NRP1
-103 kD
A549
A549-RR
- + - +
YAP
-68 kD
H1299
H1299-RR
- + - +
NRP1
-103 kD
H1299
H1299-RR
- + - +
YAP
-68 kD
GAPDH
-37 kD

SFig3.C-2

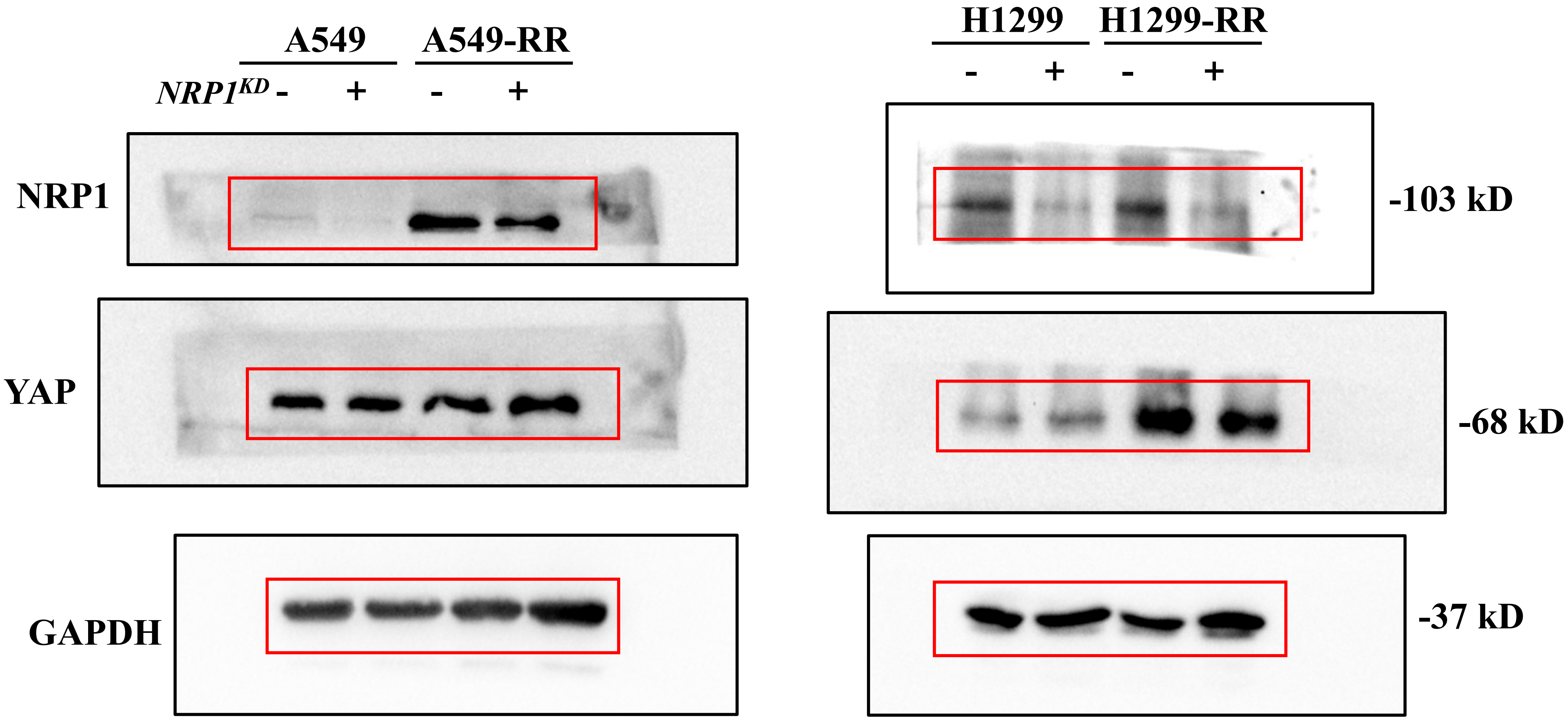
A549
A549-RR
NRP1KD - + - +
H1299
H1299-RR
- + - +
NRP1
YAP
GAPDH
-103 kD
-68 kD
-37 kD

SFig3.C-3

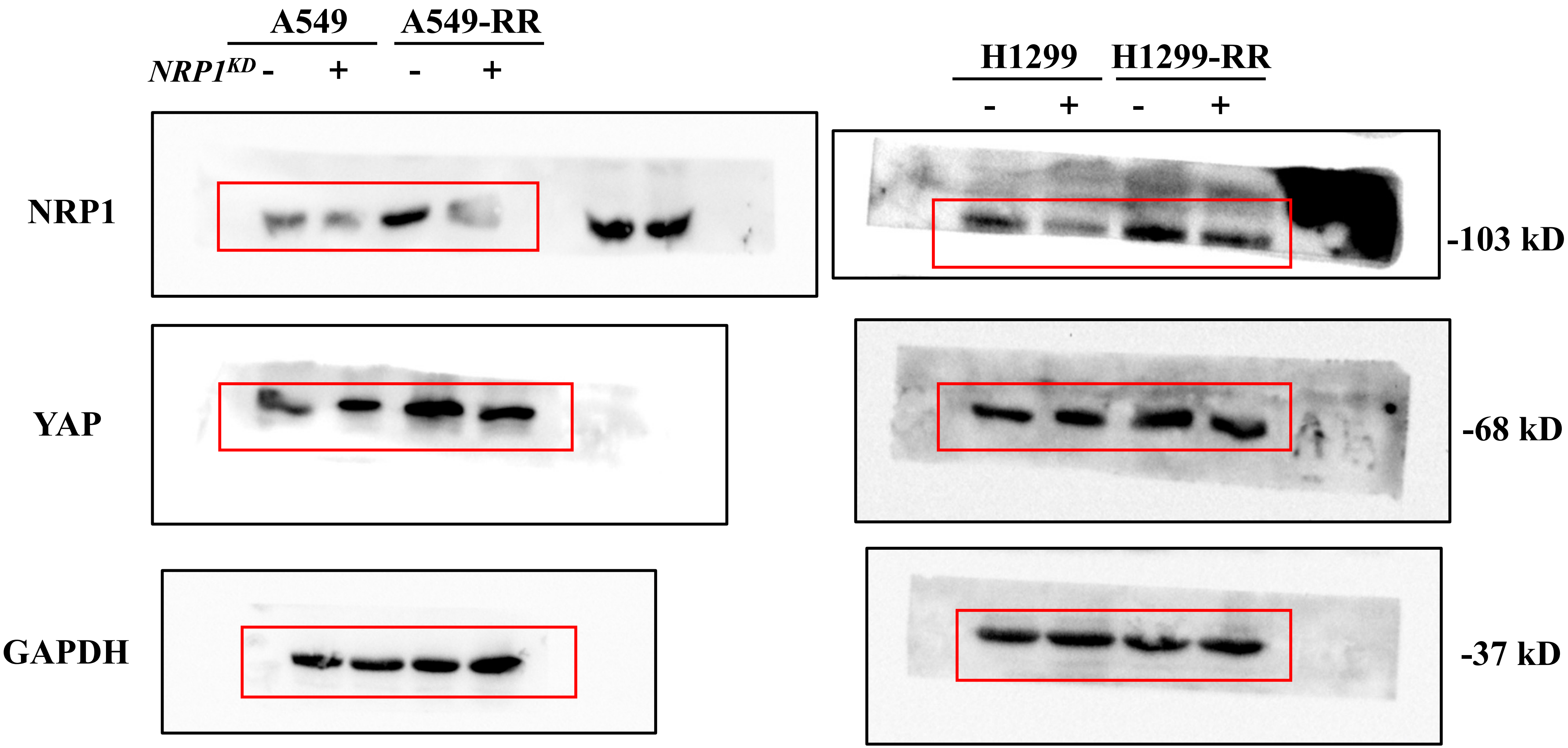

A549
A549-RR
NRP1KD - + - +
H1299
H1299-RR
- + - +
NRP1
YAP
GAPDH
-103 kD
-68 kD
-37 kD

SFig.4D

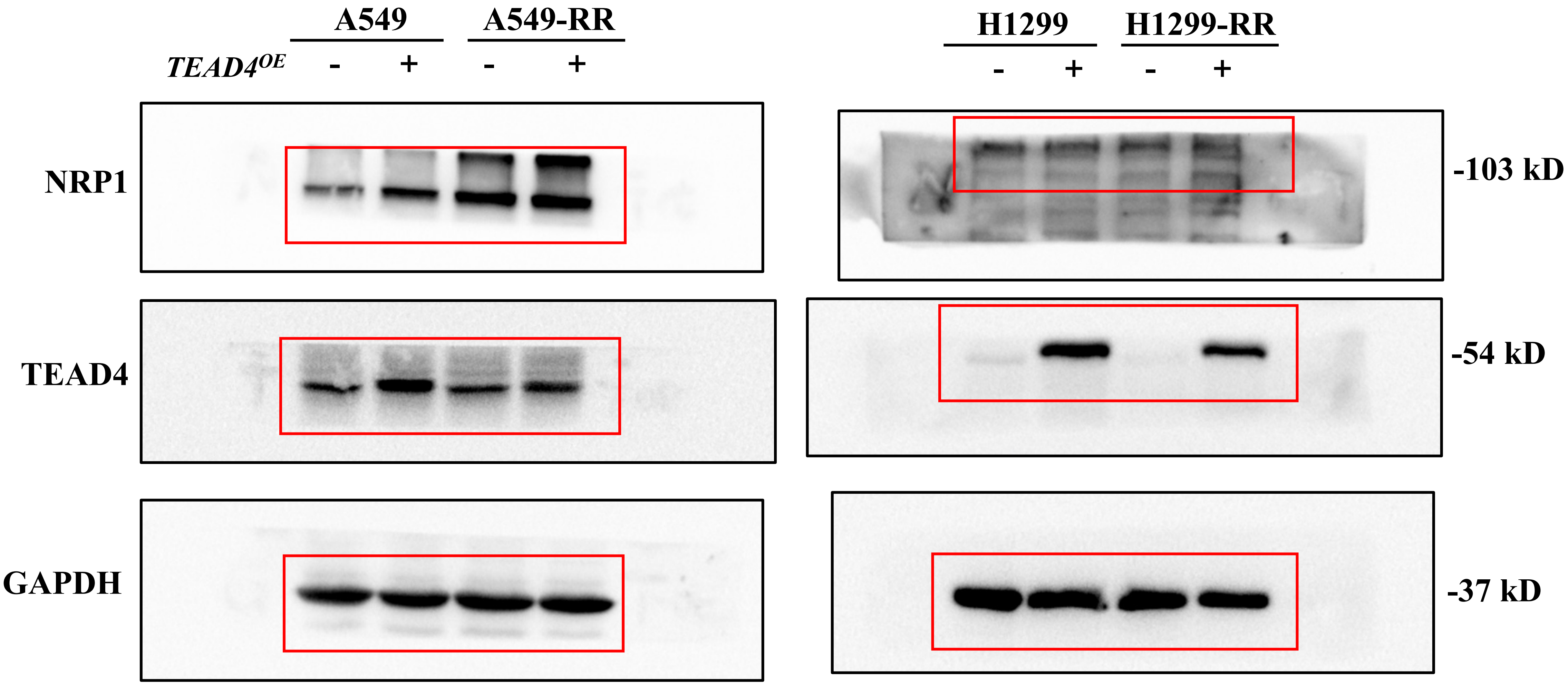
A549
A549-RR
TEAD4OE
- + - +
H1299
H1299-RR
- + - +
NRP1
TEAD4
GAPDH
-103 kD
-54 kD
-37 kD

SFig.4D-2

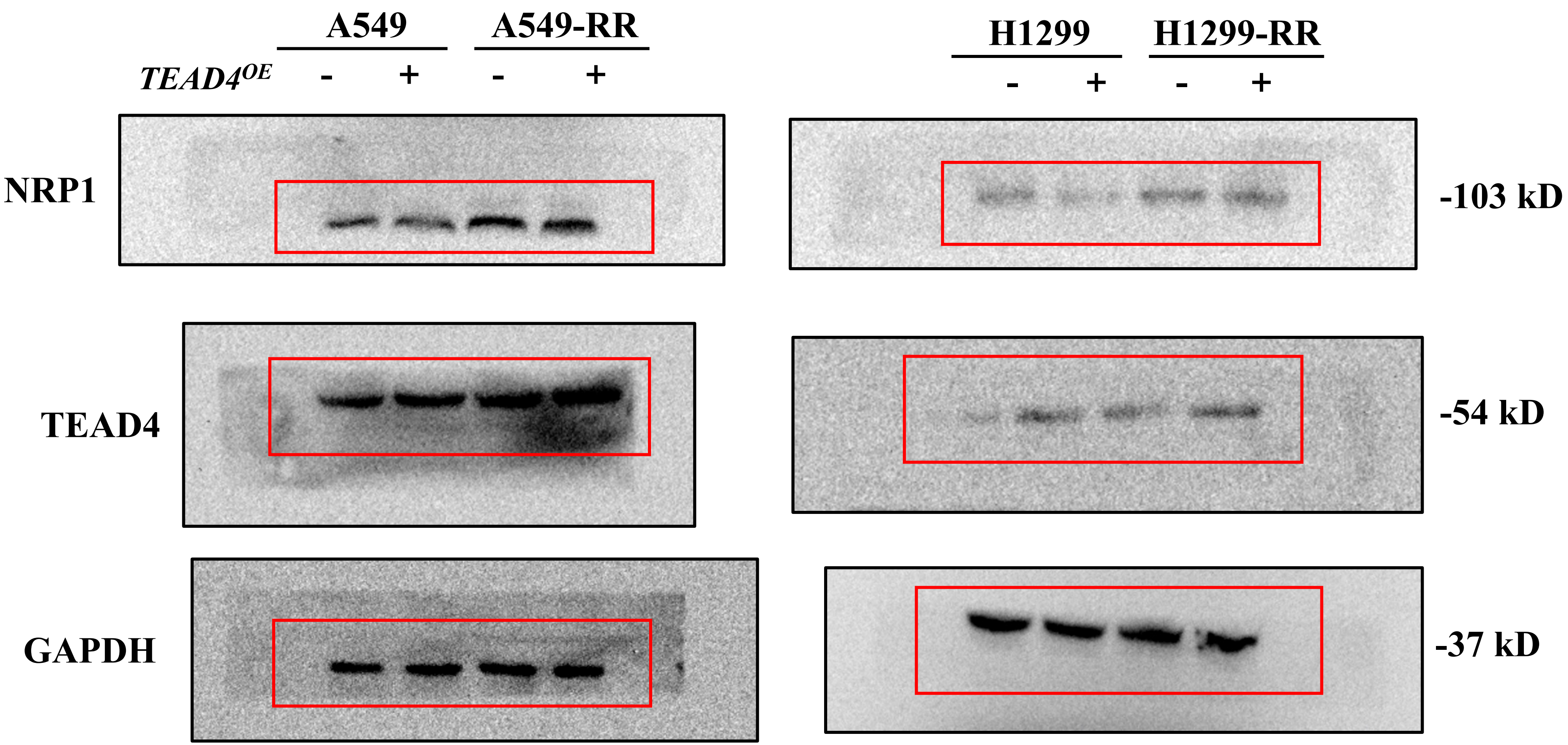
A549
A549-RR
TEAD4OE
- + - +
H1299
H1299-RR
- + - +
NRP1
TEAD4
GAPDH
-103 kD
-54 kD
-37 kD

SFig.4D-3

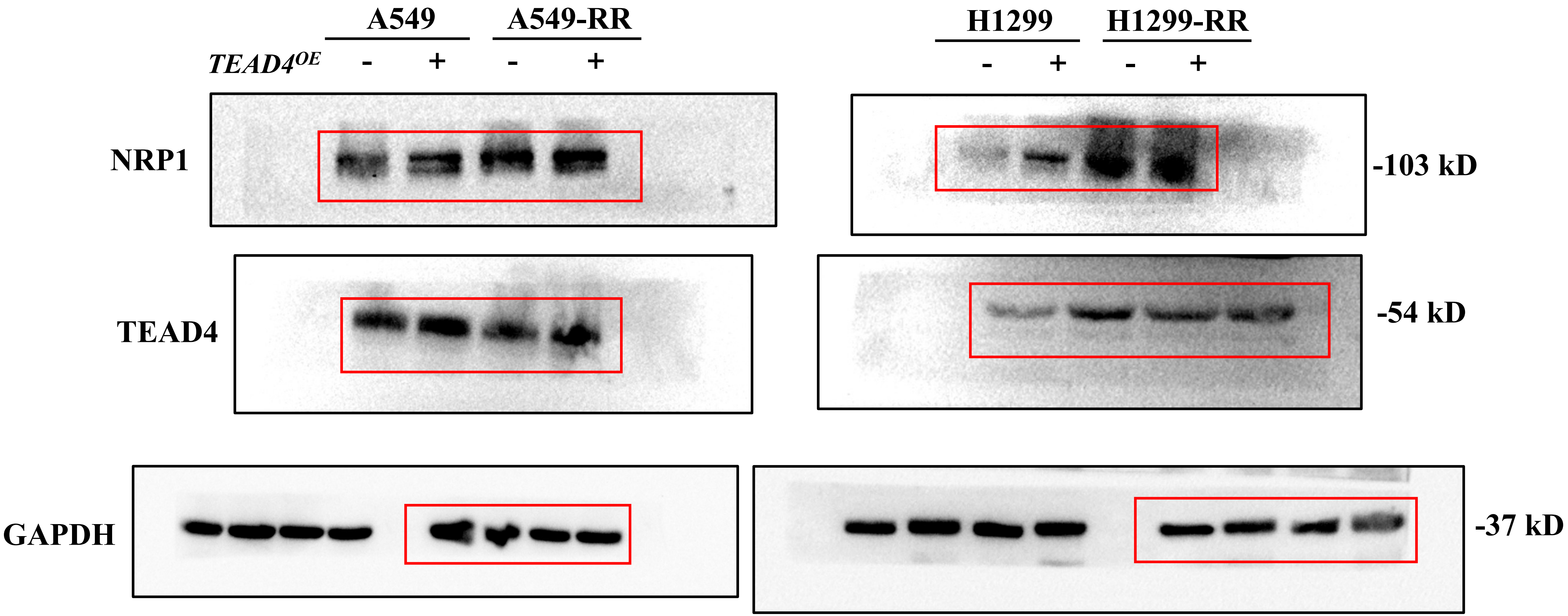
A549
A549-RR
H1299
H1299-RR
TEAD4OE
- + - +
- + - +
NRP1
TEAD4
GAPDH
-103 kD
-54 kD
-37 kD

SFig.4D

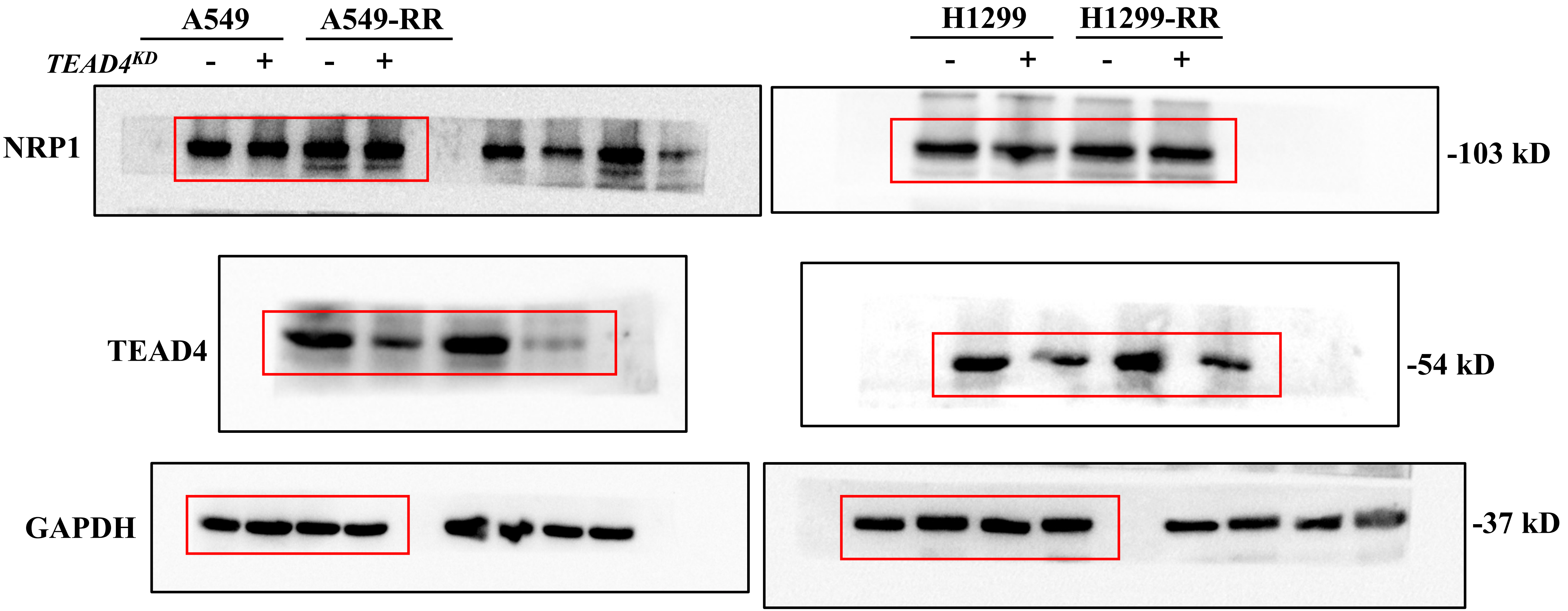

A549
A549-RR
H1299
H1299-RR
*TEAD4^KD^*
- + - +
- + - +
NRP1
TEAD4
GAPDH
-103 kD
-54 kD
-37 kD

SFig.4D-2

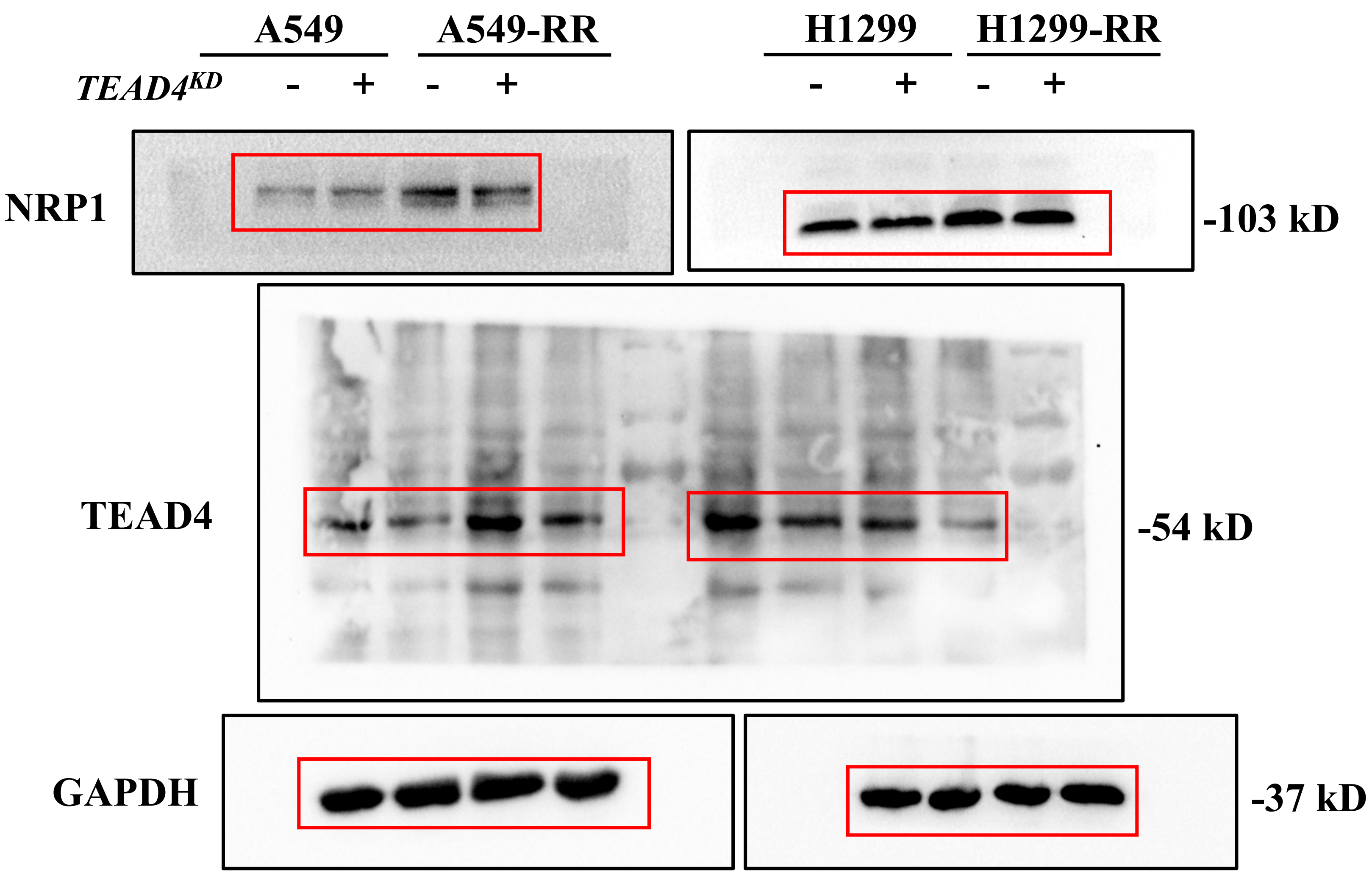
A549
A549-RR
H1299
H1299-RR
TEAD4KD - + - + - + - +
NRP1
-103 kD
TEAD4
-54 kD
GAPDH
-37 kD

SFig.4D-3

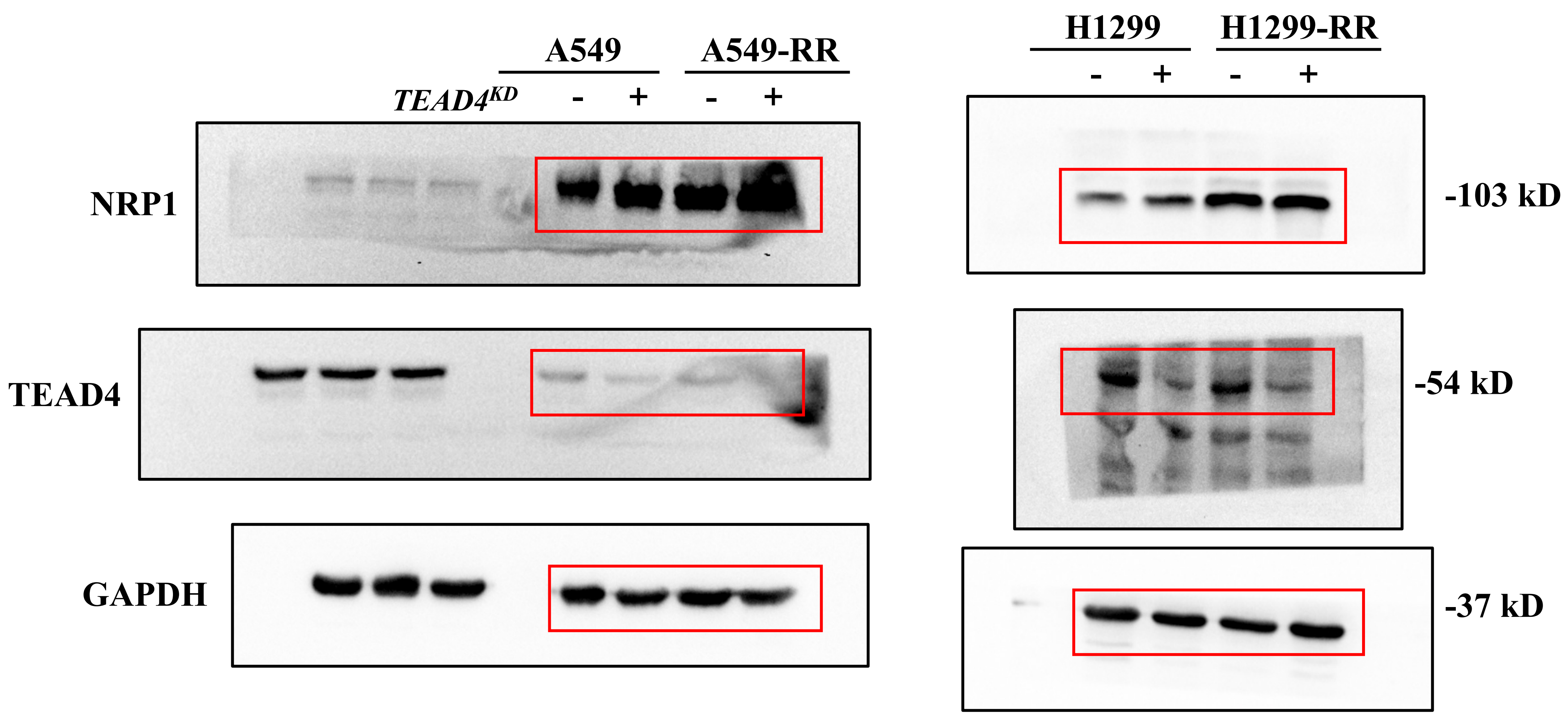
A549
A549-RR
TEAD4KD
- + - +
NRP1
TEAD4
GAPDH
H1299
H1299-RR
- + - +
-103 kD
-54 kD
-37 kD

SFig4.F

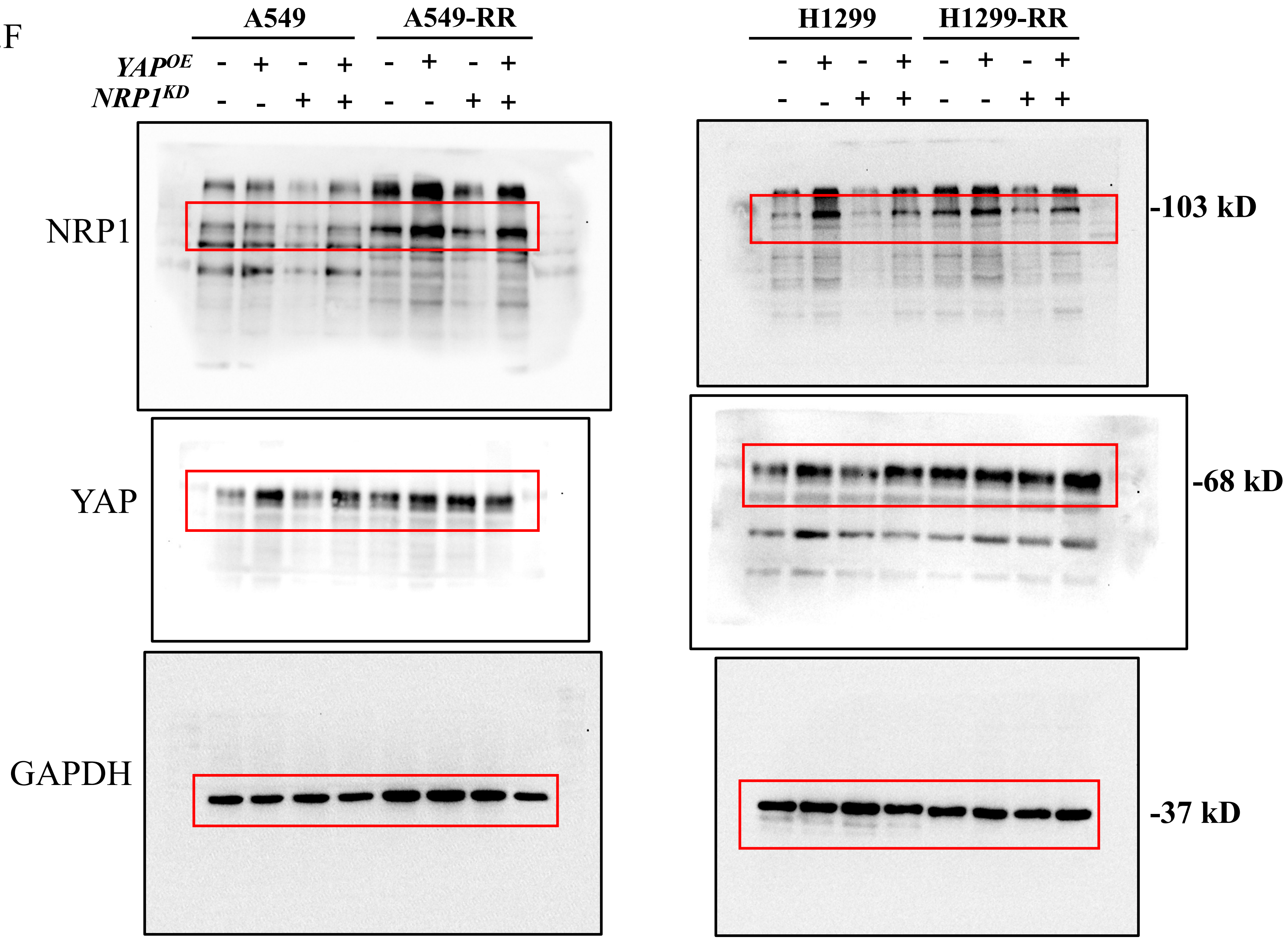
A549
A549-RR
H1299
H1299-RR
YAP^OE
NRP1^KD
NRP1
YAP
GAPDH
-103 kD
-68 kD
-37 kD

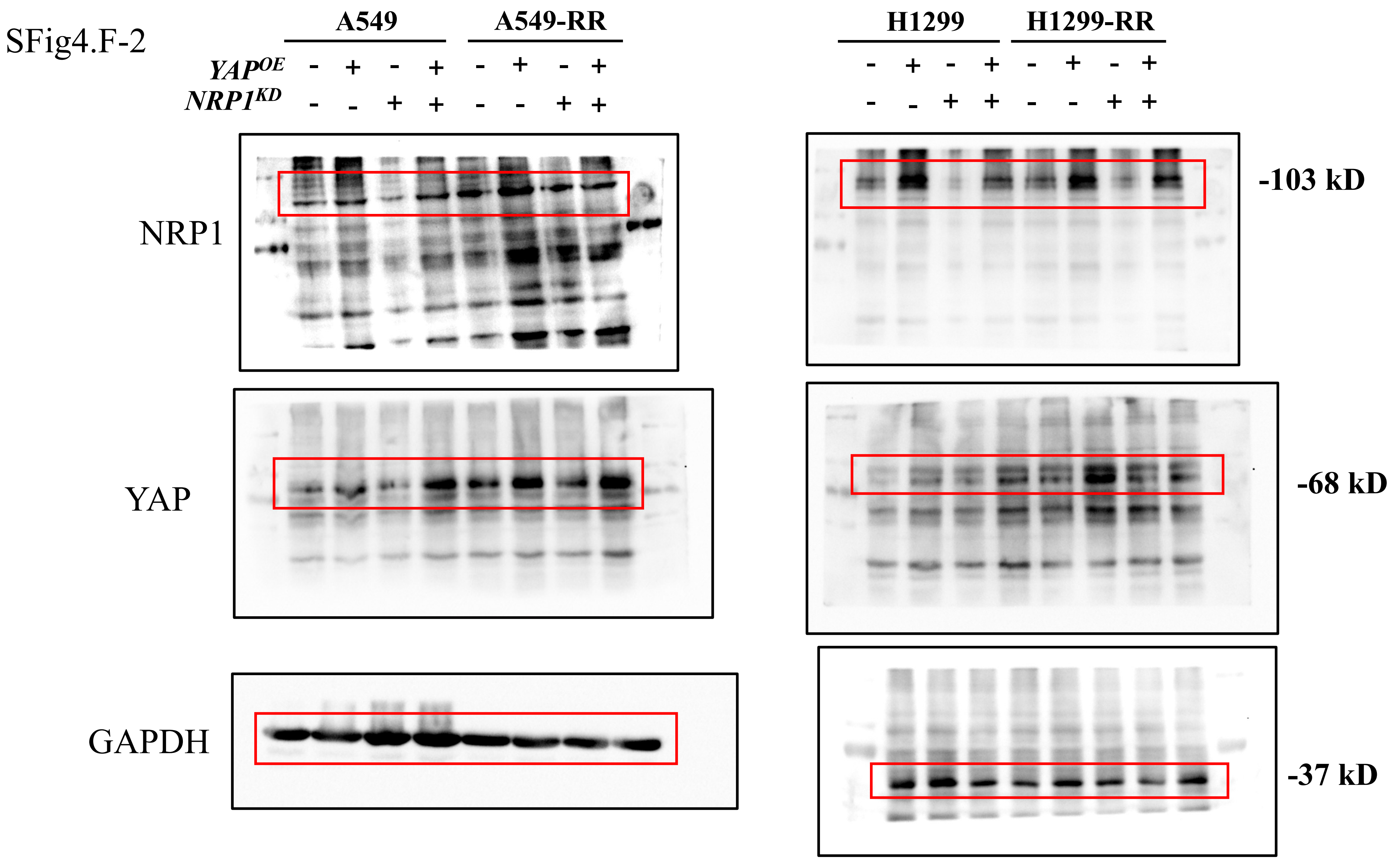
SFig4.F-2
A549
A549-RR
H1299
H1299-RR
YAP^OE
NRP1^KD
NRP1
YAP
GAPDH
-103 kD
-68 kD
-37 kD

SFig4.F-3

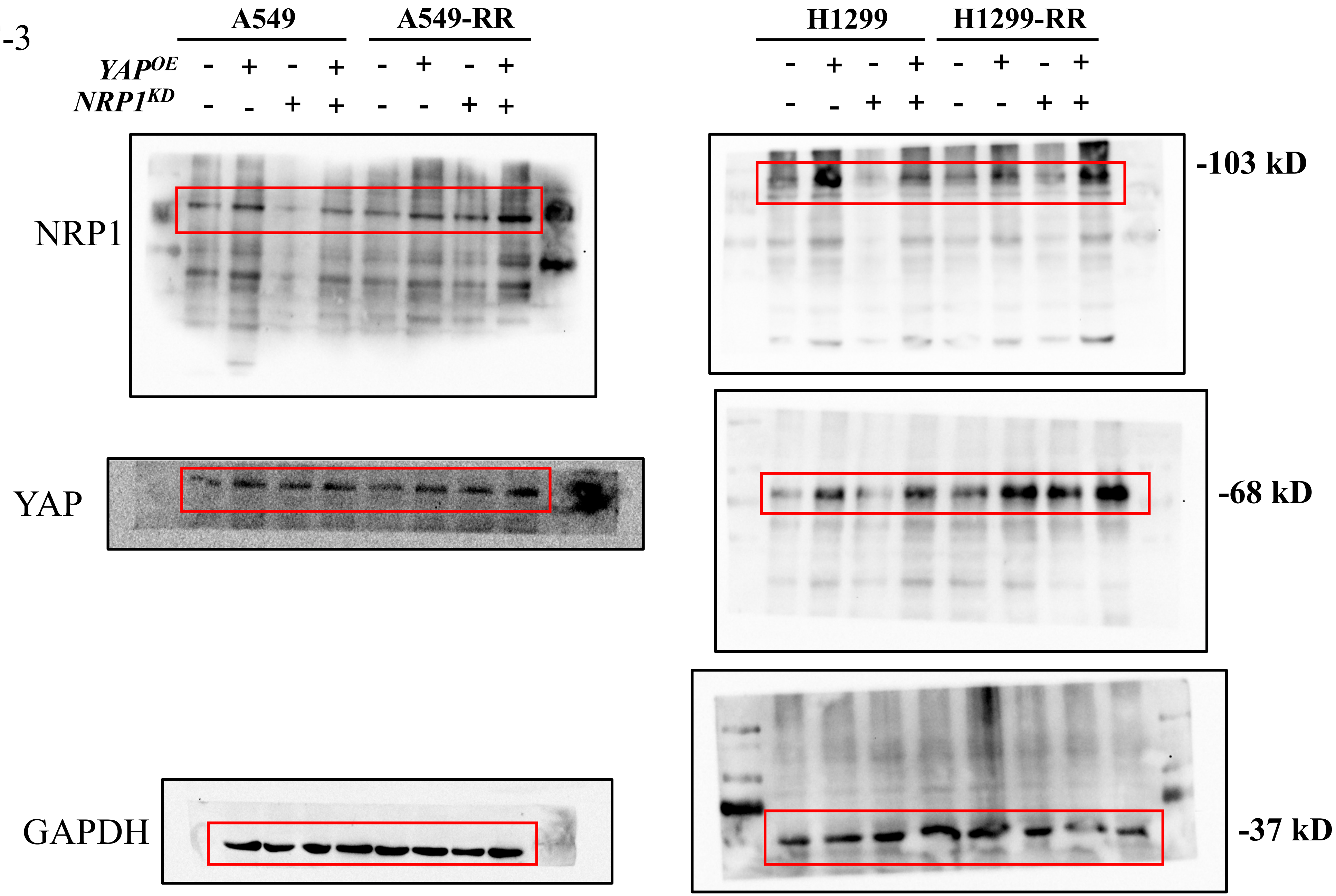
A549
A549-RR
H1299
H1299-RR
YAP^OE
NRP1^KD
- + - + - + - +
- - + + - - + +
NRP1
YAP
GAPDH
-103 kD
-68 kD
-37 kD
